# Engineered haptoglobin β fusion protein targets myoglobin and ameliorates rhabdomyolysis-associated acute kidney injury

**DOI:** 10.1038/s44321-026-00454-0

**Published:** 2026-05-25

**Authors:** Ning Li, Yuru Wang, Lu Han, Ou Qiao, Xinyue Wang, Herui Hao, Xin Chen, Pengtao Wang, Sania Saeed, Jing Wang, Fengjiao Bao, Yingjie Hou, Li Zhang, Xiaohong Duan, Shuquan Rao, Yanhua Gong

**Affiliations:** 1 School of Disaster and Emergency Medicine, Tianjin University, Tianjin, China; 2 State Key Laboratory of Experimental Hematology, National Clinical Research Center for Blood Diseases, Haihe Laboratory of Cell Ecosystem, Institute of Hematology & Blood Diseases Hospital, Chinese Academy of Medical Sciences & Peking Union Medical College, Tianjin, China; 3 Tianjin Institutes of Health Science, Tianjin, China; 4 Department of Intensive Care Unit, Key Laboratory for Critical Care Medicine of the Ministry of Health, Emergency Medicine Research Institute, Tianjin First Center Hospital, Tianjin, China

**Keywords:** Musculoskeletal System, Urogenital System

## Abstract

Rhabdomyolysis-induced acute kidney injury (RM-AKI) is mediated primarily by myoglobin (Mb) toxicity, yet effective targeted therapies remain unavailable. Through computational structural modeling, we uncovered that the haptoglobin β-subunit (Hpβ) can bind Mb, forming a structurally stable complex. We then engineered a 55 kDa recombinant GST-Hpβ fusion protein and demonstrated that this engineered construct exhibits robust binding affinity for free Mb (17.8 kDa), thereby generating a stable 72.8 kDa GST-Hpβ-Mb complex. Significantly, this complex surpasses the molecular size threshold of the glomerular filtration barrier (~69 kDa), thereby preventing Mb from being filtered into the renal tubules and inflicting subsequent pathological damage. Further analysis revealed that the GST-Hpβ-Mb complex is expeditiously eliminated via CD163-mediated macrophage phagocytosis. Employing well-established RM-AKI murine models, we demonstrated that a single intraperitoneal administration of the GST-Hpβ fusion protein markedly improves survival, ameliorates renal function, and alleviates kidney damage, with protective effects lasting beyond a two-week period. In sum, the GST-Hpβ fusion protein offers a novel and promising biotherapeutic agent that addresses the fundamental pathophysiology of RM-AKI.

## Synopsis

An engineered haptoglobin-β (GST-Hpβ) fusion protein neutralizes toxic myoglobin (Mb) through a dual mechanism involving blockade of renal filtration and macrophage-mediated clearance, offering a novel and durable therapeutic strategy for rhabdomyolysis-induced acute kidney injury (RM-AKI).

- A recombinant GST-Hpβ fusion protein was developed that binds free Mb (17.8 kDa), forming a 72.8 kDa complex, thereby preventing glomerular filtration and accumulation in the renal tubules.
- The GST-Hpβ-Mb complex is recognized and cleared by macrophages via the endogenous CD163 scavenger receptor pathway.
- In a murine model of RM-AKI, a single intraperitoneal dose of GST-Hpβ administered immediately post-injury improved survival rates, attenuated renal dysfunction, and reduced histological kidney injury.
- The protective effect of a single administration persisted for over 14 days, exceeding the critical therapeutic window of 72 h.
- Recombinant GST-Hpβ represents a stable, cost-effective, and potentially safer therapeutic alternative to plasma-derived haptoglobin or resource-intensive interventions such as dialysis.

## Abstract (web summary)

An engineered haptoglobin-β (GST-Hpβ) fusion protein neutralizes toxic myoglobin (Mb) through a dual mechanism involving blockade of renal filtration and macrophage-mediated clearance, offering a novel and durable therapeutic strategy for rhabdomyolysis-induced acute kidney injury (RM-AKI).

## Graphical abstract

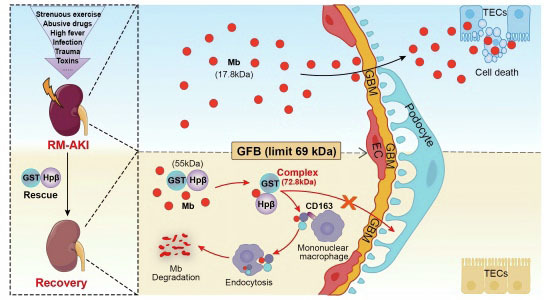

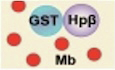

The paper explained **Problem**

Rhabdomyolysis (RM) is a severe clinical condition resulting from skeletal muscle injury, frequently leading to life-threatening acute kidney injury (AKI). Rapid breakdown of muscle tissue causes the release of large quantities of myoglobin (Mb) into the bloodstream. Due to its small molecular size, free Mb can be filtered by the glomeruli and accumulates in the renal tubules, where it forms cytotoxic casts and induces direct tubular damage. Current management of RM-AKI remains primarily supportive, such as intravenous fluid administration. No approved therapeutics that specifically target circulating Mb are available.

**Results**

This study presents a novel engineered protein therapeutic designed to neutralize circulating Mb. We identified that haptoglobin (Hp), a natural acute-phase plasma protein, binds Mb through its β-subunit (Hpβ). Leveraging this interaction, we developed a recombinant fusion protein, GST-Hpβ, which can tightly bind free Mb in circulation, forming a complex of sufficient size to prevent glomerular filtration and subsequent tubular injury. Within the bloodstream, the GST-Hpβ-Mb complex is efficiently cleared by macrophages via the CD163 scavenger receptor pathway. In a murine model of RM-AKI, a single dose of GST-Hpβ administered immediately post-injury significantly improved survival, attenuated renal dysfunction, reduced histological evidence of kidney injury, and conferred protective effects over two weeks.

**Impact**

Here, we propose GST-Hpβ as a targeted biotherapeutic strategy that directly addresses the underlying pathophysiology of RM-AKI by sequestering and promoting the clearance of the key toxic mediator, Mb. The recombinant GST-Hpβ protein offers a potentially safer, cheaper, and more efficient alternative to antibody-based approaches. This therapeutic candidate holds significant promise for the treatment of RM-AKI across diverse clinical and non-clinical contexts, including trauma, disaster response, and extreme physical exertion.

## Introduction

Rhabdomyolysis (RM) is a pathological condition that typically arises from insults to skeletal muscle tissue, such as traumatic injury, excessive physical exertion, and high fever (Bosch et al, 2009; Vanholder et al, 2000). Upon severe muscle damage, intracellular contents including the oxygen-binding heme protein myoglobin (Mb) are released into the circulation, which are capable of inducing acute kidney injury (AKI), multi-organ dysfunction, and even mortality (Huerta-Alardin et al, 2005). Alarmingly, approximately 10%-50% of RM patients progress to AKI, and the mortality rate among those afflicted with RM-AKI is approximately threefold higher than in RM patients without renal complications (Candela et al, 2020; de Meijer et al, 2003). Nevertheless, targeted therapeutic strategies to halt the progression of RM-AKI remain elusive.

Mb is a monomeric heme-containing protein with a molecular weight of 17.8 kDa, which can traverse the glomerular filtration barrier (GFB, ~69 kDa) (Slater and Mullins, 1998). Under physiological conditions, trace amounts of free Mb can enter renal tubular epithelial cells (TECs) via endocytosis and are subsequently metabolized without inducing significant damage (Elsayed and Reilly, 2010). However, in pathological states, excessive Mb floods the renal tubules, where it interacts with Tamm-Horsfall protein, accumulates, and forms obstructive tubular casts, hallmark features of RM-AKI (Zager, 1996). Accumulating evidence underscores that Mb-induced tubular obstruction constitutes a central pathogenic mechanism in the progression of RM-AKI (Boutaud et al, 2011). Furthermore, the release of iron and reactive oxygen species (ROS) from Mb directly causes oxidative damage to proximal TECs, exacerbating renal dysfunction (Luan et al, 2023; Zager, 1995). We hypothesized that strategically preventing Mb from infiltrating TECs or clearing Mb from the bloodstream could unveil a promising therapeutic avenue for the management of RM-AKI.

Haptoglobin (Hp), also known as hemopexin or bound globin, is an acute-phase plasma glycoprotein predominantly synthesized by the liver (Yueh et al, 2007). During hemolysis, Hp binds extracellular hemoglobin (Hb) through the Hp beta-subunit (Hpβ), thus forming a high-molecular-weight Hp-Hb complex that prevents Hb from extravasating through vascular endothelial junctions, including the GFB (Andersen et al, 2012; Graw et al, 2016; Wejman et al, 1984). This complex is subsequently internalized via CD163 receptor-mediated phagocytosis by monocytes and macrophages, facilitating its safe clearance from the circulation (Buehler et al, 2009; Schaer et al, 2013a). Plasma-derived Hp has been commercially available in Japan since 1985 as a therapeutic agent for the treatment of life-threatening hemolysis (Schaer et al, 2013b). Given the substantial structural and sequence homology between Mb and Hb (Fatunmbi et al, 2016; Rossifanelli et al, 1964), we hypothesized that Hp may also interact with Mb, thereby neutralizing its nephrotoxic effects.

It is worth noting that our team recently developed a high-affinity recombinant anti-Mb rabbit monoclonal antibody targeting Mb, which blocks glomerular filtration by forming large molecular complexes, and validated the feasibility of an Mb-targeted clearance strategy in an RM-AKI model (Wang et al, 2025). However, this strategy relies on the neutralizing effect of exogenous antibodies and their Fc receptor-mediated clearance pathway. In stark contrast, the present study first utilizes and extends an evolutionarily conserved endogenous detoxification system (the recombinant Hp protein), which potentially offers greater safety. Secondly, it achieves targeted clearance of pathogenic Mb proteins by directing the task to macrophages with strong phagocytic capacity via the specific receptor CD163, potentially improving clearance efficiency and reducing off-target effects. Therefore, this study is not merely a repetition of previous antibody work, but represents a fundamental mechanistic innovation—shifting from ‘exogenous blockade’ to ‘endogenous guided clearance’.

In this study, we commenced with structural modeling, which unveiled a compelling potential for interaction between Hpβ and Mb. Inspired by the concept of constructing a “molecular shield”, we engineered a 55 kDa recombinant GST-Hpβ fusion protein that exhibits robust binding affinity for free Mb (17.8 kDa), thereby generating a stable 72.8 kDa GST-Hpβ-Mb complex. Significantly, this complex can physically block free Mb from traversing the GFB, thereby halting the cascade of pathological damage. Further analysis revealed that the GST-Hpβ-Mb complex is rapidly and efficiently internalized through CD163 receptor-mediated phagocytosis by monocytes and macrophages, enabling swift systemic clearance. Employing a well-established RM-AKI murine model, we demonstrated that early administration of GST-Hpβ markedly improves survival, ameliorates renal function, and mitigates kidney injury. Significantly, a single intraperitoneal dose of the fusion protein was sufficient to confer sustained protective effects that persisted for more than two weeks. In conclusion, the recombinant GST-Hpβ fusion protein stands as a promising therapeutic strategy for RM-AKI through its dual mechanisms of Mb binding and macrophage-dependent clearance.

## Results

### Native haptoglobin binds excess myoglobin in RM-AKI mice

**Figure 1:**
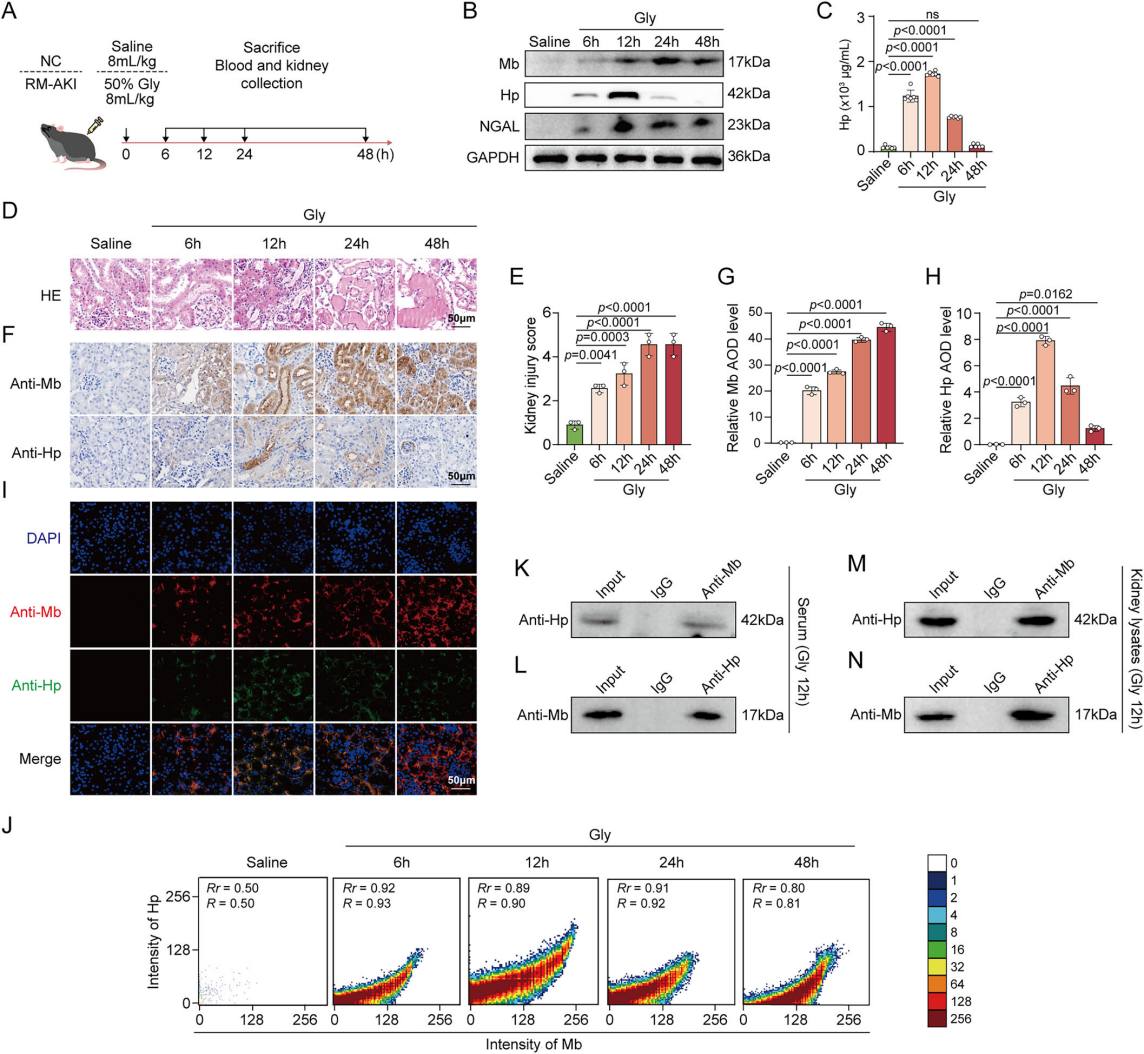
Native haptoglobin binds to excess myoglobin in RM-AKI mice. (**A**) Schematic drawing of glycerol-induced RM-AKI mice model preparation and sample collection. (**B**) WB analysis of Mb, Hp, and NGAL expression in the mouse renal cortex in the Saline and Gly groups. (**C**) ELISA analysis of the concentrations of Hp in the serum of the Saline and Gly groups (*n* = 6 per group for biological replicates). (**D**) Representative HE staining in the kidney of the Saline and Gly group mice. (**E**) Kidney injury scores (0–5) were valued and quantified according to histopathological images of kidneys in (**D**) (*n* = 3 per group for biological replicates). (**F**) Representative immunohistochemical staining of Mb and Hp in the kidney of the Saline and Gly group mice. (**G**, **H**) Quantification of Immunohistochemical data of Mb (**G**) and Hp (**H**) expression in (**F**) (*n* = 3 per group for biological replicates). (**I**) Representative confocal microscopy images of sections from kidneys harvested in Saline and Gly group mice stained for Mb (red), Hp (green), and DAPI (blue). (**J**) The fluorescence co-localization analysis of Mb and Hp via the ImageJ plugin ScatterJ. The Rr represents Pearson’s correlation, and the R represents the Overlap coefficient (0.6–1.0: have co-localized; 0–0.6: no co-localization). The closer the R-value is to 1, the more likely it is that Mb and Hp will co-localize. (**K**, **L**) Co-IP of Mb and Hp from the mouse serum of the Gly-12 h group. IP with IgG control antibody, anti-Mb antibody (**K**), or anti-Hp antibody (**L**). (**M**, **N**) Co-IP of Mb and Hp from the renal cortex of the Gly-12 h group. IP with IgG control antibody, anti-Mb antibody (**M**), and anti-Hp antibody (**N**). For statistical analysis, the one-way ANOVA (**C**, **E**, **G**, **H**) was used. Data are expressed as mean ± SD. *P* < 0.05 was considered statistically significant. ns not significant. Source data for this figure are available online.

**Figure EV1:**
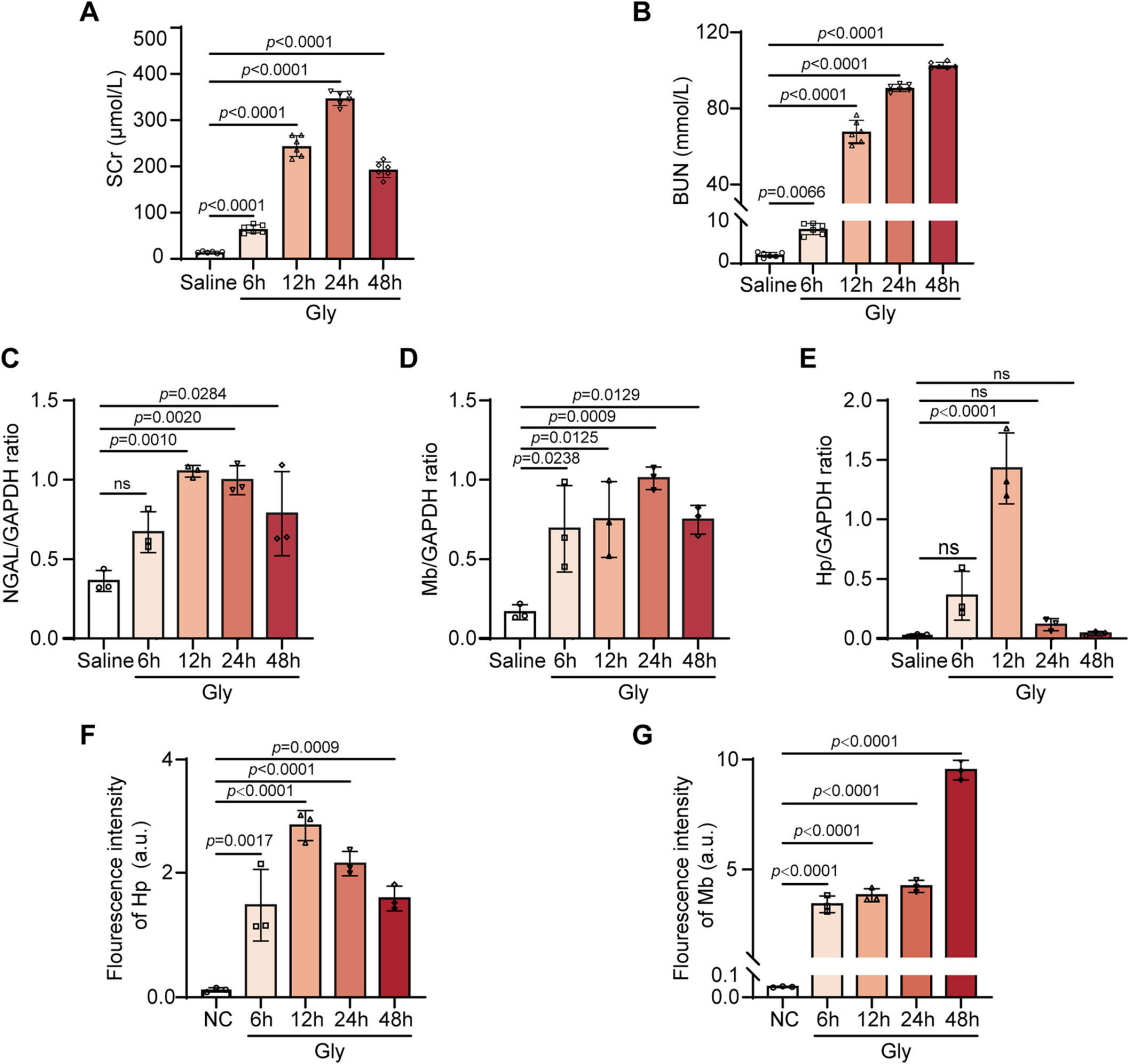
Mb and Hp expression in the kidney of RM-AKI mice. (**A**, **B**) Blood biochemical analysis of the serum concentrations of SCr (**A**) and BUN (**B**) in the glycerol-induced RM-AKI mouse model (*n* = 6 per group for biological replicates). (**C**–**E**) The grey values of the protein levels of NGAL bands (**C**), Mb bands (**D**), and Hp bands (**E**) from Western blot (Fig. 1B) were employed for statistical analysis (*n* = 3 for biological replicates). (**F**, **G**) Quantification of Immunofluorescence data of Hp (**F**) and Mb (**G**) expression in Fig. 1I (*n* = 3 per group for biological replicates). For statistical analysis, the one-way ANOVA (**A**–**G**) was used. Data are expressed as mean ± SD. *P* < 0.05 was considered statistically significant. ns not significant.

To assess the correlation between endogenous Hp and Mb, we successfully constructed the glycerol-induced non-traumatic RM-AKI mouse model (Fig. 1A), which was validated by renal function marker serum creatinine (SCr) and blood urea nitrogen (BUN) (Fig. EV1A,B), as well as the kidney injury marker neutrophil gelatinase-associated lipocalin (NGAL) (Figs. 1B and  EV1C). Meanwhile, in the HE staining assay, the renal cortex of RM-AKI mice exhibited pronounced pathological changes, including detachment of the tubular brush border, partial glomerular atrophy, and myoglobin casts (Fig. 1D,E). In the modeling group, the accumulation of Mb, a critical pathogenic factor of RM-AKI, was increased in the kidneys, forming myoglobin casts (Figs. 1B,D,F,G and  EV1D). However, Hp expression was increased in renal tissue and serum in the RM-AKI group, peaking at 12 h and then decreasing to levels similar to those of the NC (Saline) group at 48 h after modeling (Figs. 1B,C,F,H and  EV1E). These results suggested endogenous Hp was gradually exhausted during RM-AKI as the injury intensified.

Next, Mb contents in the renal tissue of the modeling group gradually increased from 6 h (Figs. 1B,D,F,G and  EV1D). In comparison, the endogenous expression level of Hp peaked at 12 h and then gradually decreased (Figs. 1B,C,F,H and  EV1E). The dynamic change in Hp levels, showing a ‘rise first and then fall’, may reflect the superimposition of two processes: ‘acute phase induction’ and ‘consumption after ligand binding’. However, this limited endogenous Hp elevation appears insufficient to counterbalance the pathological impacts of excessive Mb accumulation. Interestingly, Mb and Hp fluorescence aggregated at different time points during modeling in similar spots. Pearson correlation coefficient and overlap coefficient were nearly 1, indicating endogenous Hp and Mb co-localization in renal tissue (Figs. 1I,J and  EV1F,G). To confirm the interaction with native Hp and Mb, Co-IP assays were conducted, and results showed that native Hp bound Mb in mouse serum and kidney lysate and vice versa (Fig. 1K–N). The above evidence indicated that native Hp could bind Mb in RM-AKI mice.

### Recombinant Hpβ fusion protein binds myoglobin

**Figure 2:**
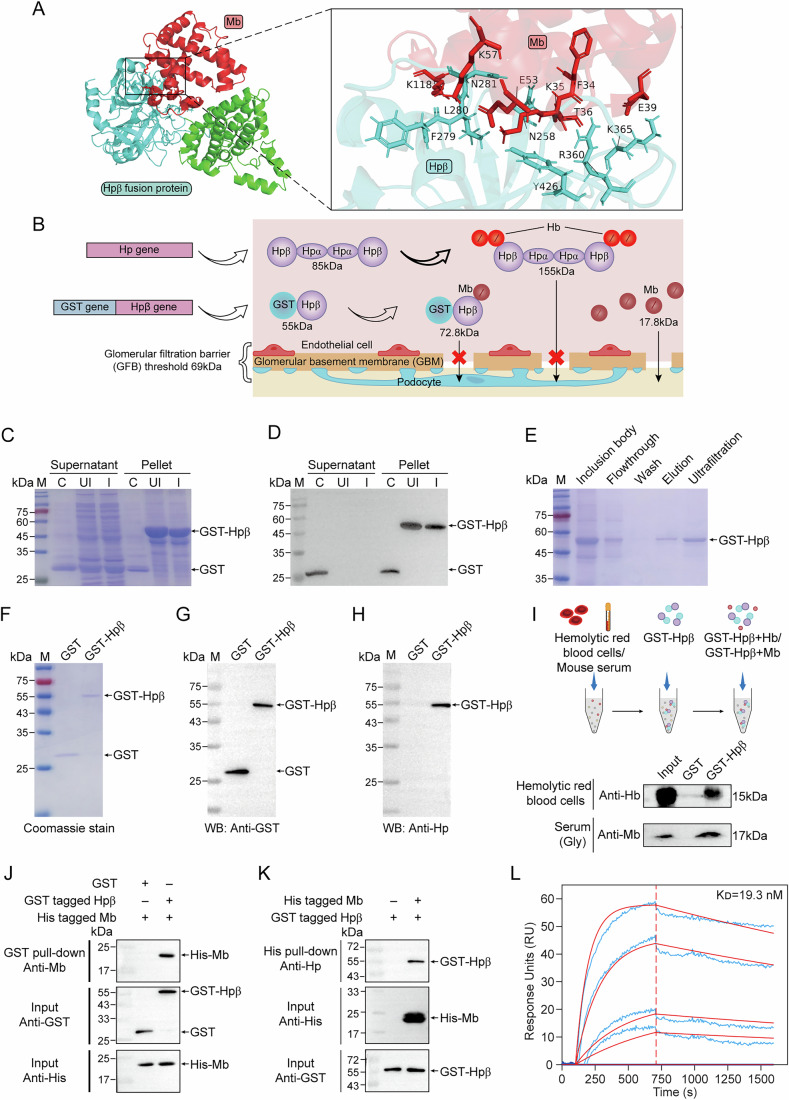
Recombinant Hpβ fusion protein binds to myoglobin. (**A**) Molecular docking of recombinant GST-Hpβ fusion protein and myoglobin (Mb) using HDOCK. PyMOL was used to visualize the binding site for the best-scoring docking poses. Specifically, the precise amino acid sites Asn23, Phe44, Leu45, Asn46, Arg125, Lys130 and Tyr191of Hpβ alone (that is, Asn258, Phe279, Leu280, Asn281 Arg360, Lys365 and Tyr426 of GST-Hpβ) interact with the amino acid sites Phe34, Lys35, Thr36, Glu39, Glu53, Lys57 and Lys118 of Mb. (**B**) Schematic drawing of the concept of physically blocking the passage of free Mb through the glomerular filtration barrier (GFB). Tetrameric Hp protein (85 kDa) binds to two Hb proteins, forming a high-molecular-weight Hp-Hb complex (155 kDa), exceeding the glomerular filtration barrier (GFB, ~ 69 kDa). GST-Hpβ fusion protein (55 kDa) binds at least one Mb, forming a GST-Hpβ-Mb complex (72.8 kDa) over the size of GFB (~ 69 kDa). (**C**, **D**) Coomassie blue staining (**C**) and WB analysis with anti-GST antibody (**D**) for GST-HPβ fusion protein expression and solubility. M-Marker, C-Control of vector strain, UI-Uninduced plasmid-containing strain, I-Induced plasmid-containing strain. (**E**) Coomassie blue staining shows the GST-Hpβ fusion protein purification process. (**F**–**H**) Coomassie blue staining (**F**), WB analysis with anti-GST antibody (**G**) and anti-Hp antibody (**H**). (**I**) GST pulldown assays analysis of the interaction between GST or GST-Hpβ fusion protein and Hb from the hemolytic red blood cells of mice or Mb from the serum of glycerol-induced RM-AKI mice. (**J**) GST pulldown assays analyze the interaction between GST or GST-Hpβ fusion protein and recombinant mouse His-Mb. (**K**) His pulldown assays analyze the interaction between His-Mb and GST-Hpβ fusion protein. (**L**) SPR sensorgrams of GST-Hpβ to the recombinant mouse His-Mb immobilized sensor chip. The raw data is shown in blue lines, and the calculated fit is shown in red. The running buffer used for ligand attachment and analyte-binding experiments was 0.01 M PBS containing 2 mM KH_2_PO_4_, 8 mM Na_2_HPO_4_·12H_2_O, 136 mM NaCl, and 2.6 mM KCl, adjusted to pH 7.4. Source data for this figure are available online.

**Figure EV2:**
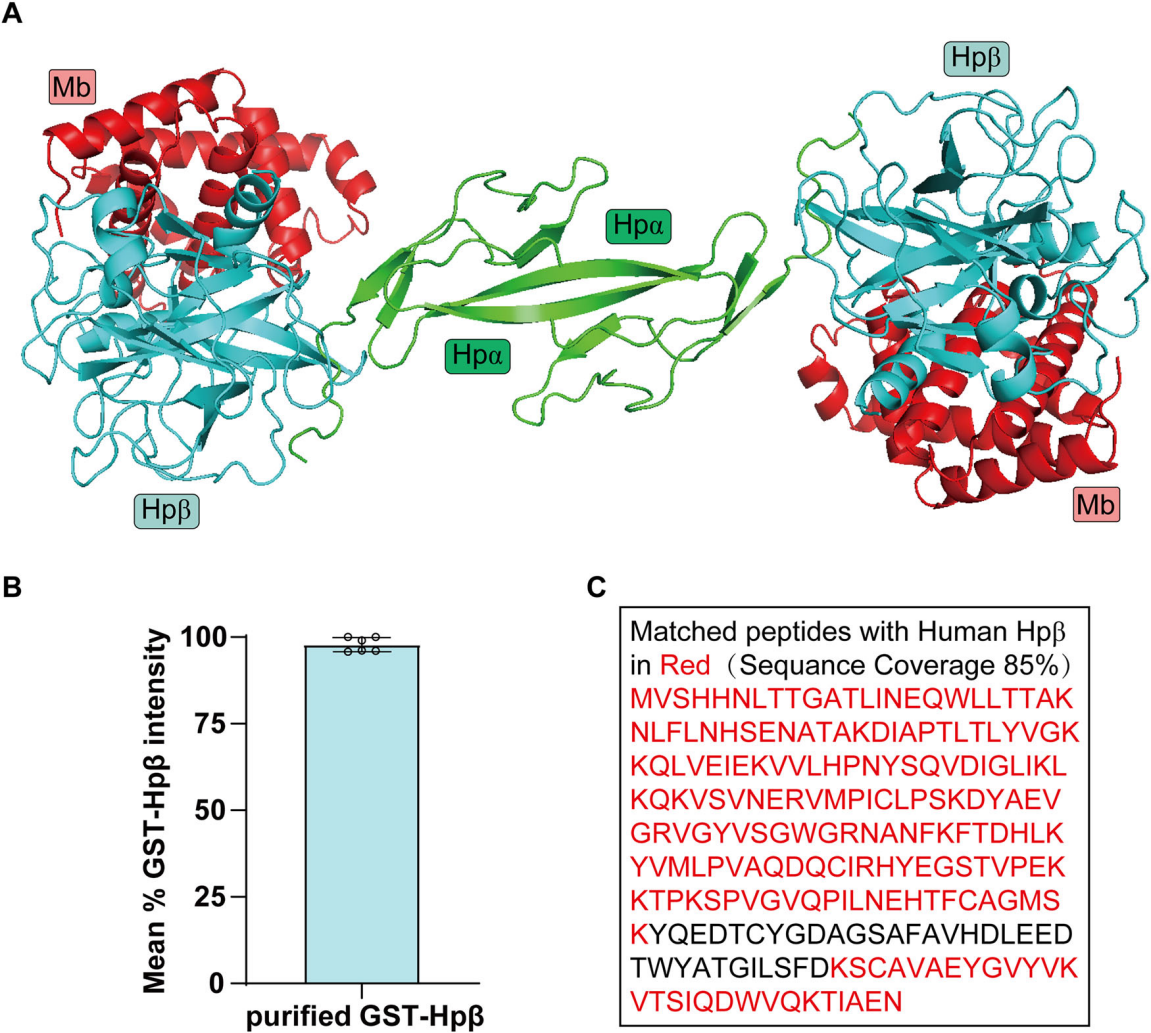
Mb binding region focuses on Hp beta-subunit (Hpβ) predicted by molecular docking and the identification of recombinant GST-Hpβ fusion protein. (**A**) Molecular docking of the tetramers Hp protein and Mb using HDOCK. PyMOL was used to visualize the binding site for the best-scoring docking poses. (**B**) Quantitative analysis of gray values of the GST-Hpβ fusion protein in Fig. 2E,F by ImageQuant™ TL analysis software. The purity of recombinant GST-Hpβ fusion protein was greater than 95% (*n* = 6 for biological replicates). Data are expressed as mean ± SD. (**C**) Protein mass spectrometry analysis for purified GST-Hpβ fusion protein.

To predict the binding region of Hp and Mb, we perform HDOCK molecular docking. Results showed that the Mb binding region was concentrated in Hp beta-subunits (Hpβ) (Figs. 2A and  EV2A). These results suggest that the interaction between Hpβ and Mb has a specific structural basis. Based on the concept of creating a “molecular shield” in the blood circulation, the therapeutic protein needs to bind to extracellular free Mb (17.8 kDa), forming a macromolecular complex, which will be physically incapable of passing through the primary membrane aperture of the GFB (~69 kDa), thus blocking the renal toxicity of Mb. Therefore, we designed a GST-Hpβ fusion protein with a molecular weight of 55 kDa (Fig. 2B). The GST-Hpβ-Mb complexes (72.8 kDa) exceed the molecular size threshold (~69 kDa) of the glomerular filtration barrier.

The prokaryotic expression system was established for inducible expression of GST-Hpβ, and Coomassie blue staining and WB showed that high amounts of GST-Hpβ were successfully expressed in inclusion bodies (Fig. 2C,D). The GST-Hpβ fusion protein was purified by affinity chromatography to give a final purity greater than 95% (Figs. 2E,F and  EV2B). Moreover, we used WB analysis with anti-GST or anti-Hp specific antibodies to confirm that the purified fusion protein was indeed GST-Hpβ (Fig. 2G,H). Furthermore, protein mass spectrometry analysis confirmed the correctness of the Hpβ sequence (Fig. EV2C).

GST pulldown assays showed that GST-Hpβ fusion protein could bind to endogenous Hb protein from hemolytic red blood cells, which showed that the purified GST-Hpβ protein was active (Fig. 2I). To investigate whether GST-Hpβ fusion protein could bind native or artificial Mb, we first performed a GST pulldown assay, which demonstrated the interaction of GST-Hpβ with natural Mb in the serum of glycerol-induced non-traumatic RM-AKI mice (Fig. 2I). Next, GST and His pulldown assays were used to detect the direct interaction between GST-Hpβ fusion protein and recombinant mouse His-Mb in vitro. Results showed that the GST-Hpβ could bind explicitly to the recombinant mouse His-Mb and vice versa (Fig. 2J,K). Moreover, the binding affinities and kinetic properties of GST-Hpβ to His-Mb were assessed using surface plasmon resonance (SPR). The equilibrium dissociation constant (K_D_) value is 19.3 nM, indicating that the GST-Hpβ fusion protein has a strong binding affinity with mouse His-Mb (Fig. 2L). These results demonstrated that the purified recombinant GST-Hpβ fusion protein exhibited good biological activity and could specifically bind to Mb both in vivo and in vitro.

### Early recombinant Hpβ fusion protein injection alleviates RM-AKI in mice

**Figure 3:**
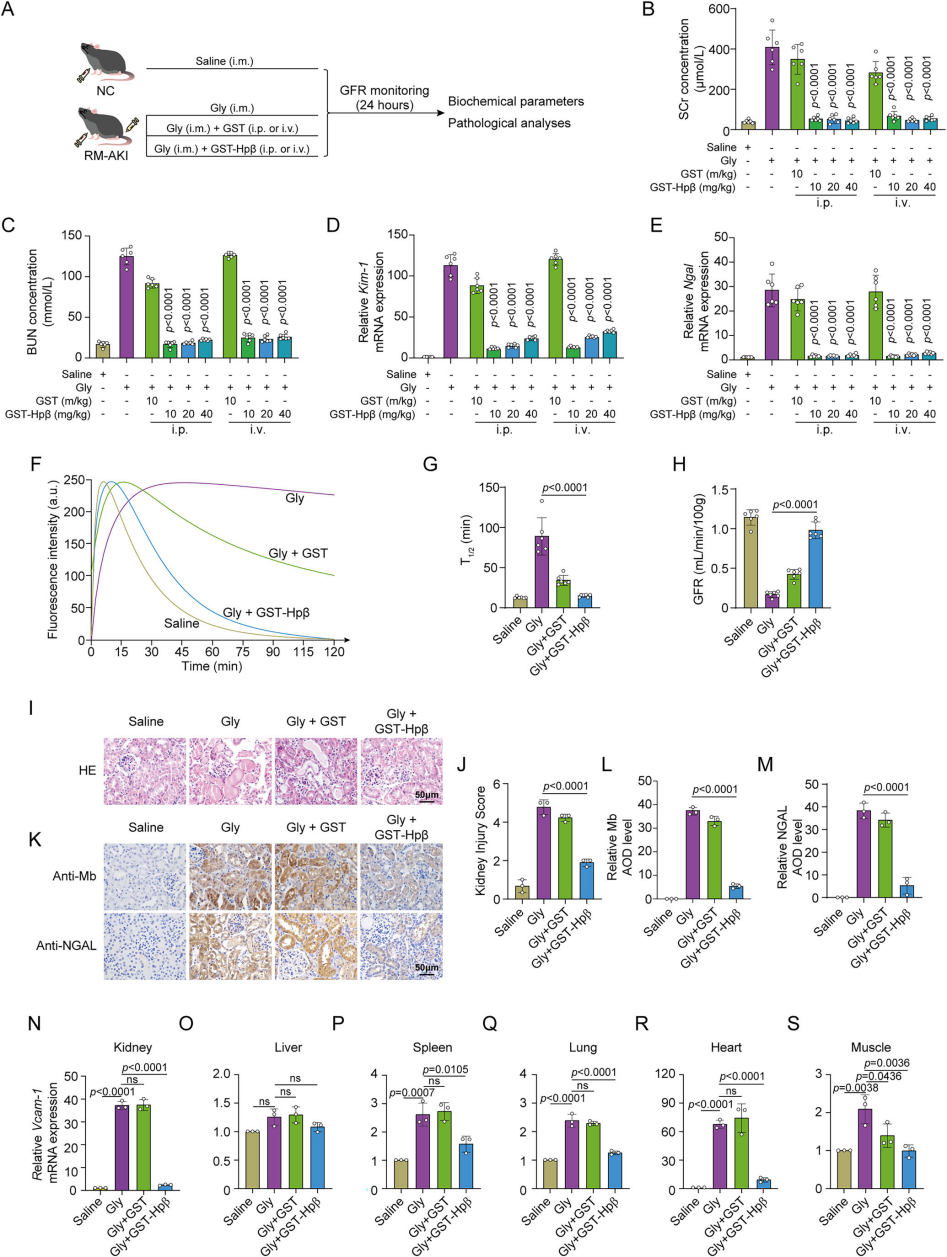
Recombinant Hpβ fusion protein injection alleviates RM-AKI in vivo*.* (**A**) Experimental design of GST-Hpβ fusion protein treatment for glycerol-induced RM-AKI mice. Intramuscular glycerol injection (Gly i.m.) to prepare glycerol-induced RM-AKI model mice. Meanwhile, GST-Hpβ or GST is injected intravenously (i.v.) or intraperitoneally (i.p.), respectively, to simulate early-stage clinical intervention. After 24 h, renal function is measured by GFR. Blood and tissue samples are collected for biochemical testing and pathological analysis. (**B**, **C**) Blood biochemical analysis of the concentrations of SCr (**B**) and BUN (**C**) in serum in each group of mice. All *P* values are compared with the Gly group (*n* = 6 per group for biological replicates). (**D**, **E**) qPCR analysis of *Kim-1* (**D**) and *Ngal* (**E**) mRNA expression in the renal cortex in each group of mice. All *P* values are compared with the Gly group (*n* = 6 per group for biological replicates). (**F**) Representative image of transcutaneous disappearance curves of FITC-sinistrin excretion after 24 h in i.p. injection with 10 mg/kg GST-Hpβ group. (**G**) The excretion half-life (T_1/2_) of FlTC-sinistrin in the selected groups of mice (*n* = 6 per group for biological replicates). (**H**) GFR in the groups of mice was measured at 24 h (*n* = 6 per group for biological replicates). (**I**) HE staining analyzes renal tubular injury degree in the Gly+GST-Hpβ 10 mg/kg group by the i.p. method. (**J**) Blinded kidney injury score of the selected groups of mice (*n* = 3 per group for biological replicates). (**K**) Representative IHC staining images of Mb and NGAL expression in kidney tissues. (**L**, **M**) Quantification of the integrated optical density (IOD) of Mb (**L**) and NGAL (**M**) in the image of (**K**) (*n* = 3 per group for biological replicates). (**N**–**S**) Endothelial activation marker *Vcam-1* mRNA expression in the affected organs (kidney, (**N**), muscle (**S**)) and other organs (liver (**O**), spleen (**P**), lung (**Q**), and heart (**R**)) of the selected groups of mice at 24 h (*n* = 3 per group for biological replicates). For statistical analysis, the one-way ANOVA (**B**–**E**, **G**, **H**, **J**, **L**–**S**) was used. Data are expressed as mean ± SD. *P* < 0.05 was considered statistically significant. ns not significant. Source data for this figure are available online.

To verify the early-stage therapeutic effect of the GST-Hpβ fusion protein in glycerol-induced non-traumatic RM-AKI mice (Fig. 3A), we first need to explore the optimal administration mode and dosage after modeling. Therefore, two administration routes (intraperitoneal (i.p.) and intravenous (i.v.)) and three dosages (10, 20, and 40 mg/kg) of the GST-Hpβ fusion protein were used. GST-Hpβ treatment significantly decreased the kidney function biomarker SCr and BUN levels in the blood of RM-AKI mice compared with the Gly group (Fig. 3B,C). Meanwhile, the mRNA expression levels of renal tubular injury markers kidney injury molecule-1(*Kim-1)* and *Ngal* in the Gly+GST-Hpβ group were markedly reduced compared with those in the Gly group (Fig. 3D,E). Moreover, the therapeutic effect was comparable between i.p. and i.v. routes, and the 10 mg/kg administration was found to be the most appropriate dose (Fig. 3B–E). Therefore, the optimal administration strategy of the GST-Hpβ fusion protein was the immediate intraperitoneal (i.p.) injection of 10 mg/kg upon RM-AKI.

**Figure EV3:**
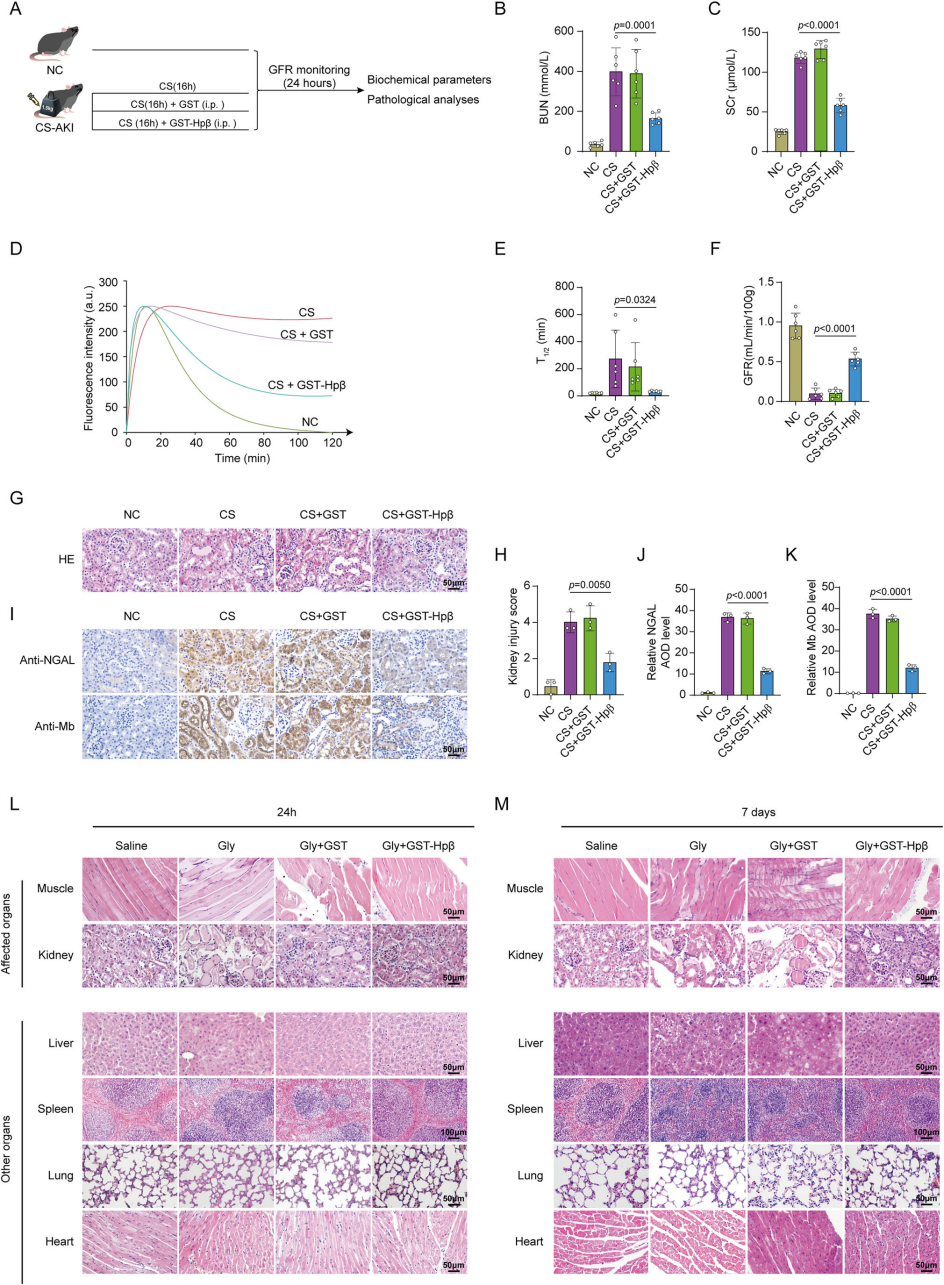
Therapeutic effects of GST-Hpβ on CS-AKI mice and pathological examination of major organs in glycerol-induced RM-AKI mice. (**A**) Experimental design of GST-Hpβ fusion protein treatment for CS-AKI mice. Mice were subjected to 1.5 kg of compression for 16 h, and GST-Hpβ was injected immediately after decompression. Kidney function in the mice was tested 24 h later, blood samples were collected for biochemical examination, and tissue samples were collected for pathological analysis. (**B**, **C**) Blood biochemical analysis of the concentrations of BUN (**B**) and SCr (**C**) in serum in NC and CS-AKI mice (*n* = 6 per group for biological replicates). (**D**) Representative image of transcutaneous disappearance curves of Inulin-FITC excretion after 24 h in i.p. injection with 10 mg/kg GST-Hpβ of CS-AKI mice. (**E**) The excretion half-life (T_1/2_) of Inulin-FITC in NC, CS, CS + GST, and CS + GST-Hpβ groups of mice (*n* = 6 per group for biological replicates). (**F**) GFR of mice in NC, CS, CS + GST, and CS + GST-Hpβ groups was measured at 24 h (*n* = 6 per group for biological replicates). (**G**) HE staining assesses renal tubular injury severity in selected NC, CS, CS + GST, and CS + GST-Hpβ groups. (**H**) Blinded kidney injury score of the selected groups of mice in the image of (**G**) (*n* = 3 per group for biological replicates). (**I**) Representative IHC staining images of Mb and NGAL expression in kidney tissues of NC, CS, CS + GST, and CS + GST-Hpβ groups. (**J**, **K**) Quantification of the integrated optical density (IOD) of NGAL (**J**) and Mb (**K**) in the image of (**I**) (*n* = 3 per group for biological replicates). (**L**, **M**) HE staining was used to analyze the injury in the affected organs (muscle, kidney) and other organs (liver, spleen, lung, and heart) in glycerol-induced RM-AKI model mice after injection of recombinant GST-Hpβ fusion protein for 24 h or 7 d. For statistical analysis, the one-way ANOVA was used (**B**, **C**, **E**, **F**, **H**, **J**, **K**). Data are expressed as mean ± SD. *P* < 0.05 was considered statistically significant. ns not significant.

To further confirm the therapeutic effect of the optimal administration strategy for GST-Hpβ, we used glomerular filtration rate (GFR), the gold standard in clinical nephrology, to assess renal function. The results indicated a rapid decline in the GFR of glycerol-induced non-traumatic RM-AKI mice in 24 h after modeling, while the GFR of mice in the GST-Hpβ treatment group significantly improved (Fig. 3F–H). Moreover, histopathological results showed that all mice in the Gly and Gly+GST groups exhibited varying degrees of kidney damage, including renal tubular dilation, cast formation, intraluminal cell death and shedding, and brush border disappearance (Fig. 3I,J). However, GST-Hpβ treatment significantly alleviated renal damage (Fig. 3I,J). In addition, Mb accumulation and kidney tubular injury marker NGAL expression were significantly reduced in the renal tissue of the Gly+GST-Hpβ group as opposed to the Gly group (Fig. 3K–M). Furthermore, we validated the therapeutic efficacy of GST-Hpβ in a mouse model of traumatic rhabdomyolysis-associated acute kidney injury (crush syndrome-associated AKI, CS-AKI) (Fig. EV3A). Compared with the normal control group (NC group), mice in the CS-AKI model group (CS group) exhibited significantly elevated serum levels of BUN and SCr at 24 h after decompression (Fig. EV3B, C), along with a marked reduction in GFR (Fig. EV3D–F). Histopathological examination revealed typical renal injuries, including cast formation, brush border detachment, and glomerular shrinkage (Fig. EV3G,H), collectively indicating successful establishment of the CS-AKI mouse model. Immediately after decompression, CS-AKI mice received an intraperitoneal injection of 10 mg/kg GST-Hpβ fusion protein (CS + GST-Hpβ group). Compared with the CS group, the CS + GST-Hpβ group showed significantly decreased serum BUN and SCr levels (Fig. EV3B,C) and a marked recovery of GFR (Fig. EV3D–F), indicating improved renal function. Moreover, both the renal injury score and the expression level of the kidney tubular injury marker NGAL were significantly reduced in the CS + GST-Hpβ group (Fig. EV3G–J). Notably, Mb accumulation in the renal tubules was also markedly attenuated (Fig. EV3I,K). Collectively, these results demonstrate that GST-Hpβ treatment significantly ameliorates renal dysfunction and reduces kidney injury in glycerol-induced non-traumatic RM-AKI mice or CS-AKI mice.

Meanwhile, qPCR results showed that endothelial activation marker *Vcam-1* mRNA expression in the Gly+GST-Hpβ group was reduced in both affected organs (kidney, muscle) and other organs (spleen, lung, heart) compared with the Gly and Gly+GST group (Fig. 3N–S). The GST-Hpβ-Mb complexes do not cause systemic endothelial toxicity. Moreover, compared to the NC (Saline) group, the other three groups showed muscle damage, demonstrating the successful construction of the RM model (Fig. EV3L,M). Compared with the Gly group, the Gly+GST-Hpβ group showed reduced pathological damage in the affected organ kidney, and no significant changes were observed in the liver, spleen, heart, and lung at 24 h or 7 d (Fig. EV3L,M). These results indicated that early-stage GST-Hpβ fusion protein treatment improves kidney function, reduces kidney injury, and does not cause apparent toxic adverse effects on vital organs within 7 days of injection.

### Recombinant Hpβ fusion protein prevents free Mb from entering TECs and guides Mb to macrophages

**Figure 4:**
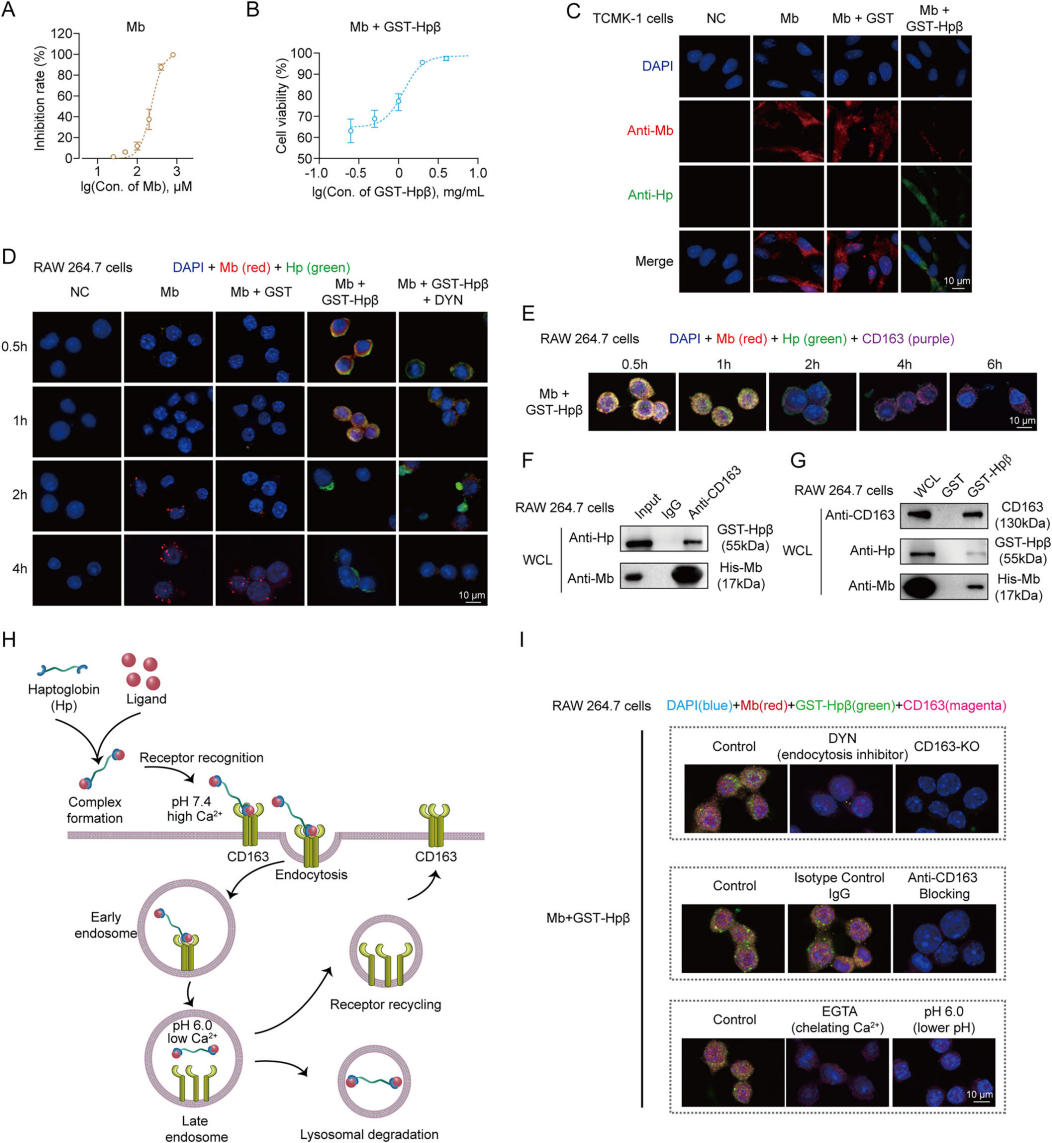
Recombinant Hpβ fusion protein prevents free Mb from entering TECs and guides Mb to macrophages. (**A**) CCK-8 detects the inhibition rate of TCMK-1 cells treated with different concentrations (25 μM, 50 μM, 100 μM, 200 μM, 400 μM, 800 μM) of mouse ferrous His-Mb, and calculates the IC_50_ value (*n* = 3 per group for biological replicates). Data are expressed as mean ± SD. (**B**) CCK-8 detects the survival rate of TCMK-1 cells treated with 200 μM mouse ferrous His-Mb and different concentrations (0.25 mg/mL, 0.5 mg/mL, 1 mg/mL, 2 mg/mL, 4 mg/mL, and 8 mg/mL) of GST-Hpβ fusion protein (*n* = 3 per group for biological replicates). Data are expressed as mean ± SD. (**C**) Representative confocal microscopy images of TCMK-1 cells treated with 200 μM ferrous His-Mb (red) or together with 2 mg/mL GST-Hpβ fusion protein (green) or 2 mg/mL GST for 6 h. (**D**) Representative confocal microscopy images of RAW 264.7 cells treated with ferrous His-Mb (red), GST-Hpβ fusion protein (green), and macrophage membrane dynamic protein inhibitor DYN for 4 h. (**E**) Representative confocal microscopy images of RAW 264.7 cells treated with ferrous His-Mb (red), GST-Hpβ fusion protein (green) for different times (0.5 h, 1 h, 2 h, 4 h, and 6 h) stained for CD163 (purple). (**F**) RAW 264.7 cells treated with 20 ng/mL IL-4 for 48 h to increase CD163 expression, then incubated with GST-Hpβ-Mb protein complex (GST-Hpβ, 100 µg/mL; His-Mb, 37.5 µg/mL; molar ratio= 1:1) for 0.5 h. RAW 264.7 cell lysate was incubated with anti-CD163 antibody-Protein A magnetic beads. Represented Co-IP results of GST-Hpβ, His-Mb of the above treatment. IP with IgG control antibody (Solarbio, #SP031), anti-CD163 antibody (Santa Cruz, #sc-58965), anti-Mb antibody (Abcam, #ab77232), or anti-Hp antibody (HUABIO, #ET1703-24). WCL, whole cell lysate. (**G**) GST pulldown assays analysis of the interaction between GST-Hpβ-Mb and CD163 from IL-4-treated RAW 264.7 cells. WCL, whole cell lysate. (**H**) Mechanism of CD163-mediated endocytosis of Hp complex. On the surface of macrophages, CD163 forms calcium- and pH-dependent oligomers that bind to the Hp-ligand complex. After phagocytosis, the low Ca^2+^ concentration and low pH in late lysosomes cause CD163 to dissociate, releasing the Hp-ligand complex. Monomeric CD163 returns to the cell membrane, while the Hp-ligand complex is degraded in the lysosome. (**I**) Representative confocal microscopy images of RAW 264.7 cells treated with GST-Hpβ-Mb protein complex (37.5 µg ferrous His-Mb (red), 100 µg/mL GST-Hpβ fusion protein (green)) in the presence or absence of human IgG1 isotype control (Selleck, Cat# A2501, 10 μg/mL), anti-CD163 antibody (Selleck, Cat# A2565, 10 μg/mL), EGTA (2 mM), pH 6.0, or macrophage membrane dynamic protein inhibitor DYN (80 μM) for 0.5 h stained for CD163 (purple). The CRISPR/Cas9 technique was used to generate *Cd163*-knockout (KO) RAW264.7 cell lines. Source data for this figure are available online.

**Figure EV4:**
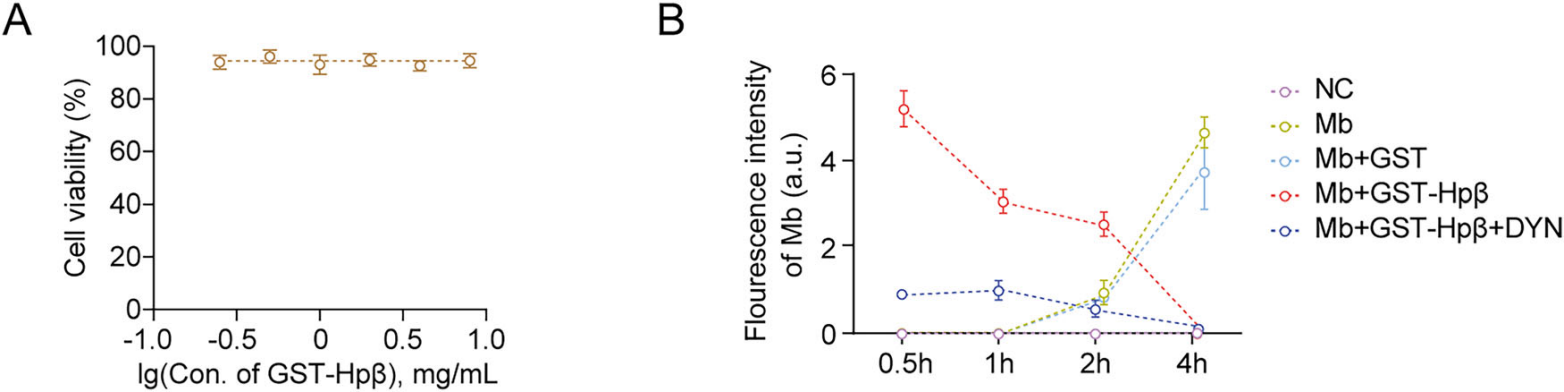
Toxicity of GST-Hpβ to TCMK-1 cells and quantification of confocal microscopy images of RAW 264.7 cells metabolism of Mb. (**A**) CCK-8 detects the survival rate of TCMK-1 cells treated with different concentrations (0.25 mg/mL, 0.5 mg/mL, 1 mg/mL, 2 mg/mL, 4 mg/mL, and 8 mg/mL) of GST-Hpβ fusion protein. (**B**) Quantification of confocal microscopy images of RAW 264.7 cells metabolism of Mb in Fig. 4D. Quantitative analysis of the fluorescence intensity of mouse His-Mb proteins in confocal microscopy images of RAW 264.7 cells for different times (0.5, 1, 2, 4 h). Results are presented as mean ± SD, and dots indicate individual quantitative analysis data points from three biological replicates.

To investigate the mechanism by which the GST-Hpβ fusion protein alleviates kidney injury, following the pathophysiology of RM-AKI, the immortalized mouse renal tubular epithelial cell line TCMK-1 was exposed to Mb to simulate an in vitro RM-AKI cellular model. The purified recombinant mouse His-Mb had dose-dependent cytotoxic effects on TCMK-1 cells, and the IC_50_ value amounted to 226.7 μM (Fig. 4A). Concerning earlier experience (Wang et al, 2021b), a 200 μM concentration of His-Mb, which is below the IC_50_ threshold, was selected for further cellular assays. Meanwhile, CCK-8 assay results showed that the GST-Hpβ fusion protein exhibited negligible cytotoxicity in TCMK-1 cells, even at 8 mg/mL (Fig. EV4). Moreover, TCMK-1 cells were exposed to 200 μM His-Mb and a range of GST-Hpβ concentrations (0.25-8 mg/mL). The GST-Hpβ dose-dependently reduced the damage of His-Mb to TCMK-1 cells and displayed an EC_50_ value of 1.154 mg/mL against 200 μM His-Mb toxicity of TCMK-1 cells (Fig. 4B). Therefore, a 200 μM dose of His-Mb and a 2 mg/mL concentration of GST-Hpβ were chosen for follow-up in vitro TCMK-1 cell studies.

Then, TCMK-1 cells were incubated with 200 μM His-Mb or together with 2 mg/mL GST-Hpβ fusion protein for 6 h. Findings indicated that the presence of Mb along with GST-Hpβ in the treatment led to a decrease in Mb accumulation in TCMK-1 cells compared with the group treated with Mb alone (Fig. 4C). These findings suggest that GST-Hpβ can effectively prevent Mb accumulation in TCMK-1 cells in vitro. We wonder where the GST-Hpβ-Mb protein complex goes. Do phagocytes engulf it? To test our hypothesis, RAW 264.7 cells were incubated with 37.5 µg His-Mb or together with 100 µg/mL GST-Hpβ fusion protein for 0.5 h, 1 h, 2 h, and 4 h, respectively. Immunofluorescence results showed that RAW 264.7 cells incubated with Mb and GST-Hpβ fusion protein together could accelerate phagocytosis and degradation of Mb (Figs. 4D and  EV4B). DYN, an inhibitor known to block phagocytosis, was added to RAW 264.7 cells and inhibited the uptake of GST-Hpβ-Mb protein complex in macrophages (Figs. 4D and  EV4B). In addition, CD163 on macrophages is a high-affinity natural receptor for the Hp-Hb complex. Does the recognition of the GST-Hpβ-Mb protein complex by macrophages also occur through the CD163 receptor? Immunofluorescence results showed that the GST-Hpβ-Mb protein complex co-localized with CD163 on the surface of RAW 264.7 cells (Fig. 4E). Co-IP or GST-pulldown results indicated the interaction between CD163 and the GST-Hpβ-Mb protein complex (Fig. 4F,G). Moreover, knocking out CD163 in macrophages or blocking its function with an anti-CD163 antibody was shown to interfere with CD163 binding to the GST-Hpβ-Mb protein complex (Fig. 4H,I). Since the CD163 receptor is calcium- and pH-dependent, chelating Ca^2+^ with EGTA or lowering the pH to 6.0 can both disrupt the binding of CD163 to the GST-Hpβ-Mb protein complex (Fig. 4H,I). These results suggested that the surface receptor CD163 on RAW 264.7 cells can recognize the GST-Hpβ fusion protein and perform receptor-mediated phagocytosis of Mb.

### Recombinant Hpβ fusion protein improves RM-AKI mice outcomes within 14 days

**Figure 5:**
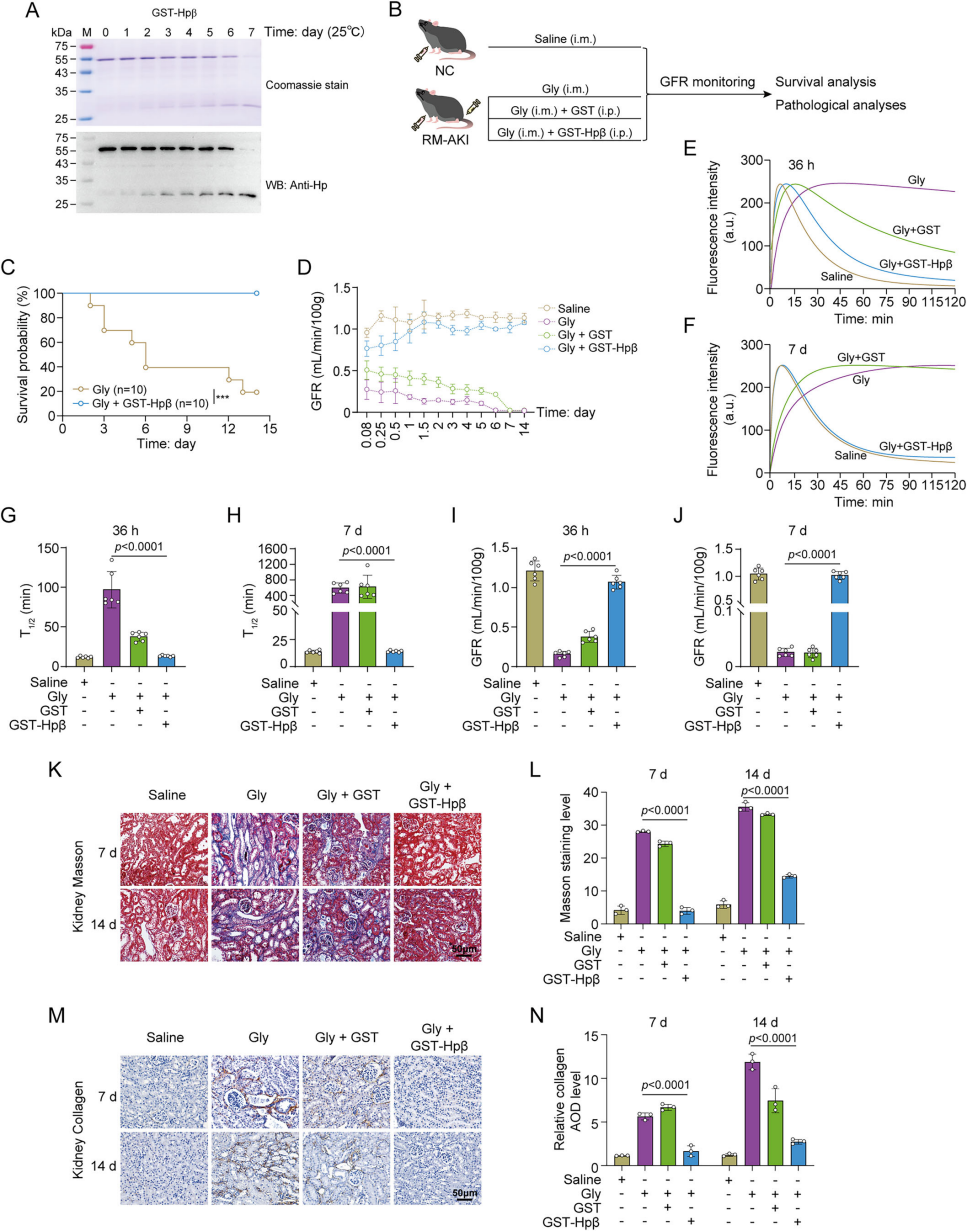
Recombinant Hpβ fusion protein improves RM-AKI outcomes within 14 days. (**A**) Stability analysis of GST-Hpβ fusion protein at 25 °C with Coomassie blue staining and WB. (**B**) Experimental design schematic of GST-Hpβ fusion protein treatment for glycerol-induced RM-AKI mice. (**C**) Survival rate curves of mice in the Gly and Gly+GST-Hpβ group within 14 days. The log-rank test was used to compare survival curves. (*n* = 10 per group for biological replicates). (**D**) GFR of each group of mice within 14 days (*n* = 3 per group for biological replicates). (**E**, **F**) Representative image of transcutaneous disappearance curves of FITC-sinistrin excretion. (**G**, **H**) The excretion half-life (T_1/2_) of FlTC-sinistrin was calculated at 36 h and 7 d in each group of mice (*n* = 6 per group for biological replicates). (**I**, **J**) GFR value at 36 h and 7 d in each group of mice (*n* = 6 per group for biological replicates). (**K**) Masson staining of kidney sections showed the distribution of collagen (blue) in tissues. (**L**) Quantitative analysis of the level of Masson staining of (**K**) (*n* = 3 per group for biological replicates). (**M**) Collagen III IHC staining of kidney sections showed deposition of Collagen III in the tissues. (**N**) Quantitative analysis of the relative Integrated Optical Density (IOD) level of Collagen III in the kidney (*n* = 3 per group for biological replicates). For statistical analysis, the one-way ANOVA (**G**–**J**, **L**, **N**) was used. Data are expressed as mean ± SD. All *P* values were compared with the Gly group, and *P* < 0.05 was considered statistically significant. Source data for this figure are available online.

Protein stability is the basis for whether it has a stable therapeutic effect and can be widely used in disaster areas or clinics. To investigate the stability of the purified GST-Hpβ fusion protein, it was placed at 25 °C for 7 days. Coomassie blue staining and WB results showed that GST-Hpβ was stable within 3 days (Fig. 5A). Obvious fracture and degradation occurred on the sixth day (Fig. 5A). Therefore, we are curious to see how long the therapeutic effect of GST-Hpβ lasts in vivo (Fig. 5B). Results showed that one-dose i.p. administration of l0 mg/kg GST-Hpβ fusion protein immediately after modeling could significantly improve the 14-day survival rate of glycerol-induced non-traumatic RM-AKI mice (Fig. 5C). Meanwhile, renal function GFR value of mice in the Gly group gradually decreased. Renal function was almost zero at day 7, while the GFR in the GST-Hpβ treatment group gradually increased, recovered to the level of the NC (Saline) group at 36 h, and could be maintained for 14 days (Fig. 5D–J). In addition, Masson staining and collagen III IHC staining showed that mice in the Gly and Gly+GST groups exhibited varying degrees of renal fibrosis (Fig. 5K–N). In contrast, no prominent fibrosis was observed in the GST-Hpβ treatment group on the 14th day (Fig. 5K–N). These results indicated that a single dose of recombinant GST-Hpβ fusion protein administration exhibits enduring treatment efficacy within two weeks in RM-AKI mice and mitigates renal fibrosis.

### Metabolic imaging of Hpβ fusion protein in RM-AKI mice

**Figure 6:**
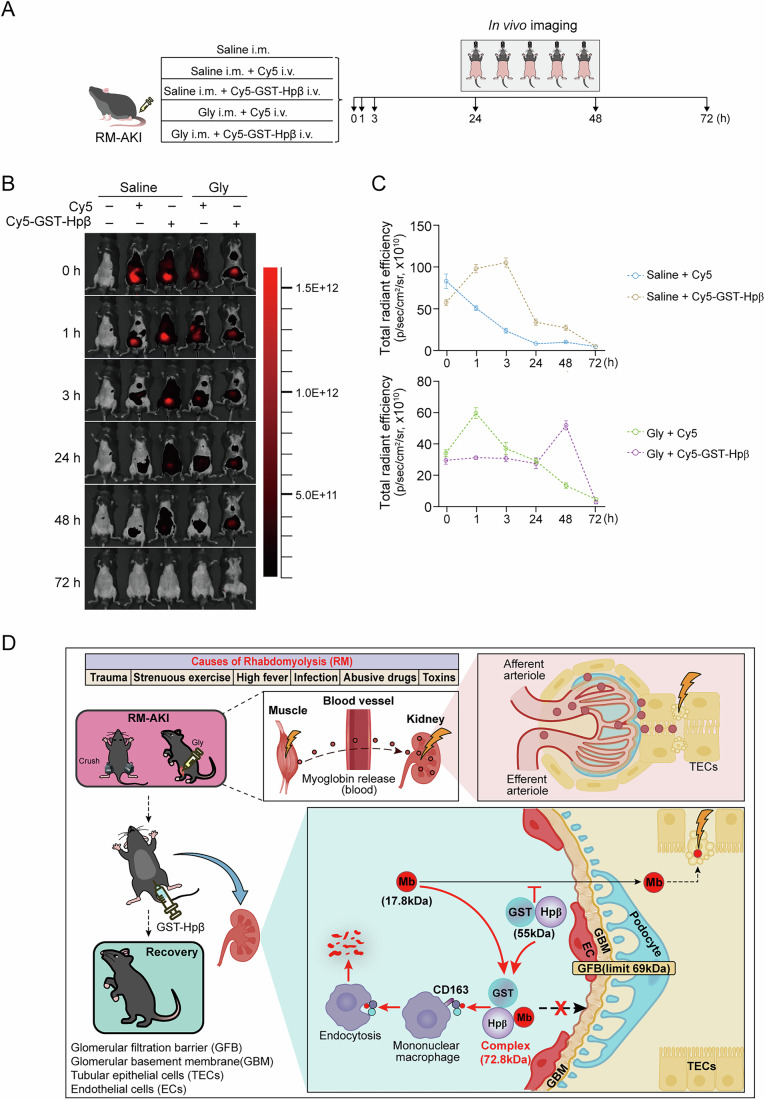
Metabolic imaging of Hpβ fusion protein in RM-AKI mice. (**A**) Experimental design schematic of in vivo imaging for glycerol-induced RM-AKI mice. (**B**) In vivo imaging of each group of mice within 72 h. (**C**) The fluorescence intensity of each group of mice at different time points in the image of (B) (*n* = 3 per group for biological replicates). Data are expressed as mean ± SD. (**D**) Schematic diagram of this research. GST-Hpβ fusion protein (55 kDa) binds free Mb (17.8 kDa), forming a large molecular weight complex over the glomerular filtration barrier (GFB, ~69 kDa), and reducing the accumulation of Mb in the kidney. The GST-Hpβ-Mb complex was degraded via CD163 receptor-mediated phagocytosis of macrophages, finally ameliorating RM-AKI. Source data for this figure are available online.

Since the therapeutic effect of the GST-Hpβ fusion protein can be maintained for 14 days in the RM-AKI model, how long will GST-Hpβ be metabolized and eliminated? To answer this question, the purified GST-Hpβ fusion protein was labeled with cyanine dye Cy5 (Cy5-GST-Hpβ). The preliminary tissue distribution and metabolic fate of Cy5-GST-Hpβ were monitored in vivo for up to 72 h following intravenous injection (Fig. 6A,B). Results showed that GST-Hpβ in mice of the Saline group and Gly group had been completely cleared within 72 h, and the initial time point of GST-Hpβ metabolism in Gly group mice was significantly delayed at least 3 h (Fig. 6C).

## Discussion

Rhabdomyolysis is a significant cause of AKI due to the accumulation of Mb in the kidney. The mortality rate of RM-AKI patients was reported to be as high as 60% (Li et al, 2022). However, at present, RM-AKI is mainly treated symptomatically, and there are no FDA-approved specific drugs. It is noteworthy that rhabdomyolysis stems from diverse etiologies, including trauma (Qiao et al, 2024), drug toxicity (Weston et al, 1986), alcohol abuse (Haller and Drachman, 1980), extreme physical exertion (Rawson et al, 2017), and high fiver (Luan et al, 2023). While the initiating factors differ, they converge on the common pathological outcome of massive Mb release into circulation. Therefore, developing a strategy that directly targets this final common pathogenic mediator, free Mb, holds promise for broad clinical applicability across the spectrum of rhabdomyolysis causes.

Previous studies have shown that Mb plays a critical pathogenic role in the pathogenesis of RM-AKI (Zager, 1995; Zager and Burkhart, 1997). Dialysis can remove the circulating toxins, including free Mb non-specifically. However, it is challenging for dialysis units and professional personnel to reach the accident scene promptly. Is it possible to develop a drug to remove free Mb?

Drawing upon the idea of creating a “molecular shield” in the blood circulation to protect the GFB, the therapeutic protein needs to bind to at least one extracellular free Mb (17.8 kDa), forming a macromolecular complex, which will be physically incapable of passing through the GFB (~69 kDa), thereby physically preventing Mb’s renal toxicity. Acute-phase plasma scavenger molecule Hp is ideal. Firstly, Hp is an α2-globulin mainly synthesized by the liver, and widely present in the serum and other body fluids of humans and many mammals (Polonouski, 1950; Polonovski, 1945). Secondly, Hp can bind Hb in the same globin family as Mb to treat severe hemolytic disease. Thirdly, Mb and Hb have similar structures. Our results indicated that native Hp binds free Mb in serum and kidneys. The endogenous expression level of Hp peaked at 12 h and then gradually decreased in the RM-AKI mouse model (Fig. 1). This ‘rise first, then fall’ dynamic strongly suggests that, after the acute-phase reaction-induced peak, endogenous Hp is gradually depleted due to continued binding with excess Mb. That is, endogenous Hp proteins are depleted and insufficient to neutralize and remove all free Mb. Therefore, we must add additional Hp combined with free Mb to treat RM-AKI mice.

Meanwhile, molecular docking predicted that the Mb-binding functional domain of Hp was the beta-subunit (Hpβ, ~28 kDa). GST (~27 kDa), a supergene family of phase II detoxifying enzymes widely distributed in organisms (Smith and Johnson, 1988), can specifically bind to glutathione (LaVallie et al, 2000), making it suitable for use as a tagged protein based on the physical blocking strategy to increase the weight of the fusion protein. Some studies have shown that GST exerts detoxification and antioxidant functions in eukaryotes by catalyzing the binding of electrophilic groups of harmful substances to the sulfhydryl groups of reduced glutathione (Mazari et al, 2023). Meanwhile, glutathione’s beneficial effects on Hb-associated oxidative damage have been observed (Kirschner-Zilber et al, 1989). Research indicates that glutathione can act as an antioxidant to alleviate damage caused by heme proteins (Zager and Burkhart, 1998). Our research has shown that the GST tag in the GST-Hpβ fusion protein does not affect the Mb-binding function of Hpβ and the function of targeting CD163 to promote Mb clearance, thereby better realizing the innovative therapeutic concept of GST-Hpβ-Mb macromolecular protein complexes blocking Mb from passing through the GFB. The design of a recombinant GST-Hpβ fusion protein to neutralize circulating Mb represents a creative and mechanistically distinct approach compared to traditional antibody-based or small-molecule interventions. However, in this study, the prokaryotic expression of the GST-tag protein has no additional independent beneficial effect in the treatment of the RM-AKI model mice, possibly because the GST fragment on the pGEX-6P-1 vector is derived from Schistosoma, and whether mammalian GST can exert antioxidant effects in mice remains to be determined. Furthermore, the xenogeneic origin of this GST tag raises concerns about immunogenicity, an important factor for the clinical translation of any recombinant protein. While this may be a greater concern for scenarios requiring repeated administration, the risk profile is likely more acceptable for the intended single-dose emergency use in acute settings. At the very least, the Schistosoma-derived GST proteins do not negatively affect therapeutic strategies that utilize recombinant GST-Hpβ fusion proteins to alleviate RM-AKI.

**Figure EV5:**
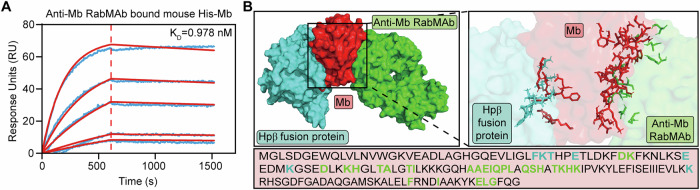
Binding affinity and binding site of anti-Mb RabMAb/GST-Hpβ fusion protein and mouse His-Mb. (**A**) SPR sensorgrams of anti-Mb RabMAb to the recombinant mouse His-Mb immobilized sensor chip. The raw data is shown in blue, and the calculated fit is shown in red. (**B**) Molecular docking predicted the binding sites of anti-Mb RabMAb and GST-Hpβ fusion protein to mouse Mb. Shown below are binding sites on the Mb amino acid sequence that do not coincide with anti-Mb RabMAb (green) and GST-Hpβ fusion protein (blue), respectively.

Hp binds to free Hb released by red blood cells with high affinity (Jayle et al, 1946; Lim et al, 2000), and the Hp-Hb complex was recognized by the macrophage scavenger receptor CD163 (Buehler et al, 2009; Schaer et al, 2013a), thereby promoting the clearance of free Hb (Andersen et al, 2012; Etzerodt et al, 2024). We guess the GST-Hpβ-Mb complex may adopt the same pathway. In our research, we observed co-localization and interaction between the CD163 receptor and the GST-Hpβ-Mb complex in RAW 264.7 cells (Fig. 4E,G). Meanwhile, knocking out CD163 in macrophages or blocking its function with an anti-CD163 antibody will impair macrophages’ phagocytosis of the GST-Hpβ-Mb protein complex (Fig. 4H,I). In addition, since the CD163 receptor is calcium-(Madsen et al, 2004; Xu et al, 2025) and pH-dependent (Gerasimenko et al, 1998), chelating Ca^2+^ with EDTA or lowering the pH to 6.0 can both disrupt the binding of CD163 to the GST-Hpβ-Mb protein complex (Fig. 4H,I). Moreover, GST-Hpβ fusion protein accelerated the phagocytosis of Mb by macrophages (Fig. 4D). The formation of the Hb-Hp complex reduces the harmful oxidative activity of heme/Hb (Nielsen and Moestrup, 2009; Nielsen et al, 2007), prevents the loss of iron ions through the kidney, and protects the kidney from iron ion damage (Baek et al, 2012; Goldenstein et al, 2012). The GST-Hpβ-Mb complex may have a similar effect and prevent heme/Mb- or iron ions-induced kidney damage. Commoditized human plasma-derived Hp is scarce and ethically problematic (Farquhar, 1975). Anti-Mb rabbit monoclonal antibody (anti-Mb RabMAb) is expensive, and there is a risk of allergy to heterologous proteins (Wang et al, 2025). Conversely, recombinant GST-Hpβ proteins are readily available, inexpensive, and endogenous. In addition, from the affinity perspective, the affinity of human recombinant Hp1-1 to human Hb is about 2.3 nM (Nielsen et al, 2006). Anti-Mb RabMAb bound mouse His-Mb with a higher binding affinity (K_D_ = 0.978 nM) (Fig. EV5A). The equilibrium dissociation constant (K_D_) value of GST-Hpβ and mouse His-Mb is 19.3 nM (Fig. 2L). In other words, the GST-Hpβ fusion protein has a strong binding affinity with mouse His-Mb. Meanwhile, molecular docking prediction showed that anti-Mb RabMAb and Mb binding sites were different from those of GST-Hpβ fusion protein and Mb (Fig. EV5B). Whether the combination of GST-Hpβ fusion protein and anti-Mb RabMAb has a better therapeutic effect in RM-AKI animals is worth further exploration. GST-Hpβ protein can be used to treat batched rhabdomyolysis patients in emergency rescue situations. GST-Hpβ can also serve as an in-hospital pharmaceutical formulation to supplement the limited treatment methods available in hospitals. Additionally, it can act as portable medication for soldiers, used for emergency response in the event of rhabdomyolysis occurring in a wartime environment.

The GST-Hpβ fusion protein can remain stable for 3 days at 25 °C in vitro, and it is likely to be completely degraded by 7 days (Fig. 5A). Small animal in vivo imaging experiments of mice show that the GST-Hpβ fusion protein is metabolized or degraded after 3 days (Fig. 6B,C). However, the therapeutic effect of the GST-Hpβ fusion protein, including improvements in kidney function and reduced kidney injury, can last for 14 days (Fig. 5D,K–N). The clearance of Hp protein bound to ligands is generally accomplished through binding to the CD163 receptor on macrophages, leading to degradation of the Hp-ligand protein in the liver or spleen (Schaer et al, 2013b). In addition, since the GST-Hpβ fusion protein contains GST derived from Schistosomes, there may be immunogenicity issues after administration. Therefore, future considerations include creating a new fusion protein (a tandem multi-beta-subunit of Hp) that does not contain the GST tag, while retaining Hpβ’s affinity and large molecular weight. This optimization would directly address the immunogenicity concern while preserving the protein’s core therapeutic mechanism, thereby enhancing its suitability not only for emergency use but also for potential scenarios requiring repeated administration. Moreover, for newly developed recombinant tandem multi-beta-subunit of Hp proteins in drug development, it is necessary to evaluate the circulating half-life and tissue distribution.

Our study has limitations. Firstly, future studies should prioritize direct clinical validation using patient cohorts with rhabdomyolysis (e.g., crush syndrome). Secondly, the GST-Hpβ fusion protein is expressed as insoluble. In the prokaryotic *E. coli* expression system, GST-Hpβ fusion protein can only be purified by inclusion bodies in the precipitation. Moreover, GST derived from Schistosomes has no obvious effect in the RM-AKI model mice, but whether mammalian-derived GST has detoxifying and antioxidant functions in treating RM-AKI needs to be investigated. Thirdly, the tandem multi-beta-subunit of Hp has a large molecular weight, binds more free Mb, and may have a more significant treatment effect than the GST-Hpβ fusion protein in RM-AKI. In fact, this research only completed the proof of concept for treating RM-AKI with the Hpβ fusion protein. Before clinical translation, additional pharmacokinetic and safety studies are required, including a thorough assessment of long-term toxicity and immune responses. Moreover, given that fluid resuscitation is the standard clinical treatment for preserving renal function during rhabdomyolysis, subsequent evaluation of GST-Hpβ or recombinant tandem multi-beta-subunit of Hp proteins, in combination with or compared to standard fluid resuscitation, will be critical for defining their clinical positioning. Fourth, under normal clinical conditions, the timing of administration in this study may be more preventive than therapeutic. Since there is always a time lag between the diagnosis of RM-AKI and medical intervention in clinical practice, it is difficult to administer treatment simultaneously with rhabdomyolysis injury. However, in scenarios with a high incidence of traumatic rhabdomyolysis-AKI (CS-AKI), such as earthquakes, immediately injecting Hpβ proteins after rescuing long-term buried victims may be an effective way to reduce the occurrence of AKI and lower the mortality. Fifth, multiple studies have shown that the Hpβ protein, as a pro-inflammatory factor high mobility group box 1 (HMGB1) antagonist, can bind HMGB1 and sequester its toxicity via a CD163 receptor-dependent mechanism in sterile and infectious inflammation (Morimoto et al, 2022; Yang et al, 2016; Yang et al, 2017). Meanwhile, previous studies have shown that HMGB1 also plays a vital role in RM-AKI (Wang et al, 2021a; Wang et al, 2018). So, besides binding to the critical pathogenic factor Mb, can an exogenous injection of GST-Hpβ further protect RM-AKI mice by binding and inhibiting the pro-inflammatory properties of HMGB1? This question is worth further exploration. In addition, there is a lack of comparison of the therapeutic effects on RM-AKI mice with natural Hp or clinical-grade Hp.

In conclusion, a recombinant GST-Hpβ fusion protein with greater than 95% purity was obtained from the *E. coli* expression system. A single dose of GST-Hpβ fusion protein injection immediately after modeling mitigated RM-AKI model mice by blocking free Mb (17.8 kDa) from crossing the GFB (~69 kDa), and promoted phagocytosis and degradation of GST-Hpβ-Mb protein complex (72.8 kDa) by macrophages through CD163-mediated phagocytosis, which can persist for a minimum of 14 days, significantly surpassing the golden rescue time window (72 h) (Fig. 6D). In the future, GST-Hpβ fusion protein could be a cost-effective way to treat RM-AKI and even excessive Mb release-related diseases. Overall, the therapeutic concept of forming macromolecular protein complexes worked.

## Methods

**Table:** Reagents and Tools Table

Reagent/resource	Reference or source	Identifier or catalog number
**Experimental models**		
Mouse C57BL/6J	SPF (Beijing) Biotechnology Co., Ltd.	N/A
TCMK-1 cells	Servicebio	STCC20015G
RAW 264.7 cells	Servicebio	STCC20020G
BL21(DE3) *E. coli* cells	Zoman Biotech	ZC121
**Recombinant DNA**		
pGEX6p-1	Addgene	27-4597-01
pET-28a(+)	Addgene	69864-3
pET-21a_3xNLS_SpCas9_protein_expression	Addgene	114365
**Antibodies**		
Anti-Mb	Abcam	ab77232
Anti-Mb	Santa Cruz	sc-74525
Anti-NGAL(anti-LCN2)	Affinity	DF6816
Anti-Hp	FineTest	FNab03755
Anti-Hp	HUABIO	ET1703-24
Anti-CD163	BioLegend	155305
Anti-CD163	Santa Cruz	sc-58965
Anti-GST	ZSGB-BIO	TA-03
Anti-GAPDH	Solarbio	K200102M
Anti-Hb	BOSTER	A00233-2
Mouse IgG	Solarbio	SP031
Alexa Fluor^®^ 488 labeled goat anti-rabbit IgG (H + L)	ZSGB-BIO	ZF-0511
Alexa Fluor^®^ 594 -conjugated goat anti-mouse IgG (H + L)	ZSGB-BIO	ZF-0513
Alexa Fluor^®^ 647 labeled goat anti-rat IgG (H + L)	Biosharp	BL062A
Horseradish peroxidase-conjugated goat anti-rabbit IgG (H + L)	ZSGB Biotech	ZB-2301
Horseradish peroxidase-labeled goat anti-mouse IgG (H + L)	ZSGB Biotech	ZB-2305
**Oligonucleotides and other sequence-based reagents**		
Primers	This study	Table EV1
sgRNA	GenScrip	5ʹ-GCGCCGGCUCUGGAUAUAUC-3ʹ
**Chemicals, enzymes and other reagents**		
Serum creatinine (SCr)	Shenzhen Derui Biotechnology Co., Ltd.	GS241 X
Blood urea nitrogen (BUN)	Shenzhen Derui Biotechnology Co., Ltd.	GAHPT
Access Myoglobin Reagent Pack	Beckman Coulter	973243
Atellica CH Haptoglobin (Hapt)	Siemens Healthineers	11097643
TRIeasy™ LS Total RNA Extraction Reagent	YEASEN	19201ES
Hifair® III 1st Strand cDNA Synthesis SuperMix for qPCR(gDNA digester plus)	YEASEN	11141ES
Hieff^®^ qPCR SYBR^®^ Green Master Mix (No Rox)	YEASEN	11201ES
RIPA buffer	Solarbio	R0010
Enhanced ECL Chemiluminescent Substrate Kit	YEASEN	36222ES
Mouse Haptoglobin ELISA kit	WEIAOBIO	EM31040S
FITC-sinistrin	MediBeacon	S10010005
Inulin-FITC	Sigma	F3272
Small patch	MediBeacon	S10010007
4% paraformaldehyde	Solarbio	P1110
Hematoxylin-Eosin/HE Staining Kit	Solarbio	G1120
Masson’s Trichrome Stain Kit	Solarbio	G1340
DAB substrate kit	ZLI-9019	ZSGB-BIO
Mounting Medium, antifading (with DAPI)	Solarbio	S2110
Cell lysis buffer for Western and IP	Beyotime	P0013
Protein A/G Magnetic Beads	Selleck	B23202
IPTG (Isopropyl β-D-Thiogalactopyranoside)	Solarbio	I8070
GSTSep Glutathione Agarose Resin	YEASEN	20507ES
Ni Sepharose^TM^ 6 Fast Flow	Cytiva	17-5318-06
HiTrap® SP High Performance	Cytiva	17-1152-01
Carboxyl chip	XLement	G70004
Penicillin-Streptomycin Liquid	Solarbio	P1400
Cell Counting Kit (CCK-8) CCK-8	YEASEN	40203ES
Dynasore	Aladdin	D125461
P3 buffer	Lonza	V4XP-3032
VeriBlot for IP Detection Reagent (HRP)	Abcam	ab131366
EGTA buffer	PSAITONG	PS0315
Recombinant Mouse IL-4 Protein	ABclonal	RP01161
**Software**		
MediBeacon Studio 2	MediBeacon	
Tanon, 2.0.1	Tanon ABL-X5 In Vivo Imaging System	
Prism 8	Graphpad by Dotmatics	https://www.graphpad.com
ImageJ	ImageJ	https://imagej.net/ij/
PymoL	Schrödinger	
Adobe Illustrator 2020	Adobe	www.adobe.com
**Other**		
WeSPR^TM^ 200 SPR instrument	XLement	
Tanon 5200 Multi	Tanon	

### Animals and RM-AKI model establishment

C57BL/6J mice (male, weighing 20–22 g, 8 weeks old) were housed in a pathogen-free environment with a 12 h light/dark cycle and provided free food and water. All mice were randomly divided into different groups. For the glycerol-induced RM-AKI model, the C57BL/6 J mice were anesthetized and injected intramuscularly into both hindlimb skeletal muscles with glycerol (8.0 mL/kg, 50% in sterile saline). Meanwhile, an equivalent volume of saline was used as the normal control (NC). The mice were euthanized at 6, 12, 24, and 48 h after modeling. For the CS-AKI model, after C57BL/6 J mice were anesthetized, the compression device was used to apply 1.5 kg of pressure to the bilateral hindlimb for 16 h. The NC group of mice was not subjected to any pressure. The mice were euthanized 24 h after decompression. Groups for each time point were set up with six mice per group. Animal experiments were approved by the Animal Ethical and Welfare Committee of Tianjin University (NO. TJUE-2022–279).

### GST-Hpβ fusion protein treatment

GST-Hpβ fusion proteins were administered by intravenous (i.v.) or intraperitoneal (i.p.) to the mice immediately after modeling. For the glycerol-induced RM-AKI model experiment, the groups were as follows: Saline group (normal control), Gly group (glycerol-induced RM-AKI), Gly + GST group (GST, 10 mg/kg), Gly + GST-Hpβ 10 mg/kg group (GST-Hpβ, 10 mg/kg), Gly + GST-Hpβ 20 mg/kg group (GST-Hpβ, 20 mg/kg), and Gly + GST-Hpβ 40 mg/kg group (GST-Hpβ, 40 mg/kg). Proteins were stored in a PBS buffer. The Saline and Gly groups were not treated with drug administration. For the CS-AKI experiment, the groups include the NC group (normal control), the CS group (CS-AKI), the CS + GST group (GST, 10 mg/kg), and the CS + GST-Hpβ group (GST-Hpβ, 10 mg/kg). Six mice were used in each group. After 24 h of protein treatment, mice were sacrificed, and samples were collected for subsequent testing.

### Serum biochemistry

Serum creatinine (SCr), blood urea nitrogen (BUN), Mb, and Hp levels were measured using an automatic biochemical analyzer (iMagic-V 7, Icubio). The testing process is carried out according to the manufacturer’s instructions (Shenzhen Derui Biotechnology Co., Ltd, #GS241 X, #GAHPT; Beckman Coulter, #973243; Siemens Healthineers, #11097643).

### Quantitative real-time PCR (qPCR)

The total RNA from kidney tissue was extracted using TRIeasy™ LS Total RNA Extraction Reagent (YEASEN, #19201ES) and reverse-transcribed into cDNA for PCR amplification according to the manufacturer’s protocols (YEASEN, #11141ES). LightCycler^®^ 96 instrument (Roche) performed the qPCR reaction with Hieff^®^ qPCR SYBR^®^ Green Master Mix (No Rox) (YEASEN, #11201ES). The 2^−ΔΔCt^ method was used to calculate relative gene expression. The primer sequences are listed in Table EV1.

### Western blot

Proteins were extracted using RIPA buffer (Solarbio, #R0010). The protein samples were electrophoresed by SDS-PAGE and transferred to PVDF membranes. The membranes were blocked with 5% skimmed milk for 1.5 h at room temperature before incubation with anti-Mb (Abcam, #ab77232, 1:5000 dilution), anti-NGAL (Affinity, #DF6816, 1:2000 dilution), anti-Hp (FineTest, #FNab03755, 1:2000 dilution), and anti-GAPDH (Solarbio, #K200102M, 1:5000 dilution) primary antibodies at 4 °C overnight. The membranes were incubated with HRP-conjugated secondary antibodies (ZSGB Biotech, #ZB-2301, #ZB-2305, 1:5000 dilution) for 1.5 h. The protein bands were visualized with the Enhanced ECL Chemiluminescent Substrate Kit (YEASEN, #36222ES) on Tanon 5200 Multi automatic chemiluminescence/fluorescence image analysis system. The intensity of each band was measured using the Tanon Gel-Pro Analyzer system.

### ELISA assay for serum Hp level

Hp level was determined using an ELISA kit (WEIAOBIO, #EM31040S) according to the manufacturer’s protocols. First, the serum samples were added to the enzyme scale plate. Subsequently, enzyme-labeling reagents (primary and secondary antibodies) were added and incubated at 37 °C. After that, the chromogenic solution was added and incubated for 15 min at 37 °C in the dark. A stop buffer was added, and the OD value was quickly measured at 450 nm with the Microplate Reader (BioTek, Gen5).

### Glomerular filtration rate (GFR) measurement

A Stock solution of 28 mg/mL FITC-sinistrin (MediBeacon, #S10010005) or Inulin-FITC (Sigma, F3272) in sterile normal saline was prepared as a fluorescent tracer (prepared immediately before use). GFR was measured in mice using transcutaneous FITC-sinistrin/Inulin-FITC disappearance with the GFR monitor (MediBeacon GmbH, Mannheim, Germany). Mice were shaved to expose the skin. The imager device was mounted, and the FITC-sinistrin/Inulin-FITC (7 mg/100 g b.w.) was injected through the tail vein after collecting the basic signal for 5 min. After 2 h, the devices were removed, and the data were analyzed using the Studio software.

### Histology

The mouse tissues were fixed in 4% paraformaldehyde (Solarbio, #P1110), dehydrated in a graded alcohol series, and embedded in paraffin. Then, the tissues were cut into 4 μm-thick sections and stained with Hematoxylin-Eosin (HE) Stain Kit (Solarbio, #G1120) and the Masson Stain Kit (Solarbio, #G1340). The pathological changes in the tissue were observed using light microscopy.

### Immunofluorescence and immunohistochemistry

After dewaxing and rehydration, paraffin sections were incubated with 3% hydrogen peroxide, heated in a microwave to repair antigens, and blocked with goat serum. For immunohistochemical analysis, sections were labeled with primary antibodies against Mb (Santa Cruz, #sc-74525, 1:100 dilution) and Hp (FineTest, #FNab03755, 1:100 dilution), then incubated with HRP-conjugated secondary antibodies at 37 °C for 1 h. Then, sections were stained with DAB, nuclei with hematoxylin, and images were acquired by light microscopy. For immunofluorescence analysis, sections labeled with primary antibodies against Mb (Santa Cruz, #sc-74525, 1:100 dilution), Hp (FineTest, #FNab03755, 1:100 dilution), and then incubated with secondary antibodies coupled to Alexa Fluor^®^ 488/594 (ZSGB-BIO, #ZF-0511, #ZF-0513, 1:200 dilution) at room temperature for 1 h. Nuclei stained with 4’,6-diamidino-2-phenylindole (DAPI, Solarbio, #S2110) and observed by fluorescence microscopy (OLYMPUS, #DP74).

For cell samples, TCMK-1 and RAW 264.7 cells were inoculated on a glass cover slide in a 24-well plate (NEST Biotechnology Co. Ltd., #702002), fixed with 4% paraformaldehyde (Solarbio, #P1110) for 20 min, then permeated with 0.3%Triton X-100 for 10 min and sealed with 5% bovine serum albumin (BSA) at room temperature for 1 h. Then, cells were incubated with primary antibodies against Mb (Santa Cruz, #sc-74525, 1:100 dilution), Hp (FineTest, #FNab03755, 1:100 dilution), and CD163 (BioLegend, #155305, 1:100 dilution) at 4 °C overnight. Next, incubated with Alexa Fluor 488/594/647 conjugated secondary antibodies (ZSGB-BIO, #ZF-0511, #ZF-0513, Biosharp, #BL062A, 1:200 dilution) at room temperature for 1 h. Then, cells were finally sealed with 4’,6-diamidino-2-phenylindole (DAPI, Solarbio, #S2110). At last, images were captured using a fluorescence or confocal microscope (Nikon, A1).

### Co-immunoprecipitation (Co-IP)

Total proteins of mouse kidney tissue or RAW 264.7 cells were extracted using IP lysis buffer (Beyotime, #P0013), and incubated overnight at 4 °C with anti-Mb antibody (Santa Cruz, #sc-74525; Abcam, #ab77232) or anti-Hp antibody (FineTest, #FNab03755; HUABIO, #ET1703-24) or anti-CD163 antibody (Santa, #sc-58965) and Protein A/G Magnetic Beads (Selleck, #B23202) with shaking. The bound proteins were subjected to Western blot analysis.

### Molecular docking

The tertiary structure of GST-Hpβ and Mb was obtained from the AlphaFold Protein Structure Database (https://alphafold.ebi.ac.uk/). The binding amino acid sites between the wild-type Hp/ recombinant GST-Hpβ fusion protein and Mb were predicted using HDOCK (http://hdock.phys.hust.edu.cn/) and PyMOL (v 3.0). The standard program defaults were used during the run. All illustrations were prepared in PyMOL.

### Expression and purification of GST-Hpβ fusion protein and GST protein

The Hpβ subunit (residues 162-406 of Hp, NP_005134.1) was expressed in BL21(DE3) *E. coli* cells (Zoman Biotech, #ZC121) using the pGEX-6P-1 plasmid (Addgene, #27-4597-01), and 0.2 mM IPTG (Solarbio, #I8070) at 37 °C overnight. The *E. coli* cells were harvested and lysed. The clarified lysate was added to GST-affinity columns (YEASEN, #20507ES), and the fusion protein was stored in PBS (pH 7.4). The purified proteins were identified by Coomassie blue staining and western blot with anti-GST antibody (ZSGB-BIO, #TA-03) and anti-Hp antibody (FineTest, #FNab03755).

For Cas9 (Addgene, #114365) protein purification, the supernatant was subjected to purification using Ni Sepharose 6 Fast Flow (Cytiva, #17-5318-06) filler with washing buffer A (20 mM Tris-HCl pH 7.5, 500 mM NaCl, 25 mM and 50 mM imidazole), washing buffer B (20 mM Tris-HCl pH 7.5, 200 mM NaCl, 25 mM and 50 mM imidazole) and the elution buffer C (20 mM Tris-HCl pH 7.5, 200 mM NaCl, 350 mM imidazole). The eluted protein was further purified using a HiTrap SP HP (Cytiva, #17-1152-01) column with buffer D (20 mM Tris-HCl pH 7.5, 100 mM NaCl, 5% glycerol) and buffer E (20 mM Tris-HCl pH 7.5, 1 M NaCl, 5% glycerol). The peak pools containing proteins were combined, concentrated, flash-frozen in liquid nitrogen, and stored at −80 °C until further use.

### His/GST pulldown

His-Mb and GST-Hpβ fusion protein is incubated at 4 °C for 30 min, then 10 μL His/GST resin (Cytiva, #17-5318-06; YEASEN, #20507ES) is added for another 30 min at 4 °C. Centrifuge at 12,000 rpm for 5 min and remove the supernatants. Use 200 μL buffer to wash the beads 3-4 times, removing unbound protein. The bound proteins were tested by Western blot.

### Surface plasmon resonance (SPR)

The affinity between GST-Hpβ fusion protein and His-Mb was measured using a WeSPR^TM^ 200 SPR instrument (XLement). All proteins were exchanged into the running buffer, 0.01 M PBS (2 mM KH_2_PO_4_, 8 mM Na_2_HPO_4_·12H_2_O, 136 mM NaCl, 2.6 mM KCl, adjusted to pH 7.4). His-Mb protein was immobilized on a carboxyl chip (XLement, #G70004) surface via coupling. Gradient concentrations of GST-Hpβ fusion protein (from 50 μg/mL to 6.25 μg/mL with 2-fold dilution) flowed over the chip surface. The data were collected, and the affinity was calculated.

### In vivo mouse imaging

The in vivo biodistribution of Cy5-GST-Hpβ protein was performed using a small animal live imaging system (Tanon, ABL X5) at various time points (0, 1, 3, 24, 48, and 72 h) following a single Cy5-GST-Hpβ (10 mg/kg) administration. Total fluorescence intensity data were analyzed from selected areas using the system’s software (Tanon, 2.0.1).

### Cell culture and cell viability

Mouse tubular epithelial cells (TECs) TCMK-1 (Servicebio, #STCC20015G) and mouse mononuclear macrophage cells RAW 264.7 (Servicebio, #STCC20020G) were cultivated in DMEM medium with high glucose levels, supplemented with 10% FBS and a mixture of antibiotics, including penicillin (100 IU/mL) and streptomycin (100 mg/mL) at 37 °C in 5% CO_2_. The vendor confirmed that all cell lines are mycoplasma-negative. The RAW264.7 cell line was confirmed as STR profiled by the vendors.

To simulate the RM-AKI model in vitro, we collected cells during their logarithmic growth phase and exposed them to 200 μM mouse His-Mb. TCMK-1 cells were inoculated into 96-well plates for 24 h to assess their viability. Cells were subjected to increasing concentrations of mouse His-Mb (ranging from 25 to 800 μM) for 24 h. Cells were then incubated with the CCK-8 reagent (YEASEN, #40203ES) for 1 h. Subsequently, absorbance readings corresponding to optical density (OD) at 450 nm were obtained using a Microplate Reader (BioTek, Gen5).

### Uptake of Mb and Hpβ complexes

To identify the cell uptake process of Mb and GST-Hpβ complexes, TCMK-1 cells or mouse macrophage cells RAW 264.7 were inoculated onto coverslips separately. Then, the cells were incubated with mouse His-Mb for 6 h at 37 °C with or without GST-Hpβ protein. In one group, RAW 264.7 cells were pre-treated with endocytosis inhibitor Dynasore (Aladdin, #D125461, 80 μM) for 30 min at 37 °C. Following incubation, cells were fixed with 4% paraformaldehyde (Solarbio, #P1110) for 20 min at ambient temperature. Then, immunofluorescence validated the uptake of the GST-Hpβ-Mb complex.

### Generation of *Cd163*-KO RAW264.7 cell line using CRISPR-Cas9 technique

A single-guide RNA (sgRNA) targeting mouse *Cd163* (5ʹ-GCGCCGGCUCUGGAUAUAUC-3ʹ) was designed and synthesized (GenScript). The sgRNA was complexed with recombinant Cas9 protein at a 2:1 molar ratio and incubated for 25 min at room temperature to form ribonucleoprotein (RNP) complexes. RAW264.7 cells were then electroporated with the RNP complexes using the 4D Nucleofector^®^ system (Lonza) with program CM-138 in 20 μL P3 buffer (Lonza, V4XP-3032) per reaction. The media was changed to full growth medium after 24 h, and the cells were subjected to limiting dilution to obtain monoclonal cell populations.

### Quantification and statistical analysis

No animal, sample, or data were excluded from the analysis. Experimental animal groups were assigned randomly to vehicle or treatment groups. No blinding was performed. The number of samples included in each experiment is indicated in the figure legends. All experiments were conducted with at least three biological replicates. Statistical analysis was conducted using One-Way analysis of variance (ANOVA) for datasets with more than two groups. The statistics reflect mean ± SD from at least three independent trials. The tests were based on the assumptions of independent samples, equal variances, and normal distribution. Differences were significant when *P*  <  0.05.

## Supplementary information

Table EV1

Peer Review File

Expanded View Figures

## Data Availability

This study includes no data deposited in external repositories. The source data are available in the following database: biostudies: S-BSST2964. The source data of this paper are collected in the following database record: biostudies:S-SCDT-10_1038-S44321-026-00454-0.
