# Supplementary material for: Engineered haptoglobin β fusion protein targets myoglobin and ameliorates rhabdomyolysis-associated acute kidney injury
Source: EMBO Mol Med. 2026 May 25;18(7):2723–47. doi: 10.1038/s44321-026-00454-0 (PMC13365507; doi:10.1038/s44321-026-00454-0)
Supplement: Supplementary file 1 — Table EV1 [file 44321_2026_454_MOESM1_ESM.docx]

Table EV1. Primer sequences for qPCR.

| **Primers:** | **Sequence Forward (5'-3')** | **Sequence Reverse (5'-3')** |
| --- | --- | --- |
| **Genes for Figure 3D-E** |  |  |
| *Kim-1* | ACATATCGTGGAATCACAACGAC | ACAAGCAGAAGATGGGCATTG |
| *Ngal* | TGGCCCTGAGTGTCATGTG | CTCTTGTAGCTCATAGATGGTGC |
| *Gapdh* | AGGTCGGTGTGAACGGATTTG | TGTAGACCATGTAGTTGAGGTCA |
|  |  |  |
| **Genes for Figure 3N-S** |  |  |
| *Vcam-1* | AGCTCATGAACAGACAGGAG | GGTAGACCCTCGCTGGAAC |
| *Gapdh* | AGGTCGGTGTGAACGGATTTG | TGTAGACCATGTAGTTGAGGTCA |
