# Supplementary material for: Engineered haptoglobin β fusion protein targets myoglobin and ameliorates rhabdomyolysis-associated acute kidney injury
Source: EMBO Mol Med. 2026 May 25;18(7):2723–47. doi: 10.1038/s44321-026-00454-0 (PMC13365507; doi:10.1038/s44321-026-00454-0)
Supplement: Supplementary file 3 — Expanded View Figures [file 44321_2026_454_MOESM3_ESM.pdf]

Expanded View Figures

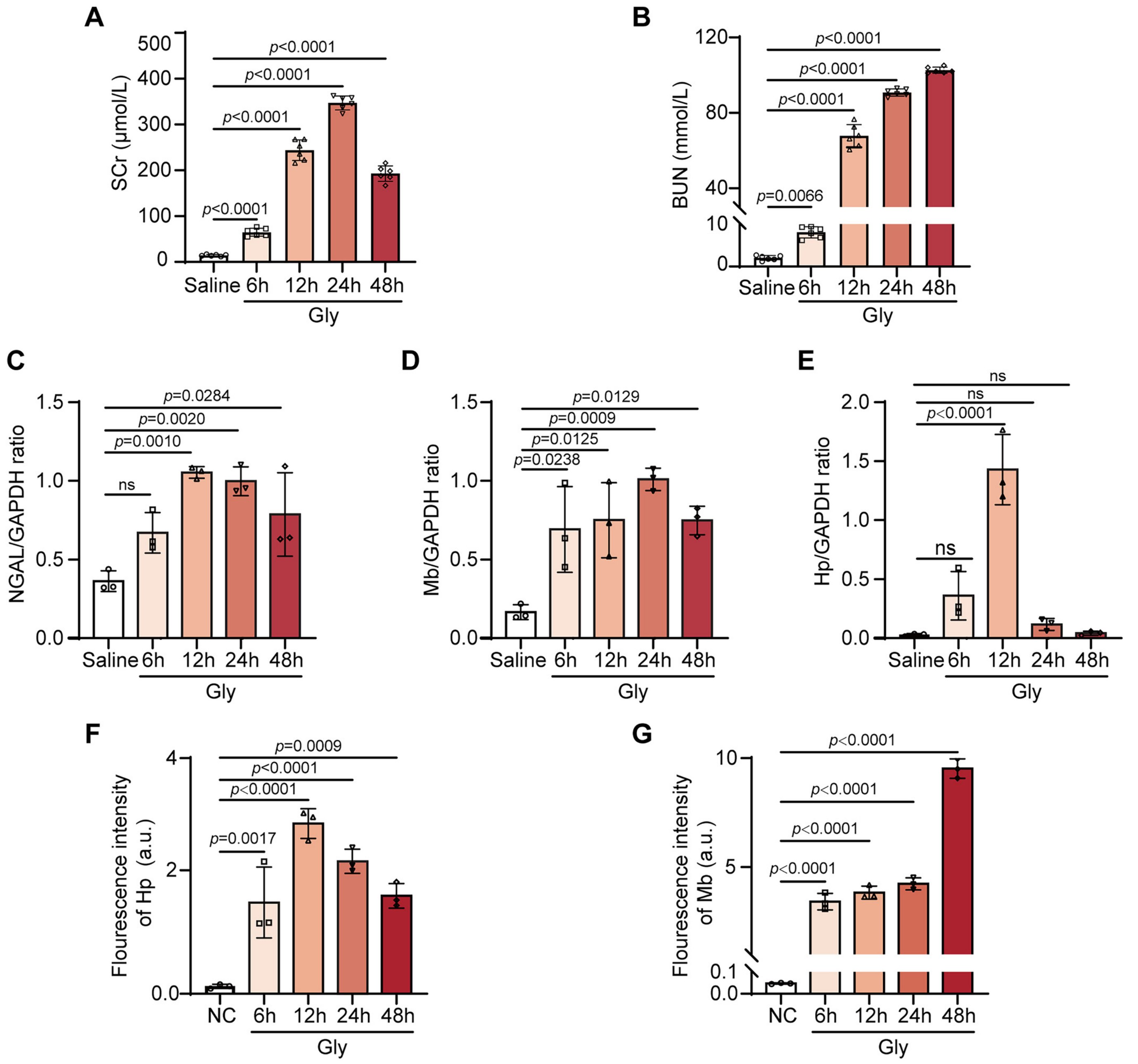

Figure EV1. Mb and Hp expression in the kidney of RM-AKI mice.

(**A**, **B**) Blood biochemical analysis of the serum concentrations of SCr (**A**) and BUN (**B**) in the glycerol-induced RM-AKI mouse model ($n = 6$ per group for biological replicates). (**C–E**) The grey values of the protein levels of NGAL bands (**C**), Mb bands (**D**), and Hp bands (**E**) from Western blot (Fig. 1B) were employed for statistical analysis ($n = 3$ for biological replicates). (**F**, **G**) Quantification of Immunofluorescence data of Hp (**F**) and Mb (**G**) expression in Fig. 1I ($n = 3$ per group for biological replicates). For statistical analysis, the one-way ANOVA (**A–G**) was used. Data are expressed as mean ± SD. $P < 0.05$ was considered statistically significant. ns not significant.

© The Author(s)

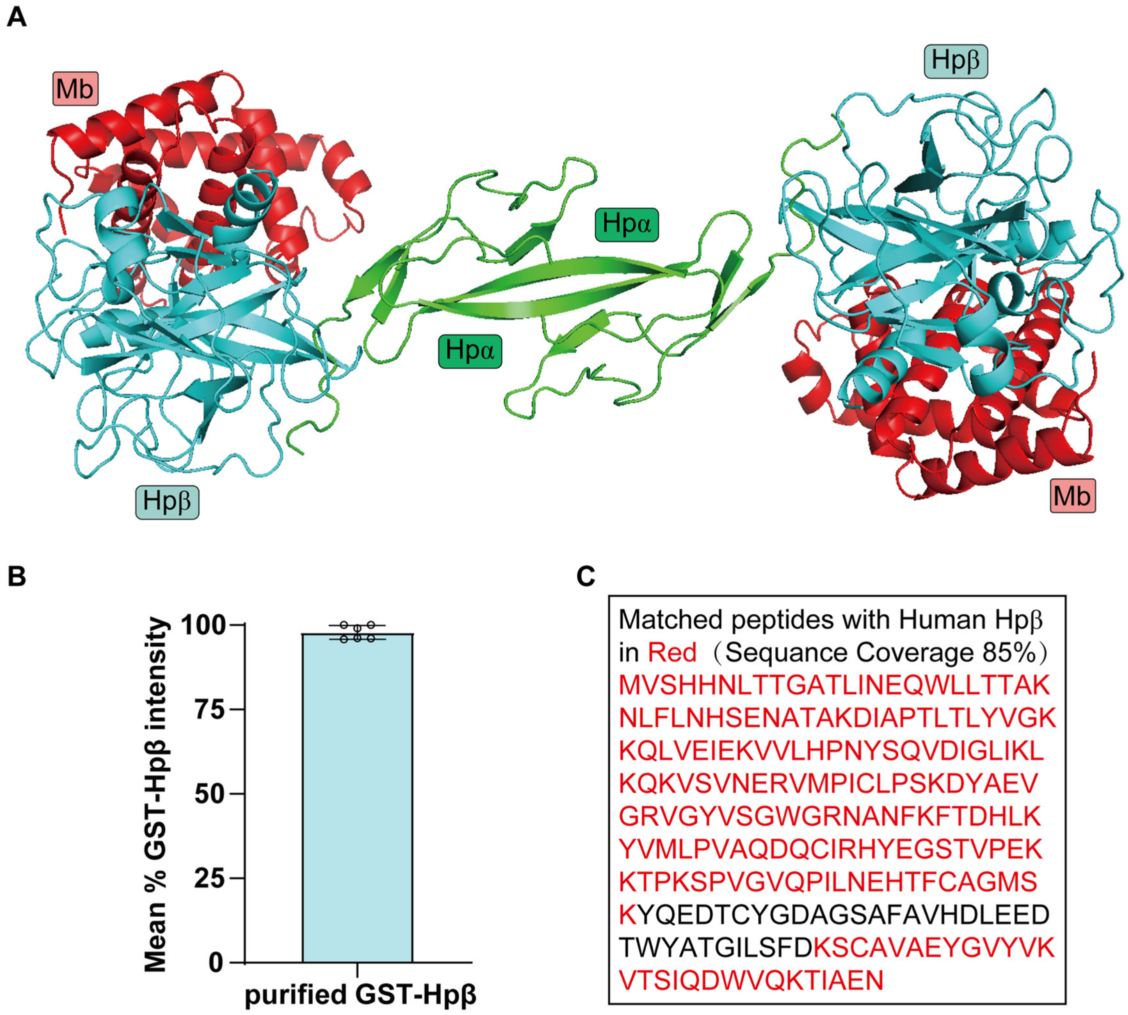

Figure EV2. Mb binding region focuses on Hp beta-subunit (Hpβ) predicted by molecular docking and the identification of recombinant GST-Hpβ fusion protein.

(**A**) Molecular docking of the tetramers Hp protein and Mb using HDOCK. PyMOL was used to visualize the binding site for the best-scoring docking poses. (**B**) Quantitative analysis of gray values of the GST-Hpβ fusion protein in Fig. 2E,F by ImageQuant™ TL analysis software. The purity of recombinant GST-Hpβ fusion protein was greater than 95% ($n = 6$ for biological replicates). Data are expressed as mean ± SD. (**C**) Protein mass spectrometry analysis for purified GST-Hpβ fusion protein.

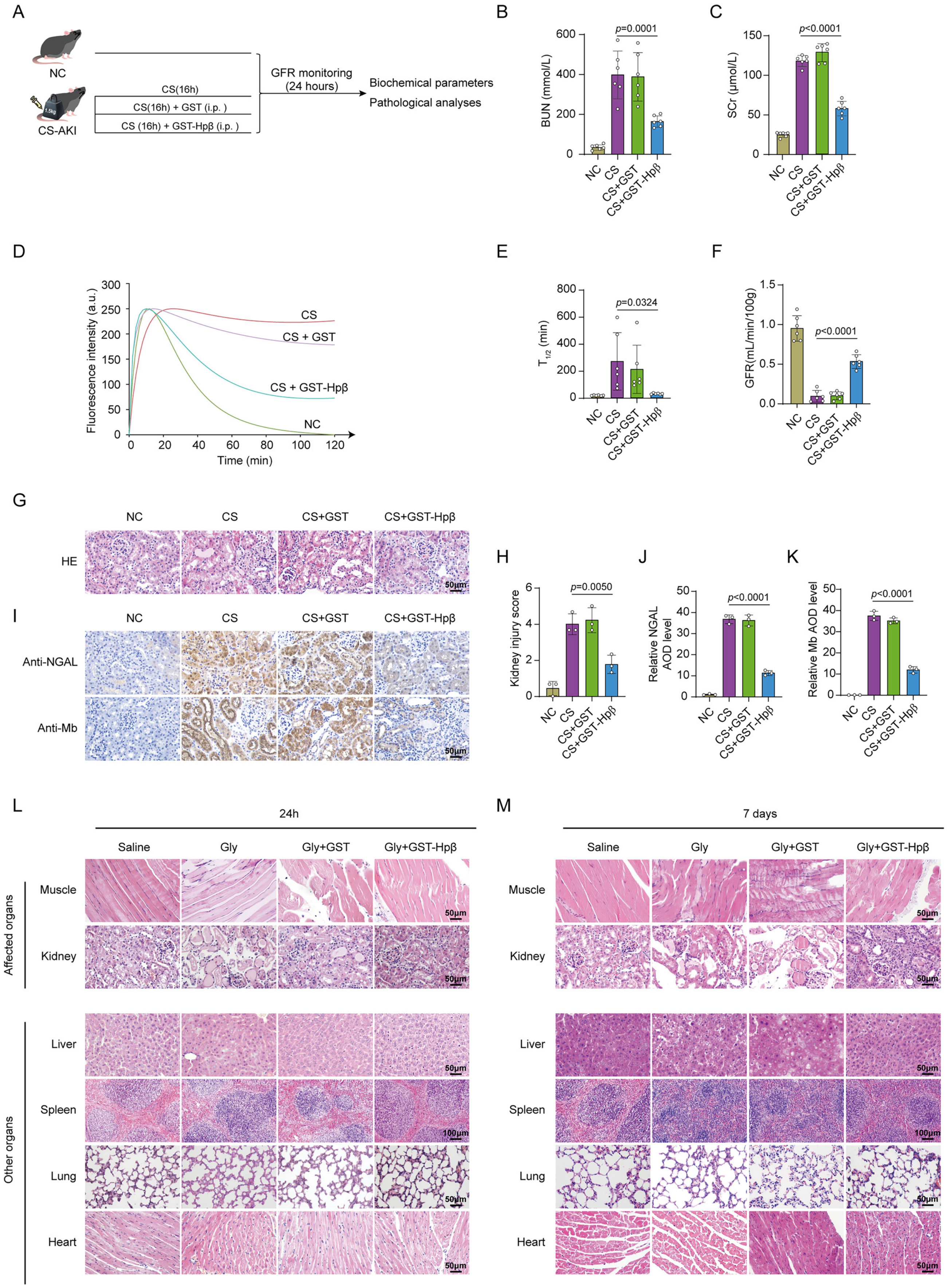
A
NC
CS-AKI
CS(16h)
CS(16h) + GST (i.p.)
CS (16h) + GST-Hpβ (i.p.)
GFR monitoring (24 hours)
Biochemical parameters
Pathological analyses
B
BUN (mmol/L)
p=0.0001
C
SCr (μmol/L)
p<0.0001
D
Fluorescence intensity (a.u.)
Time (min)
CS
CS + GST
CS + GST-Hpβ
NC
E
T1/2 (min)
p=0.0324
F
GFR(mL/min/100g)
p<0.0001
G
HE
I
Anti-NGAL
Anti-Mb
H
Kidney injury score
p=0.0050
J
Relative NGAL AOD level
p<0.0001
K
Relative Mb AOD level
p<0.0001
NC
CS
CS+GST
CS+GST-Hpβ
L
24h
M
7 days
Saline
Gly
Gly+GST
Gly+GST-Hpβ
Affected organs
Other organs
Muscle
Kidney
Liver
Spleen
Lung
Heart
50μm
100μm

Figure EV3. Therapeutic effects of GST-Hpβ on CS-AKI mice and pathological examination of major organs in glycerol-induced RM-AKI mice.

(**A**) Experimental design of GST-Hpβ fusion protein treatment for CS-AKI mice. Mice were subjected to 1.5 kg of compression for 16 h, and GST-Hpβ was injected immediately after decompression. Kidney function in the mice was tested 24 h later, blood samples were collected for biochemical examination, and tissue samples were collected for pathological analysis. (**B**, **C**) Blood biochemical analysis of the concentrations of BUN (**B**) and SCr (**C**) in serum in NC and CS-AKI mice ($n = 6$ per group for biological replicates). (**D**) Representative image of transcutaneous disappearance curves of Inulin-FITC excretion after 24 h in i.p. injection with 10 mg/kg GST-Hpβ of CS-AKI mice. (**E**) The excretion half-life ($T_{1/2}$) of Inulin-FITC in NC, CS, CS + GST, and CS + GST-Hpβ groups of mice ($n = 6$ per group for biological replicates). (**F**) GFR of mice in NC, CS, CS + GST, and CS + GST-Hpβ groups was measured at 24 h ($n = 6$ per group for biological replicates). (**G**) HE staining assesses renal tubular injury severity in selected NC, CS, CS + GST, and CS + GST-Hpβ groups. (**H**) Blinded kidney injury score of the selected groups of mice in the image of (**G**) ($n = 3$ per group for biological replicates). (**I**) Representative IHC staining images of Mb and NGAL expression in kidney tissues of NC, CS, CS + GST, and CS + GST-Hpβ groups. (**J**, **K**) Quantification of the integrated optical density (IOD) of NGAL (**J**) and Mb (**K**) in the image of (**I**) ($n = 3$ per group for biological replicates). (**L**, **M**) HE staining was used to analyze the injury in the affected organs (muscle, kidney) and other organs (liver, spleen, lung, and heart) in glycerol-induced RM-AKI model mice after injection of recombinant GST-Hpβ fusion protein for 24 h or 7 d. For statistical analysis, the one-way ANOVA was used (**B**, **C**, **E**, **F**, **H**, **J**, **K**). Data are expressed as mean ± SD. $P < 0.05$ was considered statistically significant. ns not significant.

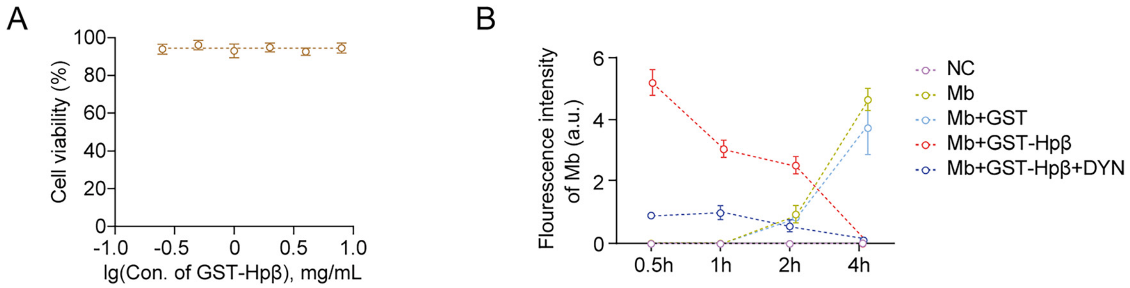

Figure EV4. Toxicity of GST-Hpβ to TCMK-1 cells and quantification of confocal microscopy images of RAW 264.7 cells metabolism of Mb.

(**A**) CCK-8 detects the survival rate of TCMK-1 cells treated with different concentrations (0.25 mg/mL, 0.5 mg/mL, 1 mg/mL, 2 mg/mL, 4 mg/mL, and 8 mg/mL) of GST-Hpβ fusion protein. (**B**) Quantification of confocal microscopy images of RAW 264.7 cells metabolism of Mb in Fig. 4D. Quantitative analysis of the fluorescence intensity of mouse His-Mb proteins in confocal microscopy images of RAW 264.7 cells for different times (0.5, 1, 2, 4 h). Results are presented as mean ± SD, and dots indicate individual quantitative analysis data points from three biological replicates.

 © The Author(s)

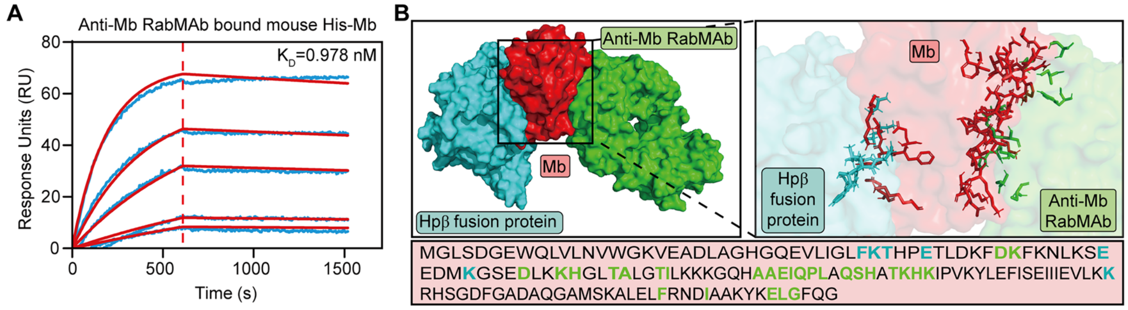

Figure EV5. Binding affinity and binding site of anti-Mb RabMAb/GST-Hpβ fusion protein and mouse His-Mb.

(**A**) SPR sensorgrams of anti-Mb RabMAb to the recombinant mouse His-Mb immobilized sensor chip. The raw data is shown in blue, and the calculated fit is shown in red. (**B**) Molecular docking predicted the binding sites of anti-Mb RabMAb and GST-Hpβ fusion protein to mouse Mb. Shown below are binding sites on the Mb amino acid sequence that do not coincide with anti-Mb RabMAb (green) and GST-Hpβ fusion protein (blue), respectively.

© The Author(s)
