# Supplementary material for: Engineered haptoglobin β fusion protein targets myoglobin and ameliorates rhabdomyolysis-associated acute kidney injury
Source: EMBO Mol Med. 2026 May 25;18(7):2723–47. doi: 10.1038/s44321-026-00454-0 (PMC13365507; doi:10.1038/s44321-026-00454-0)
Supplement: Supplementary file 2 — Peer Review File [file 44321_2026_454_MOESM2_ESM.pdf]

EMBO Molecular Medicine

Engineered haptoglobin β fusion protein targets myoglobin and ameliorates rhabdomyolysis-associated acute kidney injury

Ning Li, Yuru Wang, Lu Han, Ou Qiao, Xinyue Wang, Herui Hao, Xin Chen, Pengtao Wang, Sania Saeed, Jing Wang, Fengjiao Bao, Yingjie Hou, Li Zhang, Xiaohong Duan, Shuquan Rao, and Yanhua Gong

Corresponding author(s): Yanhua Gong (gongyanhua@tju.edu.cn) , Shuquan Rao (raoshuquan@ihcams.ac.cn)

Review Timeline:

Submission Date:	9th Oct 25
Editorial Decision:	23rd Oct 25
Revision Received:	20th Jan 26
Editorial Decision:	2nd Mar 26
Revision Received:	13th Apr 26
Editorial Decision:	28th Apr 26
Revision Received:	8th May 26
Accepted:	11th May 26

Editor: Zeljko Durdevic

Transaction Report:

(Note: With the exception of the correction of typographical or spelling errors that could be a source of ambiguity, letters and reports are not edited. Depending on transfer agreements, referee reports obtained elsewhere may or may not be included in this compilation. Referee reports are anonymous unless the Referee chooses to sign their reports.)

23rd Oct 2025

Dear Prof. Gong,

Thank you for the submission of your manuscript to EMBO Molecular Medicine. We have now received feedback from the three reviewers who agreed to evaluate your manuscript.

As you will see from the reports pasted below, while referees #1 and #2 recognize potential interest of the manuscript and raise important and partially overlapping concerns particularly regarding the lack of pharmacokinetic and toxicity evaluation, referee #3 raises concerns about clinical rhabdomyolysis and murine models. Based on the referee reports I would like to invite major revision of the present manuscript, with the understanding that all referee concerns should be addressed. Referee #3 point about clinical rhabdomyolysis model should be addressed by discussion. If you would like to discuss further the points raised by the referees, I am available to do so via email or video. Let me know if you are interested in this option.

Further consideration of a revision that addresses reviewers' concerns in full will entail a second round of review. EMBO Molecular Medicine encourages a single round of revision only and therefore, acceptance or rejection of the manuscript will depend on the completeness of your responses included in the next, final version of the manuscript. For this reason, and to save you from any frustrations in the end, I would strongly advise against returning an incomplete revision.

We would welcome the submission of a revised version within three months for further consideration. Please let us know if you require longer to complete the revision.

I look forward to receiving your revised manuscript.

Yours sincerely,

Zeljko Durdevic

Zeljko Durdevic
Senior Editor
EMBO Molecular Medicine

*****

When submitting your revised manuscript, please carefully review the instructions that follow below. We perform an initial quality control of all revised manuscripts before re-review; failure to include requested items will delay the evaluation of your revision.

We require:

1) A .docx formatted version of the manuscript text (including legends for main figures, EV figures and tables). Please make sure that the changes are highlighted to be clearly visible.

2) Individual production quality figure files as .eps, .tif, .jpg (one file per figure). For guidance, download the 'Figure Guide PDF': (https://www.embopress.org/page/journal/17574684/authorguide#figureformat).

3) A .docx formatted letter INCLUDING the reviewers' reports and your detailed point-by-point responses to their comments. As part of the EMBO Press transparent editorial process, the point-by-point response is part of the Review Process File (RPF), which will be published alongside your paper.

4) A complete author checklist, which you can download from our author guidelines (https://www.embopress.org/page/journal/17574684/authorguide#submissionofrevisions). Please insert information in the checklist that is also reflected in the manuscript. The completed author checklist will also be part of the RPF.

5) Please note that all corresponding authors are required to supply an ORCID ID for their name upon submission of a revised manuscript.

6) It is mandatory to include a 'Data Availability' section after the Materials and Methods. Before submitting your revision, primary datasets produced in this study need to be deposited in an appropriate public database, and the accession numbers and database listed under 'Data Availability'. Please remember to provide a reviewer password if the datasets are not yet public (see https://www.embopress.org/page/journal/17574684/authorguide#dataavailability).

In case you have no data that requires deposition in a public database, please state so in this section. Note that the Data Availability Section is restricted to new primary data that are part of this study.

7) For data quantification: please specify the name of the statistical test used to generate error bars and P values, the number (n) of independent experiments (specify technical or biological replicates) underlying each data point and the test used to calculate p-values in each figure legend. The figure legends should contain a basic description of n, P and the test applied. Graphs must include a description of the bars and the error bars (s.d., s.e.m.). See also 'Figure Legend' guidelines: https://www.embopress.org/page/journal/17574684/authorguide#figureformat

8) At EMBO Press we ask authors to provide source data for the main manuscript figures. You will receive a separate email with instructions for providing source data with your revised manuscript, including how to upload and organize the files.

9) Our journal encourages inclusion of *data citations in the reference list* to directly cite datasets that were re-used and obtained from public databases. Data citations in the article text are distinct from normal bibliographical citations and should directly link to the database records from which the data can be accessed. In the main text, data citations are formatted as follows: "Data ref: Smith et al, 2001" or "Data ref: NCBI Sequence Read Archive PRJNA342805, 2017". In the Reference list, data citations must be labeled with "[DATASET]". A data reference must provide the database name, accession number/identifiers and a resolvable link to the landing page from which the data can be accessed at the end of the reference. Further instructions are available at .

10) We replaced Supplementary Information with Expanded View (EV) Figures and Tables that are collapsible/expandable online. A maximum of 5 EV Figures can be typeset. EV Figures should be cited as 'Figure EV1, Figure EV2" etc... in the text and their respective legends should be included in the main text after the legends of regular figures.

- For the figures that you do NOT wish to display as Expanded View figures, they should be bundled together with their legends in a single PDF file called *Appendix*, which should start with a short Table of Content. Appendix figures should be referred to in the main text as: "Appendix Figure S1, Appendix Figure S2" etc.

- Additional Tables/Datasets should be labeled and referred to as Table EV1, Dataset EV1, etc. Legends have to be provided in a separate tab in case of .xls files. Alternatively, the legend can be supplied as a separate text file (README) and zipped together with the Table/Dataset file.

See detailed instructions here:

.

11) The paper explained: EMBO Molecular Medicine articles are accompanied by a summary of the articles to emphasize the major findings in the paper and their medical implications for the non-specialist reader. Please provide a draft summary of your article highlighting

- the medical issue you are addressing,

- the results obtained and

- their clinical impact.

This may be edited to ensure that readers understand the significance and context of the research. Please refer to any of our published articles for an example.

12) Author contributions: You will be asked to provide CRediT (Contributor Role Taxonomy) terms in the submission system. These replace a narrative author contribution section in the manuscript.

13) A Conflict of Interest statement should be provided in the main text.

14) Every published paper now includes a 'Synopsis' to further enhance discoverability. Synopses are displayed on the journal

webpage and are freely accessible to all readers. They include a short stand first (maximum of 300 characters, including space) as well as 2-5 one-sentences bullet points that summarizes the paper. Please write the bullet points to summarize the key NEW findings. They should be designed to be complementary to the abstract - i.e. not repeat the same text. We encourage inclusion of key acronyms and quantitative information (maximum of 30 words / bullet point). Please use the passive voice. Please attach these in a separate file or send them by email, we will incorporate them accordingly.

Please also suggest a striking image or visual abstract to illustrate your article as a PNG file 550 px wide x 300-600 px high.

15) Include a Reagents and Tools Table as part of the Methods section, which can be downloaded from our author guidelines (https://www.embopress.org/page/journal/17574684/authorguide#structuredmethods)

EMBO Molecular Medicine has a "scooping protection" policy, whereby similar findings that are published by others during review or revision are not a criterion for rejection. Should you decide to submit a revised version, I do ask that you get in touch after three months if you have not completed it, to update us on the status.

***** Reviewer's comments *****

Referee #1 (Comments on Novelty/Model System for Author):

Li et al. delivered a structured exploration into the therapeutic promise of a novel haptoglobin β (Hpβ) fusion protein, designed to neutralize circulating myoglobin in the context of rhabdomyolysis-induced acute kidney injury (RM-AKI). The study demonstrates that native haptoglobin effectively engages excess myoglobin in a murine RM-AKI model, reinforcing its physiological relevance. This mechanistic insight is further illuminated through metabolic imaging studies in RM-AKI mice, offering a dynamic visualization biodistribution and functional engagement of the fusion protein. Overall, the manuscript is well-organized, presenting a targeted, mechanism-driven strategy that addresses a pressing unmet need in clinical nephrology. The development of this fusion protein as a precision therapeutic constitutes a significant contribution to the field. I only have a few minor suggestions.

Specific comments
1. The authors demonstrated that the Mb-GST-Hpβ protein complex are colocalized with CD163 on the surface of RAW264.7 cells via immunocytostaining in Fig. 4E. Specifically, the authors are encouraged to validate CD163-dependent interaction through complementary techniques such as co-immunoprecipitation or Western blot analysis.

2. While tubular damage represents a well-established pathological feature of rhabdomyolysis-induced acute kidney injury, the contribution of endothelial injury merits further investigation. Current evidence indicates that myoglobin and its heme moiety promote endothelial dysfunction through oxidative stress generation and nitric oxide scavenging, leading to impaired vasodilation. The potential exacerbation of systemic endothelial toxicity by recirculating myoglobin immune complexes remains to be elucidated. To address this question, the authors are encouraged to evaluate markers of endothelial activation, such as P-selectin or Vcam-1 across different organ systems. qPCR analysis would provide initial insights. Such investigation would significantly enhance our understanding of the systemic endothelial pathology in rhabdomyolysis.

3. In Fig. 2L, the surface plasmon resonance (SPR) experiments provide valuable kinetic data; however, the absence of information regarding ionic strength conditions limits the interpretability and reproducibility of these findings. The authors are requested to specify the buffer composition and salt concentration used in the SPR assays.

4. Statistical analysis of the immunoblotting protein bands in Fig. 1N should be performed.

5. The study lacks any assessment of the safety or toxicity of the GST-Hpβ fusion protein. Basic histopathological examination of key organs (especially liver and spleen, given the proposed CD163-mediated clearance) is necessary to rule out off-target effects or tissue damage.

Referee #2 (Comments on Novelty/Model System for Author):

The overall technical quality of the study is high. The authors employed well-controlled in vivo and in vitro experiments with appropriate statistical analyses and reproducible datasets. The figures are clear, and the methodology is sound.

The novelty is also high. The design of a recombinant haptoglobin β fusion protein (GST-Hpβ) to neutralize circulating myoglobin

represents a creative and mechanistically distinct approach compared to traditional antibody-based or small-molecule interventions.

The medical impact is medium. While the findings are promising and clinically relevant to rhabdomyolysis-associated acute kidney injury (RM-AKI), additional pharmacokinetic and safety studies are required before clinical translation can be envisioned.

The model system is adequate. The glycerol-induced RM-AKI mouse model is widely recognized and appropriate for studying myoglobin-mediated renal injury.

Referee #2 (Remarks for Author):

This manuscript presents an original investigation into a recombinant haptoglobin β fusion protein (GST-Hpβ) designed to bind circulating myoglobin and thereby attenuate rhabdomyolysis-induced acute kidney injury (RM-AKI). The concept is mechanistically sound and translationally relevant, addressing a critical unmet need in RM-AKI therapy where no specific drugs currently exist. The study is well organized, and the results are clearly presented. However, several aspects concerning mechanistic validation, experimental rigor, and discussion depth require further clarification to enhance the manuscript's scientific robustness and overall impact.

1.The authors propose that CD163-mediated macrophage phagocytosis is the primary mechanism responsible for GST-Hpβ-myoglobin complex clearance. This conclusion, however, is supported only by colocalization and DYN inhibition data. To establish causality, additional experiments such as CD163 knockdown or blocking antibody treatment should be performed. Without these data, the mechanistic interpretation remains largely correlative rather than causative.

2.The manuscript suggests that the GST tag might confer antioxidant or detoxifying effects, but this point is not experimentally demonstrated. Although a GST-only control group is included, the Discussion does not clarify whether GST alone exerts any biological effect. The authors should either present supporting data or explicitly acknowledge this limitation to prevent overinterpretation.

3.While the in vitro stability of the protein at 25{degree sign}C for seven days is shown, no in vivo pharmacokinetic or toxicity evaluation is provided. To support the therapeutic claims, at least preliminary information on the circulating half-life, tissue distribution, and potential immunogenicity of the fusion protein should be included or discussed.

4.The study appears to build on the authors' previous Nature Communications (2025;16:1044) paper involving anti-myoglobin monoclonal antibodies. The Introduction should more explicitly differentiate this work by highlighting the conceptual and mechanistic innovations of the GST-Hpβ design-particularly its reliance on an endogenous scavenger mechanism and CD163-mediated clearance rather than antibody-based neutralization.

5.Both intravenous (i.v.) and intraperitoneal (i.p.) routes were tested, but subsequent experiments used only the i.p. route. The Abstract should therefore be revised to "intraperitoneal administration" to accurately reflect the main experimental design.

6.Since rhabdomyolysis has diverse etiologies, including drug toxicity, alcohol abuse, and extreme physical exertion, the Discussion should be expanded to include these non-traumatic causes to enhance the clinical relevance of the findings. Moreover, the potential toxicity and immunogenicity of recombinant proteins should be discussed, especially in the context of their suitability for repeated or emergency administration.

7.Several figures would benefit from clearer annotation and quantification. In Figure 1P, the anatomical localization of glomeruli and tubulointerstitial regions should be clearly indicated. In Figure 4D-E, the inclusion of quantitative intensity plots comparing DYN-treated and control groups would better support the conclusions. In Figure 6, quantitative fluorescence intensity decay curves or equivalent analysis should accompany the imaging data to strengthen interpretation.

8.A number of linguistic and technical issues should be corrected for precision and professionalism. The phrase "the therapeutic effect was insignificant in both i.p. and i.v." should read "the therapeutic effect was comparable between i.p. and i.v. routes." The sentence "the molecular weight of GST-Hpβ-Mb complexes (72.8 kDa) over the 69 kDa of glomerular filtration barrier" should be revised for grammatical accuracy, for example, "The GST-Hpβ-Mb complex (72.8 kDa) exceeds the molecular size threshold (~69 kDa) of the glomerular filtration barrier." The term "the golden standard" should be changed to "the gold standard." All abbreviations, such as KIM-1 and NGAL, should be fully spelled out at first mention. Additionally, overuse of intensifiers like "remarkably" or "strikingly" should be avoided and replaced with "significantly" for a more balanced tone.

Referee #3 (Comments on Novelty/Model System for Author):

Figure 1.
Human model
1. The choice of the clinical rhabdomyolysis model (sepsis) is questionable because it is complex, whereas it would have been better to study cases of crush syndrome or polytrauma. There are many causes of acute kidney injury during sepsis, and here, the average CK values observed suggest that rhabdomyolysis is moderate and perhaps too low to generate tubular Mb precipitation.
2. If haptoglobin binds myoglobin, its concentration should be reduced, as is the case during intravascular haemolysis when it binds to haemoglobin. During sepsis, the systemic inflammatory response is such that haptoglobin may be induced.
Murine model
1. myoglobin appears to be in the tubular cells, but not in the lumens, which indicates reabsorption. Conventional histology and IHC staining for myoglobin should be performed to better assess the lesion profile and identify casts.
2. It is surprising that the replicates in Fig. 1 O, Q and R show no variance.
Figure 3.
Treatment of mice with GST-Hpb: theoretically, the product should be injected at the time of diagnosis of rhabdomyolysis, as this is how the treatment will be prescribed in patients, i.e. when CPK levels rise above a critical threshold and before the onset of ARF.
The saline and treatment groups are not comparable, as the saline group receives lower volumes than the treatment group. Since hydration and alkalisation are the standard treatments, a hyperhydration/alkalisation arm is needed.
A histological study with IHC staining for myoglobin would be required.
Here again, there is virtually no variability between the six replicates for the study of renal function.
Figure 6.
Finally, GST-Hpb is eliminated via the urinary tract (fluorescence is found in the bladder). There is no fixation in the spleen, as might be expected?

Zeljko Durdevic, PhD

Senior Scientific Editor, *EMBO Molecular Medicine*

Dear Zeljko,

We sincerely appreciate the time and expertise you and the reviewers have dedicated to evaluating our manuscript entitled **"Engineered Haptoglobin β Fusion Protein Targets Myoglobin and Ameliorates Rhabdomyolysis-Associated Acute Kidney Injury"** (EMM-2025-22760-T). We have carefully addressed all comments raised during the review process and submitted a revised manuscript for your reference. In addition, we have prepared a detailed point-by-point response document, in which reviewer comments are presented in black and our responses in red.

In the revised manuscript, all modifications have been clearly marked in track changes. We have also submitted all required source data files and completed editorial checklists as requested. In addition, all corresponding authors have now registered their ORCID identifiers in the submission system: Yanhua Gong (0000-0002-2693-0498) and Shuquan Rao (0000-0003-4281-2392).

In recognition of substantial contributions during the revision phase, we have formally updated the author list in accordance with established authorship criteria, ensuring accurate reflection of each individual's scientific contribution. The revised authorship has been reviewed and approved by all co-authors.

Once again, we thank you for your valuable guidance throughout the review process. We believe the revised manuscript has been substantially improved and hope it meets the high standards of ***EMBO Molecular Medicine***.

Sincerely,

Yanhua Gong and co-authors

E-mail: gongyanhua@tju.edu.cn

Point-by-point responses

Referee #1 (Comments on Novelty/Model System for Author):

Li et al. delivered a structured exploration into the therapeutic promise of a novel haptoglobin β (Hpβ) fusion protein, designed to neutralize circulating myoglobin in the context of rhabdomyolysis-induced acute kidney injury (RM-AKI). The study demonstrates that native haptoglobin effectively engages excess myoglobin in a murine RM-AKI model, reinforcing its physiological relevance. This mechanistic insight is further illuminated through metabolic imaging studies in RM-AKI mice, offering a dynamic visualization biodistribution and functional engagement of the fusion protein. Overall, the manuscript is well-organized, presenting a targeted, mechanism-driven strategy that addresses a pressing unmet need in clinical nephrology. The development of this fusion protein as a precision therapeutic constitutes a significant contribution to the field. I only have a few minor suggestions.

Response: We thank the reviewer for carefully reviewing our manuscript and giving positive comments. Your constructive suggestion improved the quality of our manuscript, making it clear and transparent.

Specific comments

1. The authors demonstrated that the Mb-GST-Hpβ protein complex are colocalized with CD163 on the surface of RAW264.7 cells via immunocytostaining in Fig. 4E. Specifically, the authors are encouraged to validate CD163-dependent interaction through complementary techniques such as co-immunoprecipitation or Western blot analysis.

Response: Thank you for the constructive suggestions that strengthened our manuscript and made it more rigorous and comprehensive. Firstly, we incubated GST-Hpβ and Mb at 37°C for 0.5 h, then added them to RAW264.7 cells induced with 20 ng/mL IL-4 for 48 h to increase CD163 expression. After 0.5 h, the cells were collected to extract total protein and incubated with anti-CD163 antibody-Protein A magnetic beads. In samples bound to the anti-CD163 antibody, Western blot analysis detects the level of His-Mb and GST-Hpβ proteins, demonstrating an interaction between CD163 and the GST-Hpβ-Mb protein complex (**Fig. 4F**). Moreover, the GST

pull-down yielded consistent results (**Fig. 4G**). To further demonstrate that the recognition of the GST-Hpβ-Mb protein complex by macrophages is mediated through the CD163 receptor. We construct *Cd163*-knockout (KO) RAW264.7 cell lines or use an anti-CD163-blocking antibody to block CD163 function (**Fig. 4H-I**). In addition, the CD163 receptor is calcium- and pH-dependent, chelating Ca^{2+} with EDTA or lowering pH to 6.0 can both disrupt the binding of CD163 to GST-Hpβ-Mb protein complex **(Fig. 4H-I)**. Added above experiment results suggested that the surface receptors CD163 on RAW 264.7 cells can recognize the GST-Hpβ-Mb protein complex.

We added the above new results in the "Results" section as follows:

"In addition, CD163 on macrophages is a high-affinity natural receptor for the Hp-Hb complex. Does the recognition of the GST-Hpβ-Mb protein complex by macrophages also occur through the CD163 receptor? Immunofluorescence results showed that the GST-Hpβ-Mb protein complex colocalized with CD163 on the surface of RAW 264.7 cells **(Fig. 4E)**. Co-IP or GST-pulldown results indicated the interaction between CD163 and the GST-Hpβ-Mb protein complex **(Fig. 4F-G)**. Moreover, knocking out CD163 in macrophages or blocking its function with an anti-CD163 antibody was shown to interfere with CD163 binding to the GST-Hpβ-Mb protein complex **(Fig. 4H-I)**. Since the CD163 receptor is calcium- and pH-dependent, chelating Ca^{2+} with EGTA or lowering the pH to 6.0 can both disrupt the binding of CD163 to the GST-Hpβ-Mb protein complex **(Fig. 4H-I)**. These results suggested that the surface receptor CD163 on RAW 264.7 cells can recogize the GST-Hpβ fusion protein and perform receptor-mediated phagocytosis of Mb."

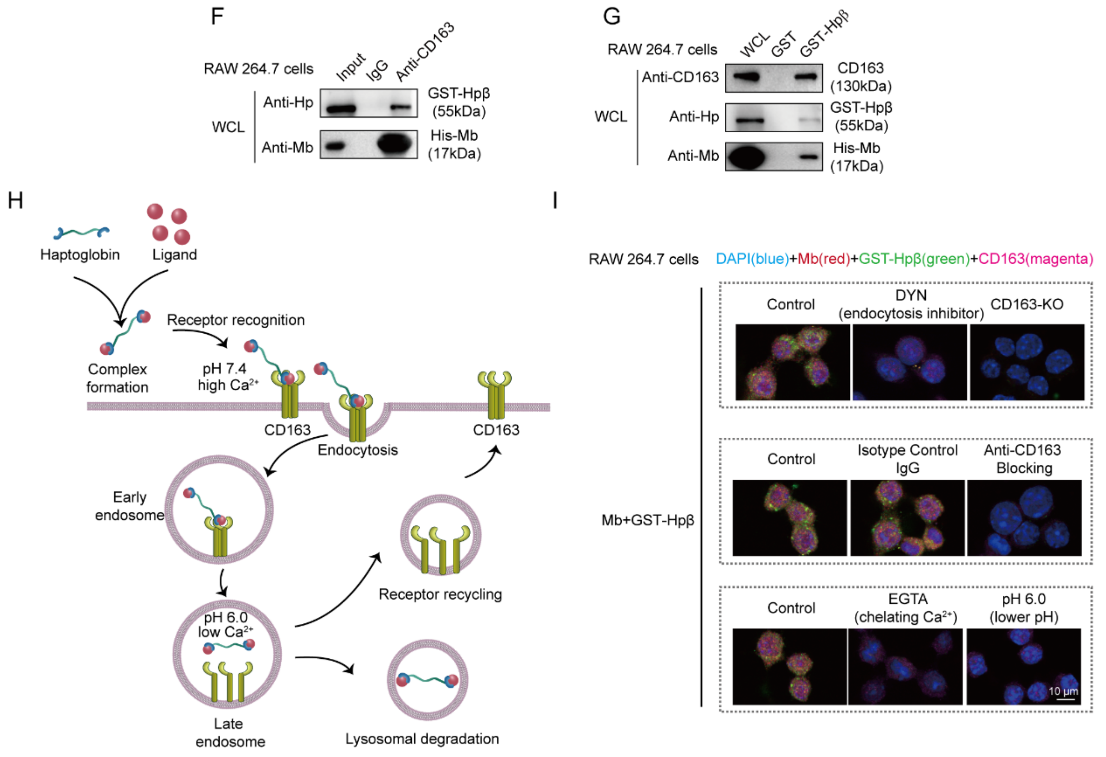

Fig. 4 Recombinant Hpβ fusion protein prevents free Mb from entering TECs and guides Mb to macrophages

(F) RAW 264.7 cells treated with 20 ng/mL IL-4 for 48 h to increase CD163 expression, then incubated with GST-Hpβ-Mb protein complex (GST-Hpβ, 100 μg/mL; His-Mb, 37.5μg/mL; molar ratio= 1:1) for 0.5 h. RAW 264.7 cell lysate was incubated with anti-CD163 antibody-Protein A magnetic beads. Represented Co-IP results of GST-Hpβ, His-Mb of the above treatment. IP with IgG control antibody (Solarbio, #SP031), anti-CD163 antibody (Santa.Cruz, #sc-58965), anti-Mb antibody (Abcam, #ab77232), or anti-Hp antibody (HUABIO, #ET1703-24). WCL, whole cell lysate.

(**G**) GST pulldown assays analysis of the interaction between GST-Hpβ-Mb and CD163 from IL-4-treated RAW 264.7 cells. WCL, whole cell lysate.

(**H**) Mechanism of CD163-mediated endocytosis of Hp complex. On the surface of macrophages, CD163 forms calcium- and pH-dependent oligomers that bind to the Hp-ligand complex. After phagocytosis, the low Ca^{2+} concentration and low pH in late lysosomes cause CD163 to dissociate, releasing the Hp-ligand complex. Monomeric CD163 returns to the cell membrane, while the Hp-ligand complex is degraded in the lysosome.

(I) Representative confocal microscopy images of RAW 264.7 cells treated with GST-Hpβ-Mb protein complex (37.5μg ferrous His-Mb (red), 100 μg/mL GST-Hpβ fusion protein (green)) in the presence or absence of human IgG1 isotype control (Selleck, Cat# A2501, 10 μg/mL), anti-CD163 antibody (Selleck, Cat# A2565, 10 μg/mL), EGTA (2 mM), pH 6.0, or macrophage membrane dynamic protein inhibitor DYN (80 μM) for 0.5 h stained for CD163 (purple). The CRISPR/Cas9 technique was used to generate *Cd163*-knockout (KO) RAW264.7 cell lines.

2. While tubular damage represents a well-established pathological feature of rhabdomyolysis-induced acute kidney injury, the contribution of endothelial injury merits further investigation. Current evidence indicates that myoglobin and its heme moiety promote endothelial dysfunction through oxidative stress generation and nitric oxide scavenging, leading to impaired vasodilation. The potential exacerbation of systemic endothelial toxicity by recirculating myoglobin immune complexes remains to be elucidated. To address this question, the authors are encouraged to evaluate markers of endothelial activation, such as P-selectin or Vcam-1 across different organ systems. qPCR analysis would provide initial insights. Such investigation would significantly enhance our understanding of the systemic endothelial pathology in rhabdomyolysis.

Response: Thanks for your constructive suggestion. As the reviewer said, evaluating endothelial injury is important to enhance our understanding of the systemic endothelial pathology in rhabdomyolysis. Therefore, we investigated whether GST-Hpβ-Mb protein immune complexes exacerbate systemic endothelial toxicity. The qPCR results showed that endothelial activation marker *Vcam-1* expression in the GST-Hpβ group was reduced in both affected organs (muscle, kidney) and other organs (spleen, lung, heart,) in 24 h compared with the Gly group and Gly+GST group (**Fig. 3N-S**). The GST-Hpβ-Mb immune complexes do not cause systemic endothelial toxicity. We added the above results in the "Results" section as follows:

"Meanwhile, qPCR results showed that endothelial activation marker *Vcam-1* mRNA expression in the Gly+GST-Hpβ group was reduced in both affected organs (kidney, muscle) and other organs (spleen, heart, lung) compared with the Gly and Gly+GST group (**Fig. 3N-S**)."

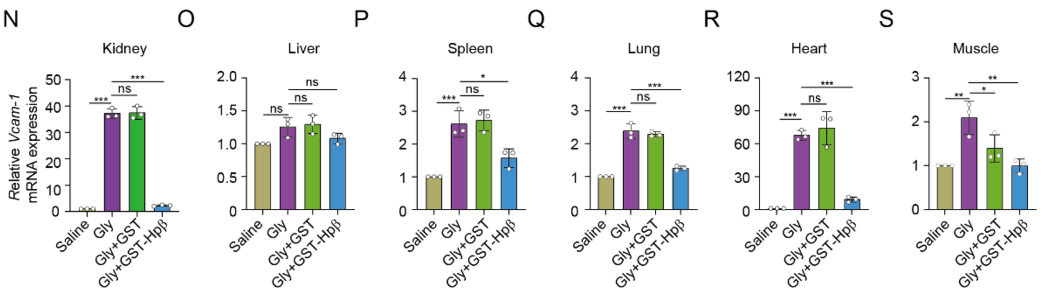

Fig. 3 Recombinant Hpβ fusion protein injection alleviates RM-AKI *in vivo*

(N-S) Endothelial activation marker *Vcam-1* mRNA expression in the affected organs (kidney, (**N**), muscle (**S**)) and other organs (liver (**P**), spleen (**Q**), lung (**R**), and heart (**R**)) of the selected groups of mice at 24h.

3. In Fig. 2L, the surface plasmon resonance (SPR) experiments provide valuable kinetic data; however, the absence of information regarding ionic strength conditions limits the interpretability and reproducibility of these findings. The authors are requested to specify the buffer composition and salt concentration used in the SPR assays.

Response: We are grateful for your rigorous attitude and constructive suggestions. We added the ionic strength used for the SPR experiments in the caption of Fig. 2L and Methods of Surface Plasmon Resonance (SPR) as follows:

In the caption of Fig. 2L:

"**(L)** SPR sensorgrams of GST-Hpβ to the recombinant mouse His-Mb immobilized sensor chip. The raw data is shown in blue lines, and the calculated fit is shown in red. The running buffer used for ligand attachment and analyte-binding experiments was 0.01 M PBS containing 2 mM KH_2PO_4, 8 mM $Na_2HPO_4 \cdot 12H_2O$, 136 mM NaCl, and 2.6 mM KCl, adjusted to pH 7.4."

In the Methods of Surface Plasmon Resonance (SPR):

"The affinity between GST-Hpβ fusion protein and His-Mb was measured using a WeSPR^{TM} 200 SPR instrument (XLement). All proteins were exchanged into the running buffer, 0.01 M PBS (2 mM KH_2PO_4, 8 mM $Na_2HPO_4 \cdot 12H_2O$, 136 mM NaCl, 2.6 mM KCl, adjusted to pH 7.4)."

4. Statistical analysis of the immunoblotting protein bands in Fig. 1N should be performed.

Response: Thank you for the good suggestion. The grey values of representative protein bands from Western blots, along with the statistical analysis results, are shown in Figure EV1H-J.

Fig.1N

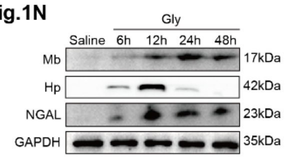

Fig. EV1

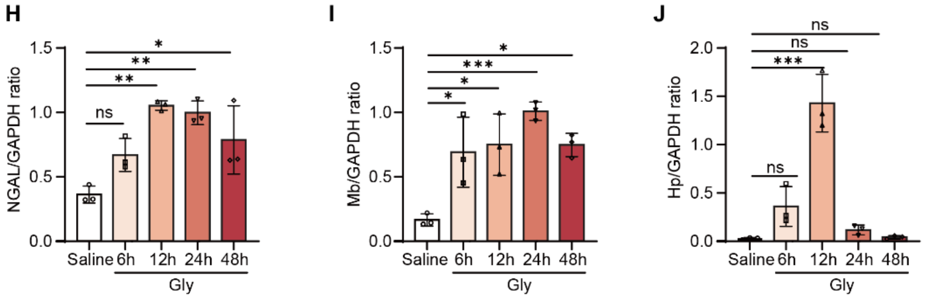

Figure EV1. Association between Mb and Hp in disease

(H-J) The grey values of the protein levels of NGAL bands (**H**), Mb bands (**I**), and Hp bands (**J**) from Western blot (**Fig. 1N**) were employed for statistical analysis (n = 3 for biological replicates).

For statistical analysis, the One-Way ANOVA (**B-L**) was used. Data are expressed as mean ± SD. $^*p < 0.05$, $^{**} p < 0.01$, $^{***} p < 0.001$, ns: not significant. Source data for this figure are available online.

5. The study lacks any assessment of the safety or toxicity of the GST-Hpβ fusion protein. Basic histopathological examination of key organs (especially liver and spleen, given the proposed CD163-mediated clearance) is necessary to rule out off-target effects or tissue damage.

Response: We thank the reviewer for pointing out this problem. As you mentioned, assessing of the safety or toxicity of the GST-Hpβ fusion protein in affected organs and other organs is important. Therefore, we increased the pathological test results of affected organs (muscle, kidney) and other organs (liver, spleen, lung, and heart) after 24 h and 7 d of recombinant GST-Hpβ fusion protein injection (**Fig. EV3A-B**). The results showed no obvious toxic side effects following injection of the GST-Hpβ fusion protein. We added the above results in the "Results" section as follows:

"Moreover, compared to the NC group, the other three groups showed muscle damage, demonstrating the successful construction of the RM model (**Fig. EV3A-B**). Compared with the Gly group, the Gly+GST-Hpβ group showed reduced pathological damage in the affected organ kidney, and no significant changes were observed in the

liver, spleen, heart, and lung at 24 h or 7 d (**Fig. EV3A-B**). These results indicated that early-stage GST-Hpβ fusion protein treatment improves kidney function, reduces kidney injury, and does not cause apparent toxic adverse effects on vital organs within 7 days of injection."

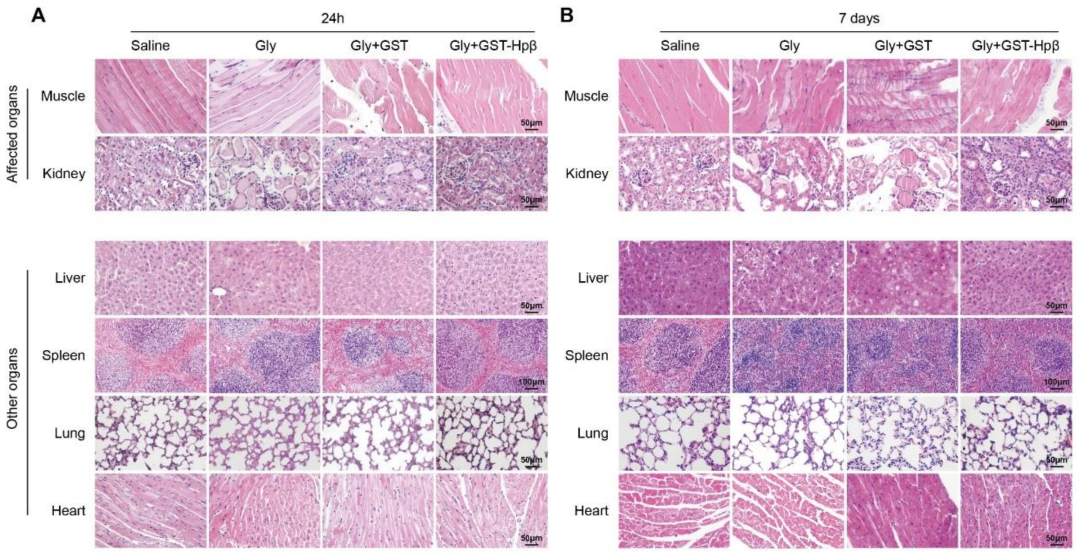

Figure EV3. Pathological examination of major organs in RM-AKI mice treated with GST-Hpβ fusion protein.

(**A-B**) HE staining was used to analyze the injury in the affected organs (muscle, kidney) and other organs (liver, spleen, lung, and heart) in RM-AKI model mice after injection of recombinant GST-Hpβ fusion protein for 24 h or 7 d.

Referee #2 (Comments on Novelty/Model System for Author):

The overall technical quality of the study is high. The authors employed well-controlled in vivo and in vitro experiments with appropriate statistical analyses and reproducible datasets. The figures are clear, and the methodology is sound.

Response: We thank the reviewer for the positive evaluation of our manuscript and for the valuable feedback, which prompted us to be more accurate and to clarify the manuscript.

The novelty is also high. The design of a recombinant haptoglobin β fusion protein (GST-Hpβ) to neutralize circulating myoglobin represents a creative and mechanistically distinct approach compared to traditional antibody-based or small-molecule interventions.

Response: We appreciate the reviewers' high praise for the novelty of this manuscript. This is indeed different from traditional methods for neutralizing circulating Mb, such as antibody or small-molecule interventions. The original idea was also to identify proteins in our body that can bind to Mb, forming large molecular-weight complexes over the glomerular filtration barrier (GFB, ~ 69 kDa).

The medical impact is medium. While the findings are promising and clinically relevant to rhabdomyolysis-associated acute kidney injury (RM-AKI), additional pharmacokinetic and safety studies are required before clinical translation can be envisioned.

Response: We thank the reviewer for their positive evaluation of the manuscript and for their valuable comments on how to improve it further. The primary purpose of this study is to conduct a proof of concept to demonstrate that the strategy of binding Hpβ to Mb is feasible. As the reviewer mentioned, applying GST-Hpβ or its analogs in clinical translation still requires pharmacokinetic and safety evaluations, which we will discuss as a limitation in the "Discussion section" as follows (underline highlight):

"Thirdly, the tandem multi-beta-subunit of Hp has a large molecular weight, binds more free Mb, and may have a more significant treatment effect than the GST-Hpβ fusion protein in RM-AKI. In fact, this research only completed the proof of concept for

treating RM-AKI with the Hpβ fusion protein. Before clinical translation, additional pharmacokinetic and safety studies are required, including a thorough assessment of long-term toxicity and immune responses."

In addition, we have added some toxic adverse effects evaluation, such as the endothelial activation marker *Vcam-1* and pathological examinations of major organs, as follows:

"Meanwhile, qPCR results showed that endothelial activation marker *Vcam-1* mRNA expression in the Gly+GST-Hpβ group was reduced in both affected organs (kidney, muscle) and other organs (spleen, heart, lung) compared with the Gly and Gly+GST group (**Fig. 3N-S**). The GST-Hpβ-Mb complexes do not cause systemic endothelial toxicity. Moreover, compared to the NC group, the other three groups showed muscle damage, demonstrating the successful construction of the RM model (**Fig. EV3A-B**). Compared with the Gly group, the Gly+GST-Hpβ group showed reduced pathological damage in the affected organ kidney, and no significant changes were observed in the liver, spleen, heart, and lung at 24 h or 7 d (**Fig. EV3A-B**). These results indicated that early-stage GST-Hpβ fusion protein treatment improves kidney function, reduces kidney injury, and does not cause apparent toxic adverse effects on vital organs within 7 days of injection."

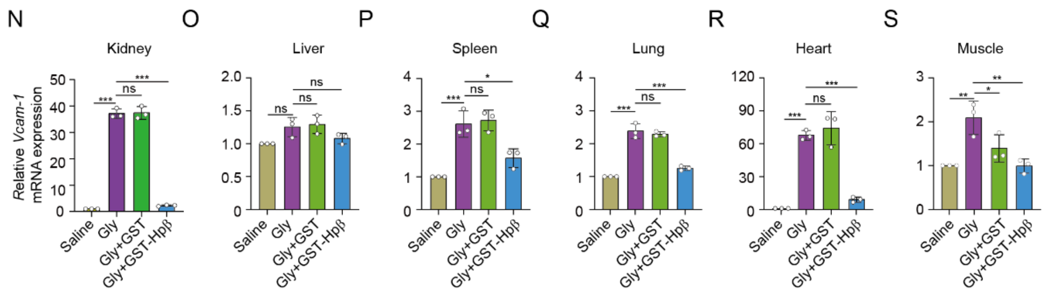

Fig. 3 Recombinant Hpβ fusion protein injection alleviates RM-AKI *in vivo*

(N-S) Endothelial activation marker *Vcam-1* mRNA expression in the affected organs (kidney, (**N**), muscle (**S**)) and other organs (liver (**P**), spleen (**Q**), lung (**R**), and heart (**R**)) of the selected groups of mice at 24h.

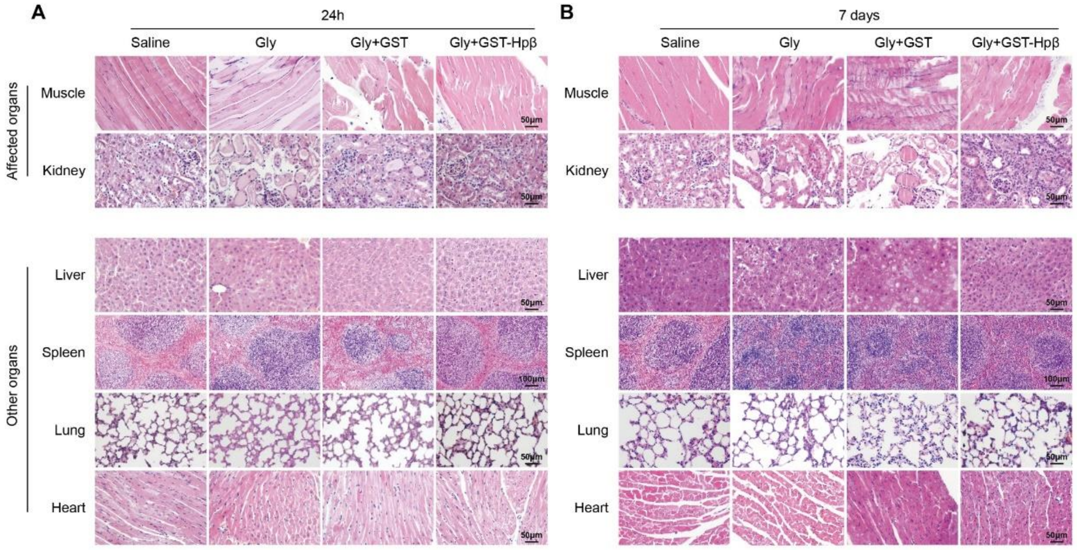

Figure EV3. Pathological examination of major organs in RM-AKI mice treated with GST-Hpβ fusion protein.

(**A-B**) HE staining was used to analyze the injury in the affected organs (muscle, kidney) and other organs (liver, spleen, lung, and heart) in RM-AKI model mice after injection of recombinant GST-Hpβ fusion protein for 24 h or 7 d.

The model system is adequate. The glycerol-induced RM-AKI mouse model is widely recognized and appropriate for studying myoglobin-mediated renal injury.

Response: We extend our gratitude to the reviewer for dedicating time and effort to provide us with insightful feedback.

Referee #2 (Remarks for Author):

This manuscript presents an original investigation into a recombinant haptoglobin β fusion protein (GST-Hpβ) designed to bind circulating myoglobin and thereby attenuate rhabdomyolysis-induced acute kidney injury (RM-AKI). The concept is mechanistically sound and translationally relevant, addressing a critical unmet need in RM-AKI therapy where no specific drugs currently exist. The study is well organized, and the results are clearly presented. However, several aspects concerning mechanistic validation, experimental rigor, and discussion depth require further clarification to enhance the manuscript's scientific robustness and overall impact.

Response: Thank you very much for reviewing our manuscript and providing the

opportunity for resubmission to EMBO Molecular Medicine. Your constructive suggestion improved the quality of our manuscript, making it clear and transparent. We have carefully examined each comment and made the suggested modifications in this updated version of the manuscript.

1.The authors propose that CD163-mediated macrophage phagocytosis is the primary mechanism responsible for GST-Hpβ-myoglobin complex clearance. This conclusion, however, is supported only by colocalization and DYN inhibition data. To establish causality, additional experiments such as CD163 knockdown or blocking antibody treatment should be performed. Without these data, the mechanistic interpretation remains largely correlative rather than causative.

Response: Thank you for the constructive suggestion that prompted us to be more accurate and clarify the manuscript. During hemolysis, in the bloodstream, hemoglobin (Hb) is bound by the acute-phase protein haptoglobin (Hp), leading to its immediate endocytosis by the cluster of differentiation 163 (CD163) macrophage receptor. CD163 is a type I transmembrane protein that belongs to the group B Scavenger Repeat Cysteine-Rich (SRCR) superfamily [1]. CD163 is composed of nine consecutive SRCR domains, followed by a transmembrane segment and a short cytoplasmic tail, which includes a functional internalization motif (Fig. A) [2]. Each SRCR domain is a compact and spherical domain of around 110 residues [3]. Xu et.al. in 2025 using Cro-EM determined that trimeric CD163 structure resembles a claw with three “toes” when viewed from the side, with the N-terminal SRCR domains capturing the Hb-Hp1 complex (Fig. B, G) [4]. The N-terminal SRCR2-4 domains of CD163 protomers directly interact with the ligand (Fig. C-G) [4]. The SRCR2 domains of CD163 protomers A and B interact with the Hp1-β subunit, while the SRCR3 and 4 domains contact the Hb subunits. Specifically, CD163 protomer A interacts with the Hb-α subunit, while CD163 protomer B interacts with the Hb-β subunit (Fig.C-G) [4]. The CD163 protomer C exhibits poor density, but it appears that the SRCR3 domain of protomer C may form direct contacts with Hp [4].

The binding of Ca^{2+} ions to CD163 is crucial not only for ligand binding but also for receptor oligomerization [5]. There are two calcium-binding sites located in SRCR2 and SRCR3, respectively (Fig. F) [4,6]. Indeed, Hp-Hb binding by CD163 is also calcium-dependent, with high-affinity binding at physiological calcium concentrations (2.5 mM $CaCl_2$) and release in low-calcium conditions [2]. Moreover, decreasing pH to 6.5 also increased dissociation of Hp-Hb from CD163, although at a slower rate [7]. This may be due to degradation of the Hp-Hb complex at endosomes. Both calcium concentration and pH are low in the late endosome. Research indicates that in cell cultures, the calcium concentration in endosomes has been measured to be less than 10 μM at pH 6.5 [7]. Under these conditions, the Hp-Hb complex may immediately dissociate from CD163. In other words, on the surface of the cell, CD163 forms Ca^{2+}-dependent oligomers that bind Hp-Hb complexes. After endocytosis, a decrease in Ca^{2+} concentration and pH in the late endosome triggers CD163 dissociation and Hp-Hb release. Monomeric CD163 is recycled back to the plasma membrane, where the CD163 trimer is reestablished, while Hp-Hb is degraded in the lysosome [5,8]. Zhou et.al indicated that no binding of CD163 with Hp-Hb was observed in the absence of calcium (EGTA to chelate calcium) or at pH 6.0 [8]. Therefore, calcium, together with pH, mediates the formation of CD163 multimers and is required for Hp-Hb uptake.

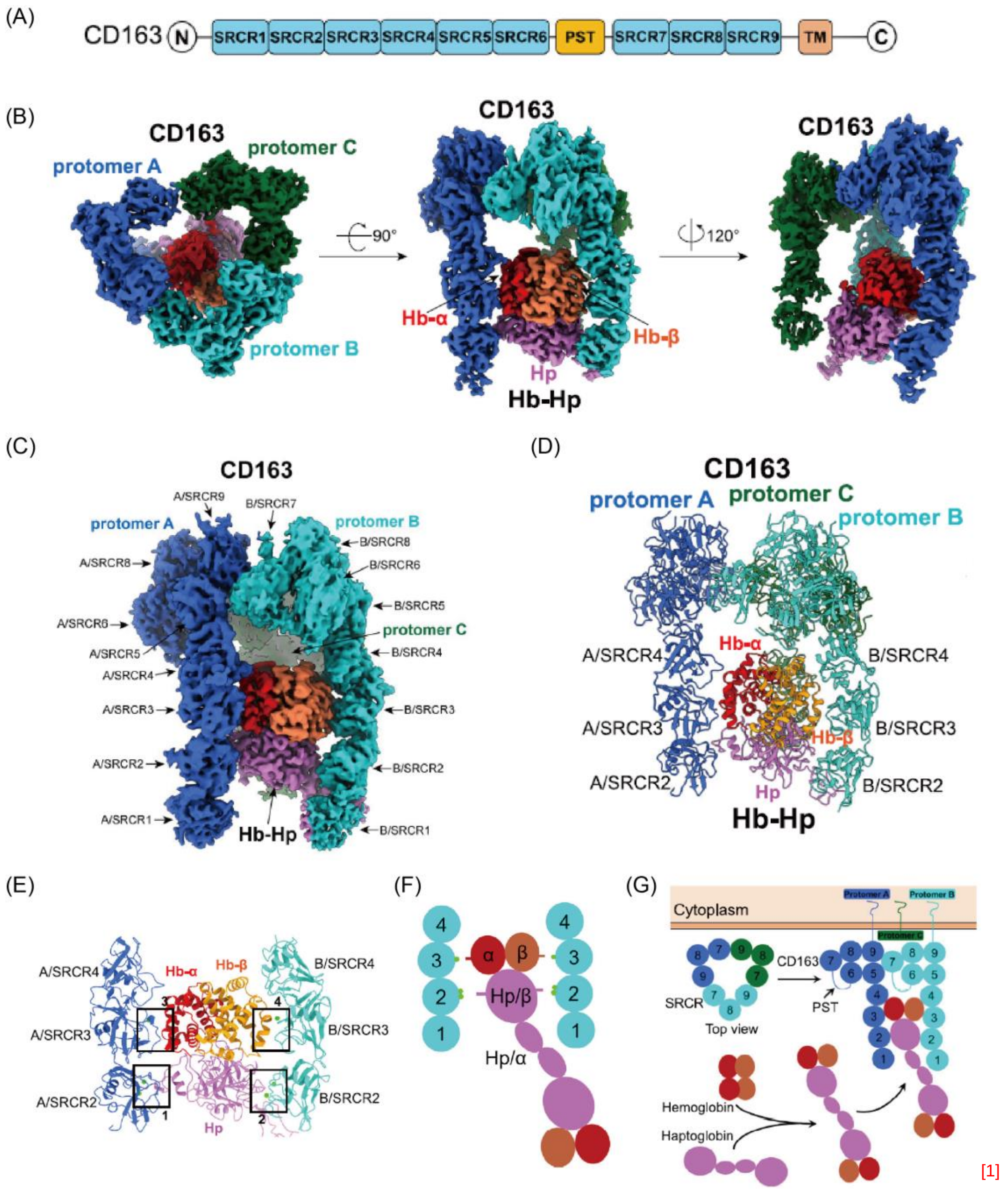

Fig. Cryo-EM structure of CD163 in complex with Hb-Hp [1].

All the data from Xu et. al.'s paper, published in Nature Communications in 2025 [1]. (A) Domain architecture of CD163. PST, proline-serine-threonine rich interdomain segment; TM, transmembrane; CCP, complement control protein; SP, serine protease. (B) Different views of the cryo-EM map of trimeric CD163 in complex with Hb-Hp. Three CD163 protomers are shown in blue, cyan, and green. Hb subunits are shown in red (α) and orange (β), Hp is shown in pink. (C-D) A Cryo-EM study showed the overall structure of CD163 in complex with Hb-Hp. (E) Structural model of Hb-Hp binding to SRCR2-4 of two CD163 protomers after local refinement. (F) Model for calcium-dependent interaction between two CD163 protomers and Hb-Hp complex. The green circle represents the calcium-binding site. (G) Model for CD163-mediated recognition and internalization of Hb-Hp complex.

Therefore, in addition to knocking out CD163 or blocking CD163 with antibodies, in order to further demonstrate that Hp-Mb clearance depends on CD163, this study also used EGTA to chelate Ca^{2+} ions and pH 6.0 to interfere with the binding of CD163 and Hp. Moreover, we added Co-IP and GST-pulldown experiments to verify the interaction between CD163 and the GST-Hpβ-Mb complex.

We added the above new results in the "Results" section as follows:

"In addition, CD163 on macrophages is a high-affinity natural receptor for the Hp-Hb complex. Does the recognition of the GST-Hpβ-Mb protein complex by macrophages also occur through the CD163 receptor? Immunofluorescence results showed that the GST-Hpβ-Mb protein complex colocalized with CD163 on the surface of RAW 264.7 cells **(Fig. 4E)**. Co-IP or GST-pulldown results indicated the interaction between CD163 and the GST-Hpβ-Mb protein complex **(Fig. 4F-G)**. Moreover, knocking out CD163 in macrophages or blocking its function with an anti-CD163 antibody was shown to interfere with CD163 binding to the GST-Hpβ-Mb protein complex **(Fig. 4H-I)**. Since the CD163 receptor is calcium- and pH-dependent, chelating Ca^{2+} with EGTA or lowering the pH to 6.0 can both disrupt the binding of CD163 to the GST-Hpβ-Mb protein complex **(Fig. 4H-I)**. These results suggested that the surface receptor CD163 on RAW 264.7 cells can recognize the GST-Hpβ fusion protein and perform receptor-mediated phagocytosis of Mb."

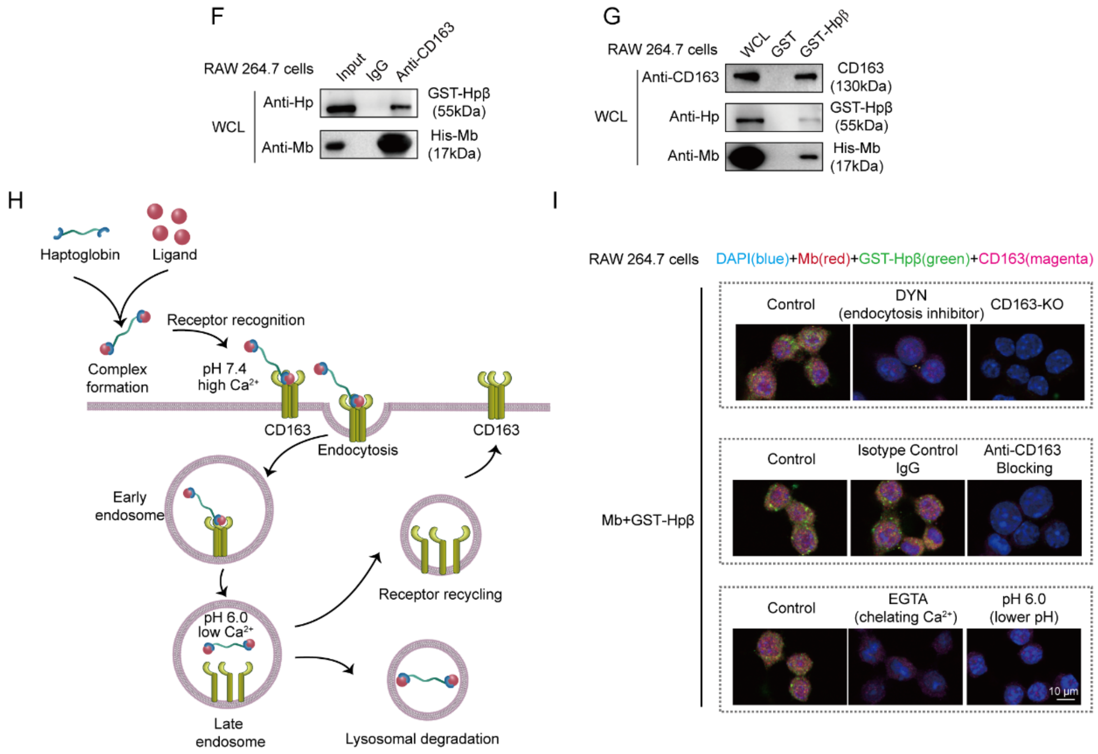

Fig. 4 Recombinant Hpβ fusion protein prevents free Mb from entering TECs and guides Mb to macrophages

(F) RAW 264.7 cells treated with 20 ng/mL IL-4 for 48 h to increase CD163 expression, then incubated with GST-Hpβ-Mb protein complex (GST-Hpβ, 100 μg/mL; His-Mb, 37.5μg/mL; molar ratio= 1:1) for 0.5 h. RAW 264.7 cell lysate was incubated with anti-CD163 antibody-Protein A magnetic beads. Represented Co-IP results of GST-Hpβ, His-Mb of the above treatment. IP with IgG control antibody (Solarbio, #SP031), anti-CD163 antibody (Santa.Cruz, #sc-58965), anti-Mb antibody (Abcam, #ab77232), or anti-Hp antibody (HUABIO, #ET1703-24). WCL, whole cell lysate.

(**G**) GST pulldown assays analysis of the interaction between GST-Hpβ-Mb and CD163 from IL-4-treated RAW 264.7 cells. WCL, whole cell lysate.

(**H**) Mechanism of CD163-mediated endocytosis of Hp complex. On the surface of macrophages, CD163 forms calcium- and pH-dependent oligomers that bind to the Hp-ligand complex. After phagocytosis, the low Ca^{2+} concentration and low pH in late lysosomes cause CD163 to dissociate, releasing the Hp-ligand complex. Monomeric CD163 returns to the cell membrane, while the Hp-ligand complex is degraded in the lysosome.

(I) Representative confocal microscopy images of RAW 264.7 cells treated with GST-Hpβ-Mb protein complex (37.5μg ferrous His-Mb (red), 100 μg/mL GST-Hpβ fusion protein (green)) in the presence or absence of human IgG1 isotype control (Selleck, Cat# A2501, 10 μg/mL), anti-CD163 antibody (Selleck, Cat# A2565, 10 μg/mL), EGTA (2 mM), pH 6.0, or macrophage membrane dynamic protein inhibitor DYN (80 μM) for 0.5 h stained for CD163 (purple). The CRISPR/Cas9 technique was used to generate *Cd163*-knockout (KO) RAW264.7 cell line.

References:

[1]. Ritter M, Buechler C, Langmann T, Schmitz G. Genomic organization and chromosomal localization of the human CD163 (M130) gene: a member of the scavenger receptor cysteine-rich superfamily. *Biochem Biophys Res Commun* 1999, 260(2)**:** 466-474.

[2] Madsen M, Moller HJ, Nielsen MJ, Jacobsen C, Graversen JH, van den Berg T, Moestrup SK. Molecular characterization of the haptoglobin.hemoglobin receptor CD163. Ligand binding properties of the scavenger receptor cysteine-rich domain region. *J Biol Chem* 2004, 279(49)**:** 51561-51567.

[3] Hohenester E, Sasaki T, Timpl R. Crystal structure of a scavenger receptor cysteine-rich domain sheds light on an ancient superfamily. *Nat Struct Biol* 1999, 6(3)**:** 228-232.

[4] Xu H, Song X, Su XD. Calcium-dependent oligomerization of scavenger receptor CD163 facilitates the endocytosis of ligands. *Nat Commun* 2025, 16(1)**:** 6679.

[5] Etzerodt A, Mikkelsen JH, Torvund-Jensen M, Hennig D, Boesen T, Graversen JH, Moestrup SK, Kollman JM, Andersen CBF. The Cryo-EM structure of human CD163 bound to haptoglobin-hemoglobin reveals molecular mechanisms of hemoglobin scavenging. *Nat Commun* 2024, 15(1)**:** 10871.

[6] Ojala JR, Pikkarainen T, Tuuttila A, Sandalova T, Tryggvason K. Crystal structure of the cysteine-rich domain of scavenger receptor MARCO reveals the presence of a basic and an acidic cluster that both contribute to ligand recognition. *J Biol Chem* 2007, 282(22)**:** 16654-16666.

[7] Gerasimenko JV, Tepikin AV, Petersen OH, Gerasimenko OV. Calcium uptake via endocytosis with rapid release from acidifying endosomes. *Curr Biol* 1998, 8(24)**:** 1335-1338.

[8] Zhou RX, Higgins MK. Scavenger receptor CD163 multimerises to allow uptake of diverse ligands. *Nat Commun* 2025, 16: 6623.

2.The manuscript suggests that the GST tag might confer antioxidant or detoxifying effects, but this point is not experimentally demonstrated. Although a GST-only control group is included, the Discussion does not clarify whether GST alone exerts any biological effect. The authors should either present supporting data or explicitly acknowledge this limitation to prevent overinterpretation.

Response: Thank you for pointing out this problem. In the Discussion section, we presented some findings from other researchers, indicating that GST in mammals has detoxifying and antioxidant functions. However, in our study, because the GST protein was derived from the Schistosoma sequence cloned into the pGEX-6P-1 vector, it did not exhibit therapeutic effects in the treatment of RM-AKI mice. The associated contents in the manuscript are as follows:

"Some studies have shown that GST exerts detoxification and antioxidant functions in eukaryotes by catalyzing the binding of electrophilic groups of harmful substances to the sulfhydryl groups of reduced glutathione (Mazari *et al*, 2023). Meanwhile, glutathione's beneficial effects on Hb-associated oxidative damage have been observed (Kirschner-Zilber *et al*, 1989). Research indicates that glutathione can act as an antioxidant to alleviate damage caused by heme proteins (Zager & Burkhart, 1998. Our research has shown that the GST tag in the GST-Hpβ fusion protein does not affect the Mb-binding function of Hpβ and the function of targeting CD163 to promote Mb clearance, which better realizes the innovative therapeutic concept of GST-Hpβ-Mb macromolecular protein complexes blocking Mb from passing through the GFB. The design of a recombinant GST-Hpβ fusion protein to neutralize circulating myoglobin represents a creative and mechanistically distinct approach compared to traditional antibody-based or small-molecule interventions. However, in this study, the prokaryotic expression of the GST tag protein has no additional independent beneficial effect in the treatment of the RM-AKI model mice, possibly because the GST fragment on the pGEX-6P-1 vector is derived from Schistosoma, and whether mammalian GST can exert antioxidant effects in mice remains to be determined. Furthermore, the xenogeneic origin of this GST-tag raises concerns about immunogenicity, an important factor for the clinical translation of any recombinant protein. While this may be a greater concern for scenarios requiring repeated administration, the risk profile is likely more acceptable for the intended single-dose emergency use in acute settings. At the very least, the Schistosoma-derived GST proteins do not negatively affect therapeutic strategies that utilize recombinant GST-Hpβ fusion proteins to alleviate RM-AKI."

In addition, we have also added the limitations of the Japanese Schistosome-derived GST used in this study in the "Our study has limitations" paragraph as follows:

"Secondly, the GST-Hpβ fusion protein is expressed as insoluble. In the prokaryotic *E. coli* expression system, GST-Hpβ fusion protein can only be purified by inclusion bodies in the precipitation. Moreover, GST derived from Schistosomes has no effect in the RM-AKI model mice, but whether mammalian-derived GST has detoxifying and antioxidant functions in treating RM-AKI needs to be investigated."

3.While the in vitro stability of the protein at 25{degree sign}C for seven days is shown, no in vivo pharmacokinetic or toxicity evaluation is provided. To support the therapeutic claims, at least preliminary information on the circulating half-life, tissue distribution, and potential immunogenicity of the fusion protein should be included or discussed.

Response: We thank the reviewer for this important point. As the reviewer mentioned, we tested the stability of the GST-Hpβ fusion protein at 25°C *in vitro*. The primary purpose of this assessment was to consider the stability of the recombinant GST-Hpβ fusion protein during storage and transportation. Additionally, in small-animal *in vivo* imaging experiments, the GST-Hpβ fusion protein was no longer detectable at the whole-mouse level after 3 days. The reviewer noted that the fusion protein's circulation half-life, tissue distribution, and potential immunogenicity are critical for the clinical translation of the drug. However, the most important finding of this study is the potential therapeutic possibility or proof of concept of using the Hpβ fusion protein to treat RM-AKI. Given the impact of GST and clinical translation, we plan to remove GST in the future while retaining Hpβ affinity and large molecular weight. For newly developed recombinant tandem multi-beta-subunit of Hp proteins in drug development, it is necessary to evaluate related factors such as drug half-life and tissue distribution.

Following the reviewers' suggestions, we have added the following content to the Discussion section:

"The GST-Hpβ fusion protein can remain stable for 3 days at 25°C *in vitro*, and it is likely to be completely degraded by 7 days (**Fig. 5A**). Small animal *in vivo* imaging experiments of mice show that the GST-Hpβ fusion protein is metabolized or degraded after 3 days (**Fig. 6B–C**). However, the therapeutic effect of the GST-Hpβ fusion protein, such as improving kidney function and reducing kidney injury, can last for 14 days (**Fig. 5D, K-N**). The clearance of Hp protein bound to ligands is generally accomplished through binding to the CD163 receptor on macrophages, leading to degradation of the Hp-ligand protein in the liver or spleen (Schaer *et al*., 2013b). In addition, since the GST-Hpβ fusion protein contains GST derived from Schistosomes, there may be immunogenicity issues after administration. Therefore, future considerations include creating a new fusion protein (a tandem multi-beta-subunit of Hp) that does not contain the GST tag while retaining the affinity of Hpβ and a large molecular weight. This optimization would directly address the immunogenicity concern while preserving the protein's core therapeutic mechanism, thereby enhancing its suitability not only for emergency use but also for potential scenarios requiring repeated administration. Moreover, for newly developed recombinant tandem multi-beta-subunit of Hp proteins in drug development, it is necessary to evaluate the circulating half-life and tissue distribution."

4.The study appears to build on the authors' previous Nature Communications (2025;16:1044) paper involving anti-myoglobin monoclonal antibodies. The Introduction should more explicitly differentiate this work by highlighting the conceptual and mechanistic innovations of the GST-Hpβ design-particularly its reliance on an endogenous scavenger mechanism and CD163-mediated clearance rather than antibody-based neutralization.

Response: We sincerely appreciate your valuable and highly insightful comments on this paper. We completely agree with your view that it is necessary to clarify more clearly and explicitly in the introduction the fundamental distinction of this study from the previously published work on anti-Mb monoclonal antibody (anti-Mb RabMAb) [PMID: 39865095] in terms of core concepts and therapeutic mechanisms. The

'endogenous scavenger mechanism' and 'CD163-mediated clearance' you pointed out are precisely the core innovations in the study's design logic. We added the following paragraph in the Introduction section:

"It is worth noting that our team recently developed a high-affinity recombinant anti-Mb rabbit monoclonal antibody targeting Mb, which blocks glomerular filtration by forming large molecular complexes, and validated the feasibility of an Mb-targeted clearance strategy in an RM-AKI model (Wang *et al*, 2025). However, this strategy relies on the neutralizing effect of exogenous antibodies and their Fc receptor-mediated clearance pathway. In stark contrast, the present study first utilizes and extends an evolutionarily conserved endogenous detoxification system (the recombinant Hp protein), which potentially offers greater safety. Secondly, it achieves targeted clearance of pathogenic Mb proteins by directing the task to macrophages with strong phagocytic capacity via the specific receptor CD163, potentially improving clearance efficiency and reducing off-target effects. Therefore, this study is not merely a repetition of previous antibody work, but represents a fundamental mechanistic innovation—shifting from 'exogenous blockade' to 'endogenous guided clearance."

Thanks again for the reviewer's precise comments, which helped us better refine and present the core value of this study. The revised introduction, following your suggestions, allows readers to immediately grasp the fundamental differences between this work and previous studies and to understand the unique advantages and innovative significance of the GST-Hpβ fusion protein as a new strategy for RM-AKI treatment based on its endogenous clearance mechanism.

5.Both intravenous (i.v.) and intraperitoneal (i.p.) routes were tested, but subsequent experiments used only the i.p. route. The Abstract should therefore be revised to "intraperitoneal administration" to accurately reflect the main experimental design.

Response: We apologize for mistakenly including the administration method in the Abstract section. We revised the "intravenous administration" to "intraperitoneal administration" in the Abstract section.

6.Since rhabdomyolysis has diverse etiologies, including drug toxicity, alcohol abuse, and extreme physical exertion, the Discussion should be expanded to include these non-traumatic causes to enhance the clinical relevance of the findings. Moreover, the potential toxicity and immunogenicity of recombinant proteins should be discussed, especially in the context of their suitability for repeated or emergency administration.

Response: Thank you very much for these two very insightful and crucial comments on the discussion section. We agree that thoroughly exploring the clinical generalizability of this study as well as the safety of the therapeutic molecules themselves is essential for a comprehensive assessment of their translational potential.

Regarding the clinical generalizability of RM rescue, we have added the following content to the Discussion section (shown with underlines):

"Rhabdomyolysis is a significant cause of AKI due to the accumulation of Mb in the kidney. The mortality rate of RM-AKI patients was reported to be as high as 60% (Li *et al*, 2022). However, at present, RM-AKI is mainly treated symptomatically, and there are no FDA-approved specific drugs. It is noteworthy that rhabdomyolysis stems from diverse etiologies, including trauma (Qiao *et al*, 2024), drug toxicity (Weston *et al*, 1986), alcohol abuse (Haller & Drachman, 1980), extreme physical exertion (Rawson *et al*, 2017), and high fiver (Luan *et al.*, 2023). While the initiating factors differ, they converge on the common pathological outcome of massive Mb release into circulation. Therefore, developing a strategy that directly targets this final common pathogenic mediator, free Mb, holds promise for broad clinical applicability across the spectrum of rhabdomyolysis causes."

Regarding the potential toxicity and immunogenicity of GST-Hpβ, we have added the following content in the Discussion section (shown with underlines):

"However, in this study, the prokaryotic expression of the GST-tag protein has no additional independent beneficial effect in the treatment of the RM-AKI model mice, possibly because the GST fragment on the pGEX-6P-1 vector is derived from

Schistosoma, and whether mammalian GST can exert antioxidant effects in mice remains to be determined. Furthermore, the xenogeneic origin of this GST tag raises concerns about immunogenicity, an important factor for the clinical translation of any recombinant protein. While this may be a greater concern for scenarios requiring repeated administration, the risk profile is likely more acceptable for the intended single-dose emergency use in acute settings. At the very least, the Schistosoma-derived GST proteins do not negatively affect therapeutic strategies that utilize recombinant GST-Hpβ fusion proteins to alleviate RM-AKI."

Furthermore, to avoid immunogenicity issues caused by the heterologous GST protein, we are considering designing a version without GST and only the Hpβ tandem in future studies. Accordingly, we have added the following content in the Discussion section (underlined):

"The GST-Hpβ fusion protein can remain stable for 3 days at 25°C *in vitro*, and it is likely to be completely degraded by 7 days (**Fig. 5A**). Small animal *in vivo* imaging experiments of mice show that the GST-Hpβ fusion protein is metabolized or degraded after 3 days (**Fig. 6B–C**). However, the therapeutic effect of the GST-Hpβ fusion protein, such as improving kidney function and reducing kidney injury, can last for 14 days (**Fig. 5D, K-N**). The clearance of Hp protein bound to ligands is generally accomplished through binding to the CD163 receptor on macrophages, leading to degradation of the Hp-ligand protein in the liver or spleen (Schaer *et al.*, 2013b). In addition, since the GST-Hpβ fusion protein contains GST derived from Schistosomes, there may be immunogenicity issues after administration. Therefore, future considerations include creating a new fusion protein (a tandem multi-beta-subunit of Hp) that does not contain the GST tag while retaining the affinity of Hpβ and a large molecular weight. This optimization would directly address the immunogenicity concern while preserving the protein's core therapeutic mechanism, thereby enhancing its suitability not only for emergency use but also for potential scenarios requiring repeated administration. Moreover, for newly developed recombinant tandem multi-beta-subunit of Hp proteins in drug development, it is necessary to evaluate the circulating half-life and tissue distribution."

7.Several figures would benefit from clearer annotation and quantification. In Figure 1P, the anatomical localization of glomeruli and tubulointerstitial regions should be clearly indicated. In Figure 4D-E, the inclusion of quantitative intensity plots comparing DYN-treated and control groups would better support the conclusions. In Figure 6, quantitative fluorescence intensity decay curves or equivalent analysis should accompany the imaging data to strengthen interpretation.

Response: We thank the reviewer for prompting us to provide clearer annotations and quantifications of the figures. Given the immunofluorescence staining images in Fig. 1P, it is difficult for readers to distinguish the anatomical localization of glomeruli and tubulointerstitial regions. Therefore, we have supplemented it with Immunohistochemical data for Mb and Hp proteins to help readers better understand the localization of Mb and Hp proteins (**Fig. 1R-T**).

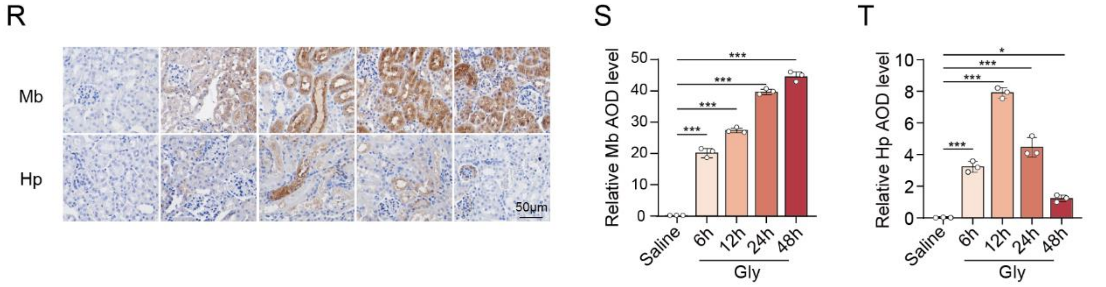

Fig. 1 Association between endogenous myoglobin and haptoglobin in human pathological conditions, and native haptoglobin binds to excess myoglobin in RM-AKI mice

(**R**) Representative immunohistochemical staining of Mb and Hp in the kidney of the Saline and Gly group mice.

(**S-T**) Quantification of Immunohistochemical data of Mb (**S**) and Hp (**T**) expression in Fig.1R (n = 3 per group for biological replicates).

For the Figure 4D, we added the quantitative intensity plots as follows:

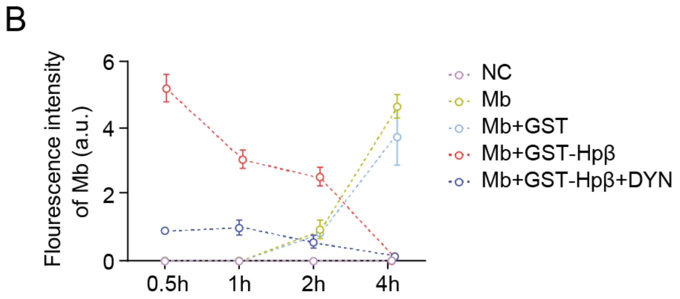

Figure EV4. Toxicity of GST-Hpβ to TCMK-1 cells and quantification of confocal microscopy images of RAW 264.7 cells metabolism of Mb

(**B**) Quantification of confocal microscopy images of RAW 264.7 cells metabolism of Mb in Fig. 4D. Quantitative analysis of the fluorescence intensity of His-Mb proteins in confocal microscopy images of RAW 264.7 cells for different times (0.5, 1, 2, 4 h). Results are presented as mean ± SD, and dots indicate individual quantitative analysis data points from 3 biological replicates.

For Figure 6, we previously statistically analyzed the fluorescence intensity decay curves for each group (**Fig. 6C**). To improve clarity, we divided the four groups into two main groups. The upper part of Fig. 6C shows that in the normal group, no fluorescence signal of the fusion protein was detected at the overall animal level after 72 hours. The lower part of Fig. 6C shows that in the model group, no fluorescence signal of the fusion protein was detected at around 72 hours. In terms of fluorescence intensity, the Gly group showed stronger fluorescence than the control group at 48 hours. Overall, RM-AKI reduces the metabolic degradation of the GST-Hpβ fusion protein in mice.

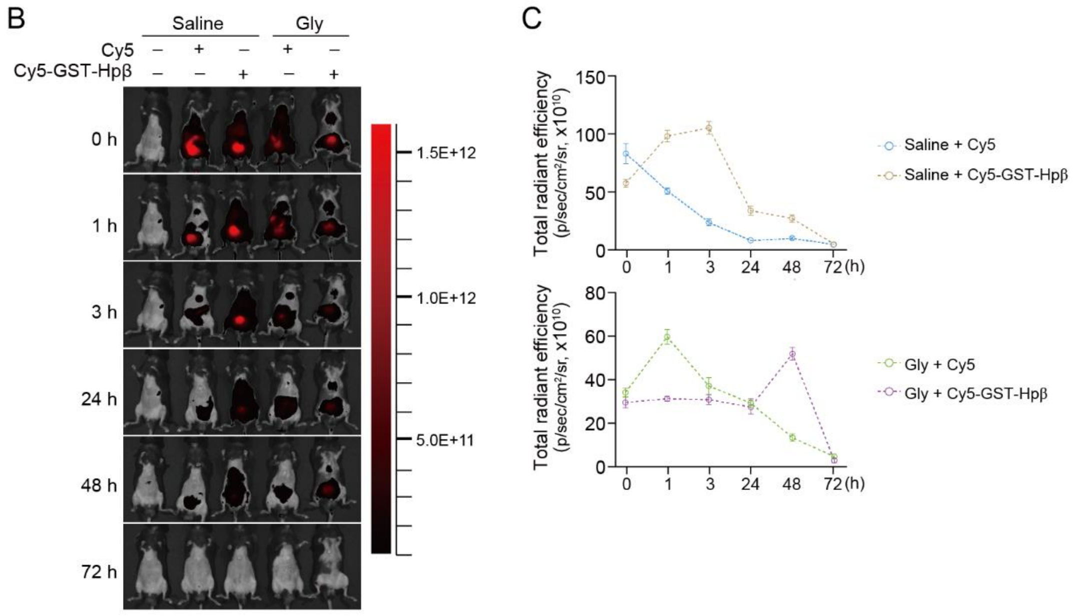

Fig. 6 Metabolic imaging of Hpβ fusion protein in RM-AKI mice

(B) *In vivo* imaging of each group of mice within 72 hours.

(C) The fluorescence intensity of each group of mice at different time points.

8.A number of linguistic and technical issues should be corrected for precision and professionalism. The phrase "the therapeutic effect was insignificant in both i.p. and i.v." should read "the therapeutic effect was comparable between i.p. and i.v. routes." The sentence "the molecular weight of GST-Hpβ-Mb complexes (72.8 kDa) over the 69 kDa of glomerular filtration barrier" should be revised for grammatical accuracy, for example, "The GST-Hpβ-Mb complex (72.8 kDa) exceeds the molecular size threshold (~69 kDa) of the glomerular filtration barrier." The term "the golden standard" should be changed to "the gold standard." All abbreviations, such as KIM-1 and NGAL, should be fully spelled out at first mention. Additionally, overuse of intensifiers like "remarkably" or "strikingly" should be avoided and replaced with "significantly" for a more balanced tone.

Response: Thank you for pointing out these problems. Firstly, we have rephrased the associated sentences in the main text. Secondly, we added the fully spelled-out terms kidney injury molecule-1 (KIM-1) and neutrophil gelatinase-associated lipocalin (NGAL) at first mention. Thirdly, we delete vocabulary like "remarkably" or "strikingly"

and replace them with "significantly".

Referee #3 (Comments on Novelty/Model System for Author):

Figure 1.

Human model

1. The choice of the clinical rhabdomyolysis model (sepsis) is questionable because it is complex, whereas it would have been better to study cases of crush syndrome or polytrauma. There are many causes of acute kidney injury during sepsis, and here, the average CK values observed suggest that rhabdomyolysis is moderate and perhaps too low to generate tubular Mb precipitation.

Response: We thank the reviewer for raising this critical point. We fully agree that ideal clinical validation would involve samples from patients with well-defined rhabdomyolysis etiology.

Since our initial purpose was to identify preliminary evidence supporting a potential association between myoglobin (Mb) and haptoglobin (Hp) across diverse pathological states, we therefore chose relevant pathological states of available human proteomics datasets (sepsis, contrast-induced acute kidney injury (CI-AKI), chronic limb-threatening ischemia (CLTI)).

To further strengthen our conclusion, we selected three independent public datasets (PXD027485, PXD024271, PXD025810), representing states of systemic inflammation (sepsis), renal injury (CI-AKI), and localized muscle ischemia (CLTI), respectively. The CLTI muscle tissue data, in particular, directly demonstrates an association between Mb release and Hp elevation in the context of ischemic muscle injury, which is closer to the core pathology of rhabdomyolysis (muscle damage). We acknowledge the multifactorial etiology of AKI in sepsis. In the serum proteomics dataset (PXD027485), we specifically analyzed a subgroup of patients with serum Mb levels above the mean (**Fig. 1A-B**), focusing on pathological states "with concomitant myoglobin elevation" to observe the Hp response trend. For the sepsis patient's blood biochemistry, we selected a case where creatine kinase (CK) levels exceeded approximately 1000 U/L (**Fig. 1I-L, Fig. EV1B-E**), as rhabdomyolysis (RM) in humans is often defined by a CK level >1000 U/L within 72 hours (as per cohort studies such as that by Finn Erland Nielsen et al.)[1]. The mean CK value for this patient appears is around 1000 U/L because the patient received immediate and continuous renal

replacement therapy (CRRT) for 48 hours upon admission. Sample collection began after CRRT was stopped; therefore, the CK concentration was declining from a peak of ~1500 U/L at that time (**Fig. 1I, Fig. EV1B**). While the normal blood Mb concentration is < 70 ng/mL, there is no absolute threshold for renal injury; however, a concentration >1000 ng/mL is generally considered to confer increased risk[2]. The patient's clinical data show that even after 48 hours of dialysis, the Mb level was only gradually decreasing from a dangerous level near 2000 ng/mL (**Fig. 1K, Fig. EV1D**).

The primary purpose of citing these data was to demonstrate the phenomenon that Hp levels frequently change under various pathological conditions involving muscle injury or elevated Mb, thereby generating the scientific hypothesis that "Hp may participate in Mb metabolism". This hypothesis was subsequently systematically validated in our controlled, classical glycerol-induced RM-AKI mouse model (Fig. 1M-Z and all subsequent experiments).

We wholeheartedly agree with the reviewer's perspective that future direct clinical validation should prioritize cohorts with conditions like crush syndrome or severe trauma. However, constrained by the difficulty of obtaining such specific clinical samples, we will address this point in the Discussion section of the revised manuscript as a recognized limitation and a key direction for future translational research. The specific addition will be as follows (underlined):

"Our study has limitations. Firstly, public datasets and sepsis patient blood samples were used for exploratory purposes to identify preliminary clues of a potential association between Mb and Hp across diverse human pathological states. Direct clinical validation in future studies should prioritize cohorts of patients with rhabdomyolysis (e.g., crush syndrome)."

References:

[1] Nielsen FE, Cordtz JJ, Rasmussen TB, Christiansen CF. The Association Between Rhabdomyolysis, Acute Kidney Injury, Renal Replacement Therapy, and Mortality. Clin Epidemiol. 2020 Sep 24;12:989-995. doi: 10.2147/CLEP.S254516.

[2]Ahmed K, Abdelrahman H, El-Menyar A, Saqr M, Silva AD, Alkahky SM, Al Qahtani J, Mekkodathil A, Al-Thani H, Peralta R. Clinical implications of serum myoglobin in trauma patients: A retrospective study from a level 1 trauma center. Int J

Crit Illn Inj Sci. 2020 Oct-Dec;10(4):170-176. doi: 10.4103/IJCIIS.IJCIIS_71_19.

2. If haptoglobin binds myoglobin, its concentration should be reduced, as is the case during intravascular haemolysis when it binds to haemoglobin. During sepsis, the systemic inflammatory response is such that haptoglobin may be induced.

Response: This is a critical observation that highlights the challenge of interpreting Hp level changes within a complex pathophysiological context. Our data precisely reflect these two competing processes: induction (increased synthesis as part of the acute-phase response) versus consumption (ligand clearance). You are absolutely correct that in the classic model of acute intravascular hemolysis, the acute-phase protein Hp binds hemoglobin (Hb) and is rapidly cleared via CD163-mediated phagocytosis, leading to a sharp decline in plasma Hp concentration, which is a physiological basis for its role as a diagnostic marker. However, in our RM-AKI mouse model, we observed a more complex dynamic process (**Fig. 1N–T**). In the early phase after injury (within 12 h), Hp levels in both renal tissue and serum were significantly elevated (**Fig. 1N–O, R, T**). This initial rise is most likely attributable to the acute-phase response, where the liver synthesizes and secretes large amounts of Hp in response to inflammatory signals. Subsequently, after 12 h, despite ongoing injury (as indicated by continually rising Mb levels, **Fig. 1N, P, R-S**), endogenous Hp levels began to decline, returning to baseline by 48 hours (**Fig. 1N–O, R, T**). This pattern of an "initial induction followed by subsequent decline" strongly suggests that after the acute-phase-induced peak, endogenous Hp is gradually consumed as excess Mb continues to bind.

Your point regarding the potential induction of Hp during sepsis is highly relevant. In Fig. 1L, we observed that serum Hp levels in a sepsis patient with concomitant RM were slightly elevated compared with those of healthy controls but remained within the normal range (0.3–2 g/L). Considering the extremely high circulating Mb levels in this patient (**Fig. 1K**), we speculate a balance may exist: systemic inflammation induces Hp synthesis, while concurrently released Mb consumes a portion of it. As a result, Hp may not be fully depleted, but its absolute amount is insufficient to neutralize all the

excess Mb. As we stated in the Discussion section: "this limited endogenous Hp elevation appears insufficient to counterbalance the pathological impacts of excessive Mb accumulation".

Crucially, regardless of its concentration changes, we provide direct molecular evidence via immunofluorescence co-localization (**Fig. 1U-V**) and immunoprecipitation (**Fig. 1W–Z**) that a physical complex forms between endogenous Hp and Mb in both the serum and kidneys of RM-AKI mice. This confirms the occurrence of the binding event, regardless of the trend in its concentration changes.

Therefore, in the revised manuscript, we will strengthen the description of the results in Fig. **1N–T** in the "Results" section ("Native haptoglobin binds excess myoglobin in RM-AKI mice") to explicitly state that the change in Hp levels likely reflects the superimposition of "acute-phase induction" and "post-ligand-binding consumption". Furthermore, we will cite this data in the "Discussion" section to support the argument that endogenous Hp is insufficient to cope with the massive Mb release during RM-AKI. The specific revisions in the manuscript are as follows:

In the Results section of "Native haptoglobin binds excess myoglobin in RM-AKI mice", we have added the following contents (shown with underlines):

"Next, Mb contents in the renal tissue of the modeling group gradually increased from 6 h **(Fig. 1N, P, R-S and Fig. EV1I)**. In comparison, the endogenous expression level of Hp peaked at 12 h and then gradually decreased **(Fig. 1N-O, R, T and Fig. EV1J)**. The dynamic change of Hp levels showing a 'rise first and then fall' may reflect the superimposition of two processes: 'acute phase induction' and 'consumption after ligand binding'."

We have added the following contents in the Discussion section (shown with underlines):

"Our results indicated that native Hp binds free Mb in serum and kidneys. The endogenous expression level of Hp peaked at 12 h and then gradually decreased in the RM-AKI mouse model **(Fig. 1)**. This 'rise first, then fall' dynamic strongly suggests that, after the acute-phase reaction-induced peak, endogenous Hp is gradually depleted due to continued binding with excess Mb. That is, endogenous Hp proteins

are depleted and insufficient to neutralize and remove all free Mb. Therefore, we must add additional Hp combined with free Mb to treat RM-AKI mice."

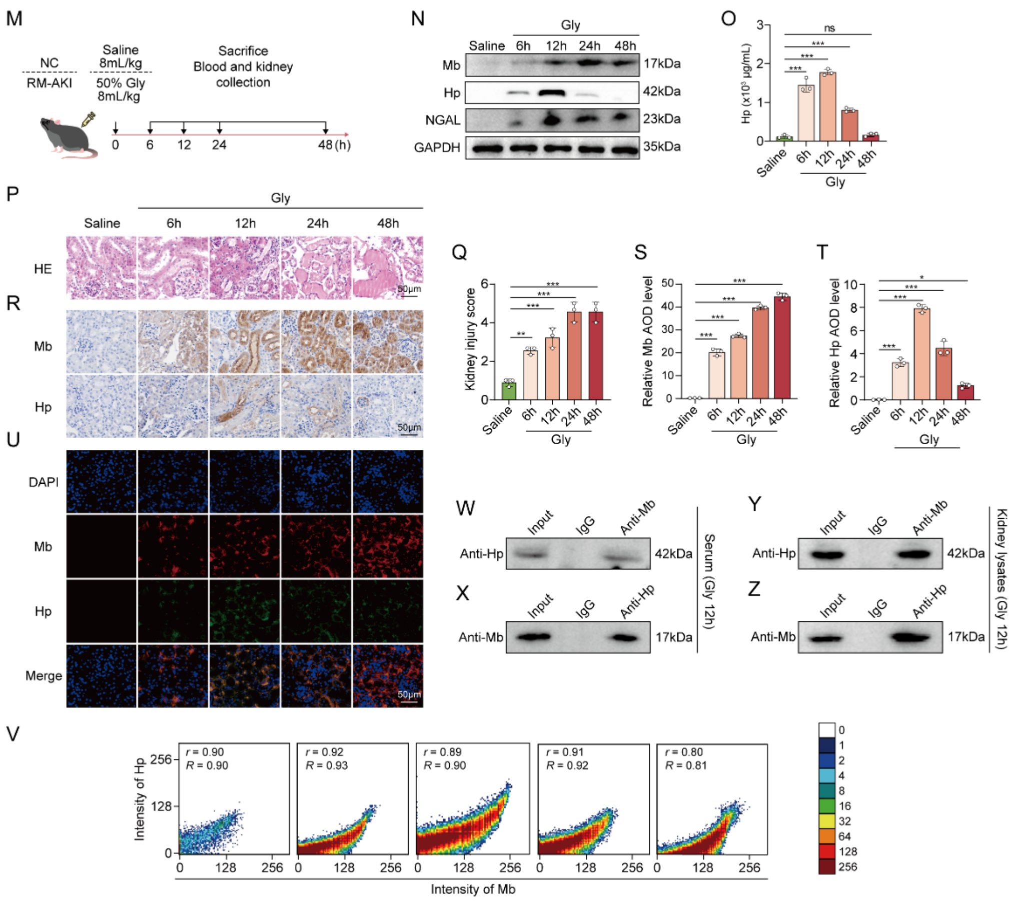

Fig. 1 Association between endogenous myoglobin and haptoglobin in human pathological conditions, and native haptoglobin binds to excess myoglobin in RM-AKI mice

(M) Schematic drawing of RM-AKI mice model preparation and sample collection.

(N) WB analysis of Mb, Hp, and NGAL expression in the mouse renal cortex in the Saline and Gly groups.

(O) ELISA analysis of the concentrations of Hp in the serum of the Saline and Gly groups (n = 3 per group for biological replicates).

(**P**) Representative HE staining in the kidney of the Saline and Gly group mice.

(**Q**) Kidney injury scores (0-5) were valued and quantified according to histopathological images of kidneys in Fig.1P (n = 3 per group for biological replicates).

(**R**) Representative immunohistochemical staining of Mb and Hp in the kidney of the Saline and Gly group mice.

(**S-T**) Quantification of Immunohistochemical data of Mb (**S**) and Hp (**T**) expression in Fig.1R (n = 3 per group for biological replicates).

(U) Representative confocal microscopy images of sections from kidneys harvested in Saline and Gly group mice stained for Mb (red), Hp (green), and DAPI (blue).

(V) The fluorescence co-localization analysis of Mb and Hp via the ImageJ plugin ScatterJ. The closer the R-value is to 1, the more likely it is that Mb and Hp will co-localize.
(W-X) Co-IP of Mb and Hp from the mouse serum of the Gly-12 h group. IP with IgG control antibody, anti-Mb antibody **(W)**, or anti-Hp antibody **(X)**.
(Y-Z) Co-IP of Mb and Hp from the renal cortex of RM-AKI mice (Gly-12 h group). IP with IgG control antibody, anti-Mb antibody **(Y)**, and anti-Hp antibody **(Z)**.
For statistical analysis, the unpaired two-tailed t-test (A-H) and One-Way ANOVA (O, Q, S-T) were used. Data are expressed as mean ± SD. **p* < 0.05, ** *p* < 0.01, *** *p* < 0.001, ns: not significant. Source data for this figure are available online.

Murine model

1. myoglobin appears to be in the tubular cells, but not in the lumens, which indicates reabsorption. Conventional histology and IHC staining for myoglobin should be performed to better assess the lesion profile and identify casts.

Response: We thank the reviewer for this insightful observation. We added the results of pathological staining (HE) for the normal control (Saline) group and the RM-AKI model group. At the same time, accumulation of Mb protein in the kidneys was detected by immunohistochemistry. We have added the corresponding description in the main text, as follows (shown with underlines):

"Meanwhile, in the HE staining assay, the renal cortex of RM-AKI mice exhibited pronounced pathological changes, including detachment of the tubular brush border, partial glomerular atrophy, and myoglobin casts (**Fig. 1P-Q**). In the modeling group, the accumulation of Mb, a critical pathogenic factor of RM-AKI, was increased in the kidneys, forming myoglobin casts **(Fig. 1N, P-S and Fig. EV1I)**."

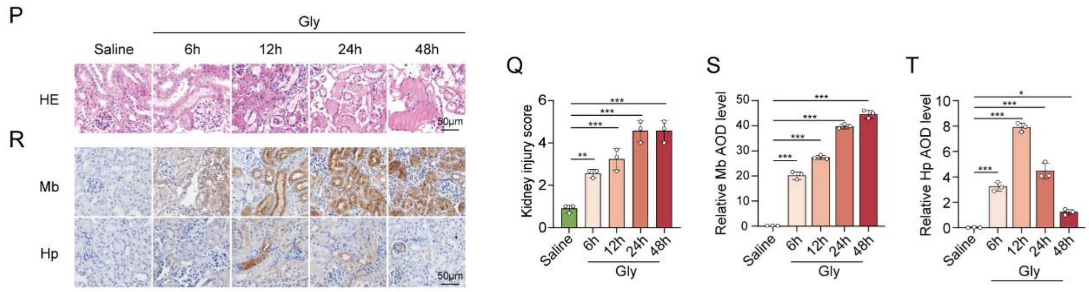

Fig. 1 Association between endogenous myoglobin and haptoglobin in human pathological conditions, and native haptoglobin binds to excess myoglobin in RM-AKI mice
(**P**) Representative HE staining in the kidney of the Saline and Gly group mice.

(**Q**) Kidney injury scores (0-5) were valued and quantified according to histopathological images of kidneys in Fig.1P (n = 3 per group for biological replicates).
(**R**) Representative immunohistochemical staining of Mb and Hp in the kidney of the Saline and Gly group mice.
(**S-T**) Quantification of Immunohistochemical data of Mb (**S**) and Hp (**T**) expression in Fig.1R (n = 3 per group for biological replicates).

2. It is surprising that the replicates in Fig. 1 O, Q and R show no variance.

Response: We appreciate the reviewer's attention to data presentation. To increase data reproducibility, we have repeated the experiments with three biological replicates. The new data is presented as follows:

Fig.1O

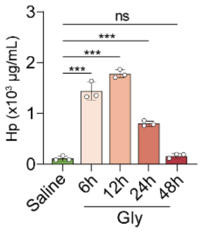

Fig. EV1

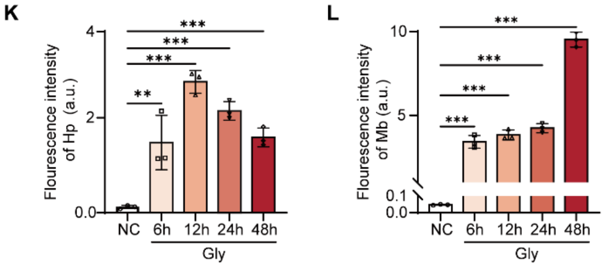

Fig. 1 Association between endogenous myoglobin and haptoglobin in human pathological conditions, and native haptoglobin binds to excess myoglobin in RM-AKI mice

(**O**) ELISA analysis of the concentrations of Hp in the serum of the Saline and Gly groups (n = 3 per group for biological replicates).

Figure EV1. Association between Mb and Hp in disease

(**K-L**) Quantification of Immunohistochemical data of Mb (**K**) and Hp (**L**) expression in **Fig.1U** (n = 3 per group for biological replicates).

Figure 3.

Treatment of mice with GST-Hpb: theoretically, the product should be injected at the time of diagnosis of rhabdomyolysis, as this is how the treatment will be prescribed in patients, i.e. when CPK levels rise above a critical threshold and before the onset of ARF.

Response: We thank the reviewer for raising this critical point regarding the timing of therapeutic intervention, which is paramount for clinical translation. We fully agree that the ideal therapeutic window is after the clinical diagnosis of rhabdomyolysis (RM) but before the onset of established acute kidney injury (AKI). Our experimental design was precisely intended to simulate this "early intervention" scenario and evaluate its efficacy.

In our primary therapeutic protocol (**Fig. 3A**), GST-Hpβ was administered intraperitoneally (i.p.) or intravenously (i.v.) immediately following the intramuscular injection of glycerol to induce RM-AKI. Previous studies have established that muscle damage commences within 1-3 hours after glycerol injection, accompanied by a sharp rise in creatine kinase (CK) and myoglobin (Mb) levels [1-4]. Therefore, administration "immediately after modeling" effectively simulates a therapeutic intervention at an early stage of RM-AKI development, prior to the manifestation of significant renal functional impairment. This aligns logically with the clinical scenario you described: intervention when CK levels exceed a critical threshold but before acute renal failure is established.

As demonstrated by our efficacy data, this early intervention significantly improved renal function at 24 hours (reduced SCr and BUN levels, ameliorated GFR) (**Fig. 3B-H**) and attenuated renal histopathological injury (**Fig. 3I-M**). This confirms the effectiveness of administering treatment promptly after the initiation of the pathological cascade.

Furthermore, this experimental paradigm of immediate post-glycerol intervention (within 0-2 hours) is well-established and widely adopted in the field for evaluating the efficacy of various therapeutic agents in RM-AKI mouse and rat models [2-8]. Our design is consistent with this standard methodological approach.

Accordingly, in the revised manuscript, we have clarified in the "Results" section ("Early recombinant Hpβ fusion protein injection alleviates RM-AKI in mice") and in

the legend of Figure 3A that the "immediately after modeling" design was implemented to simulate early clinical intervention. The details are as follows (underline highlight):

In the Results section of "Early recombinant Hpβ fusion protein injection alleviates RM-AKI in mice", we emphasize the early stage as follows:

"To verify the early-stage therapeutic effect of the GST-Hpβ fusion protein in RM-AKI mice (**Fig. 3A**), we first need to explore the optimal administration mode and dosage after modeling."

In the caption of Fig. 3, Recombinant Hpβ fusion protein injection alleviates RM-AKI in vivo,

"**(A)** Experimental design of GST-Hpβ fusion protein treatment for RM-AKI mice. Intramuscular glycerol injection (Gly i.m.) to prepare RM-AKI model mice. Meanwhile, GST-Hpβ or GST is injected intravenously (i.v.) or intraperitoneally (i.p.), respectively, to simulate early-stage clinical intervention. After 24 hours, renal function is measured by GFR. Blood and tissue samples are collected for biochemical testing and pathological analysis."

References:

[1] Nara A, Yajima D, Nagasawa S, Abe H, Hoshioka Y, Iwase H. Evaluations of lipid peroxidation and inflammation in short-term glycerol-induced acute kidney injury in rats. *Clin Exp Pharmacol Physiol* 2016, 43(11)**:** 1080-1086.

[2] Yang FL, Subeq YM, Chiu YH, Lee RP, Lee CJ, Hsu BG. Recombinant human erythropoietin reduces rhabdomyolysis-induced acute renal failure in rats. *Injury* 2012, 43(3)**:** 367-373.

[3] Subeq YM, Wu WT, Lee CJ, Lee RP, Yang FL, Hsu BG. Pentobarbital reduces rhabdomyolysis-induced acute renal failure in conscious rats. *J Trauma* 2009, 67(1)**:** 132-138.

[4] Wang Z, Shah SV, Liu H, Baliga R. Inhibition of cytochrome P450 2E1 and activation of transcription factor Nrf2 are renoprotective in myoglobinuric acute kidney injury. *Kidney Int* 2014, 86(2)**:** 338-349.

[5] Shi Y, Xu L, Tang J, Fang L, Ma S, Ma X, Nie J, Pi X, Qiu A, Zhuang S, Liu N. Inhibition of HDAC6 protects against rhabdomyolysis-induced acute kidney injury. *Am J Physiol Renal Physiol* 2017, 312(3)**:** F502-f515.
[6] Wang S, Zhang C, Li J, Niyazi S, Zheng L, Xu M, Rong R, Yang C, Zhu T. Erythropoietin protects against rhabdomyolysis-induced acute kidney injury by modulating macrophage polarization. *Cell Death Dis* 2017, 8(4)**:** e2725.
[7] Wang X, Li N, Han L, Qiao O, Chen X, Wang P, Zhang L, Hou Y, Bao F, Hao H, Saeed S, Zhang L, Li Z, Duan X, Rao S, Liu Z, Gong Y. Rescue RM/CS-AKI by blocking strategy with one-dose anti-myoglobin RabMAb. *Nat Commun* 2025, 16(1)**:** 1044.
[8] Zhao X, Sun J, Dong J, Guo C, Cai W, Han J, Shen H, Lv S, Zhang R. An auto-photoacoustic melanin-based drug delivery nano-platform for self-monitoring of acute kidney injury therapy via a triple-collaborative strategy. *Acta Biomater* 2022, 147**:** 327-341.

The saline and treatment groups are not comparable, as the saline group receives lower volumes than the treatment group.

Response: Thank you for highlighting this important detail regarding experimental design. In the animal experiments, the injection volume was carefully standardized. Mice weighing 20–25 g in the model group (Gly group) received an intramuscular injection of 160–200 μL of 50% glycerol (diluted in saline), corresponding to a final dose of 8.0 mL/kg. Mice in the normal control group (Saline group) received an equal volume intramuscular injection of 160–200 μL of saline. This protocol ensured volumetric consistency between the saline control and the glycerol model solution. The comparison between these two groups was primarily intended to validate the successful induction of the RM-AKI model.

Regarding the treatment groups, if the difference in administered fluid volume alone were responsible for improved renal function, the group receiving an equal volume (200 μL) of the control GST protein ("Gly+GST") should have shown benefits similar to those of the "Gly+GST-Hpβ" treatment group. However, the data in Fig. 3B–

S clearly show that only the "Gly+GST-Hpβ" treatment group produced a significant renoprotective effect. In contrast, the "Gly+GST" control group showed no statistically significant difference from the Gly model group. This provides compelling evidence that the therapeutic efficacy originates from the specific function of the GST-Hpβ fusion protein, rather than from the injection volume or the GST tag itself.

Furthermore, during the experimental process, we initially included a "Gly+PBS" group, in which glycerol-modeled mice received an equal volume injection (200 μL) of PBS — the vehicle used to dissolve both GST and GST-Hpβ. The results from this group were similar to those of the "Gly+GST" group (**Fig. a-c**). To enhance the clarity and focus of the manuscript for readers, we presented the "Gly+GST" group as the primary vehicle control for the therapeutic experiments, as it most directly addresses potential confounding effects from the protein components of the solution.

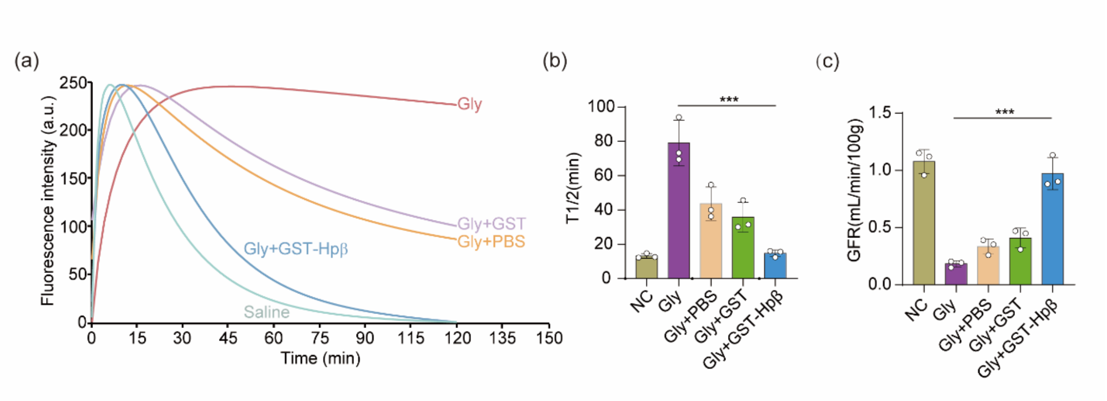

Fig. GST-Hpβ treatment improves kidney function in RM-AKI mice

(a) Representative image of transcutaneous disappearance curves of FITC-sinistrin excretion after 24 h in i.p. injection with 10 mg/kg GST-Hpβ group.

(b) The excretion half-life ($T_{1/2}$) of FITC-sinistrin in the selected groups of mice (n = 3 per group for biological replicates).

(c) GFR of the selected groups of mice was measured at 24h (n = 3 per group for biological replicates).

Since hydration and alkalisation are the standard treatments, a hyperhydration/alkalisation arm is needed.

Response: We thank the reviewer for this suggestion. Firstly, it should be noted that fluid resuscitation and urinary alkalinization are not considered the undisputed gold standard for the treatment of rhabdomyolysis-associated acute kidney injury (RM-AKI)

[1,2]. In emergency medical settings, fluid resuscitation serves as a non-specific, foundational supportive measure with limited and variable efficacy—a primary reason for pursuing novel therapeutic strategies [3,4]. While early fluid resuscitation in rhabdomyolysis is aimed at maintaining renal tubular blood flow, flushing casts, reducing intratubular Mb accumulation and toxicity, and ensuring adequate renal perfusion to prevent AKI, there remains significant controversy over the optimal crystalloid solution [5]. Furthermore, the fluid requirement and infusion rate are patient-specific; patients with persistent oliguria or anuria may ultimately require continuous renal replacement therapy (CRRT). The utility of urine alkalinization is also debated, with no robust clinical evidence strongly supporting the use of sodium bicarbonate and/or mannitol to prevent AKI in rhabdomyolysis [6].

Our study is fundamentally a mechanistic exploration and proof-of-concept preclinical investigation. Its primary goal was to validate the feasibility and efficacy of a novel biotherapeutic strategy—the engineered GST-Hpβ fusion protein—which aims to prevent and treat RM-AKI by binding circulating Mb and directing its clearance via macrophages. Consequently, the experimental design focused on demonstrating that this strategy is superior to no targeted intervention (the Gly model group) and elucidating its mechanism of action. This approach, focusing on establishing efficacy against the disease model, is standard in foundational therapeutic development for RM-AKI [7-10].

Given the practical challenges of implementing a continuous, titrated fluid resuscitation protocol in mice and the core objective of evaluating the novel biotherapeutic (recombinant GST-Hpβ) itself, our study used appropriate vehicle controls. We tested an equal volume PBS injection group (Gly+PBS), but its results were comparable to those of the Gly+GST control group (**Fig.a-c**). To maintain clarity and focus in presenting the central therapeutic concept, we presented the Gly+GST group as the primary control for the rescue experiments.

We fully agree that, in the next step toward clinical translation, including a control group receiving hyperhydration/alkalinization (mimicking foundational supportive care) will be essential. Such a study design will be crucial to determine whether the GST-Hpβ fusion protein strategy could serve as a replacement therapy or, more likely,

provide additional renal protection on top of foundational fluid resuscitation/alkalinization, which are critical for defining its clinical positioning

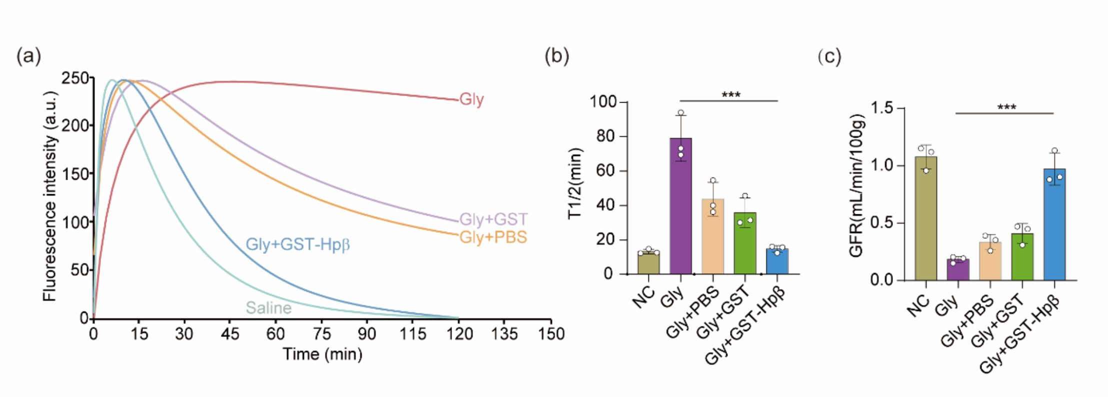

Fig. GST-Hpβ treatment improves kidney function in RM-AKI mice

(a) Representative image of transcutaneous disappearance curves of FITC-sinistrin excretion after 24 h in i.p. injection with 10 mg/kg GST-Hpβ group.

(b) The excretion half-life ($T_{1/2}$) of FITC-sinistrin in the selected groups of mice (n = 3 per group for biological replicates).

(c) GFR of the selected groups of mice was measured at 24h (n = 3 per group for biological replicates).

References:

[1] Honore PM, De Bels D, Spapen HD. Beneficial effects of antioxidant therapy in crush syndrome in a rodent model: enough evidences to be used in humans? *Ann Intensive Care* 2018, 8(1)**:** 96.

[2] Khan Z, Ibekwe M, Abumedian M, Yousif Y, Mlawa G. A Rare Presentation of Acute Renal Failure Secondary to Rhabdomyolysis in a Patient Due to Atorvastatin Requiring Short-Term Renal Replacement Therapy. *Cureus* 2022, 14(3)**:** e23511.

[3] Chavez LO, Leon M, Einav S, Varon J. Beyond muscle destruction: a systematic review of rhabdomyolysis for clinical practice. *Crit Care* 2016, 20(1)**:** 135.

[4] Xiao L, Ran X, Zhong Y, Le Y, Li S. Serum creatine kinase levels are not associated with an increased need for continuous renal replacement therapy in patients with acute kidney injury following rhabdomyolysis. *Ren Fail* 2022, 44(1)**:** 893-901.

[5] Scharman EJ, Troutman WG. Prevention of kidney injury following rhabdomyolysis: a systematic review. *Ann Pharmacother* 2013, 47(1)**:** 90-105.

[6] Homsi E, Barreiro MF, Orlando JM, Higa EM. Prophylaxis of acute renal failure in patients with rhabdomyolysis. *Ren Fail* 1997, 19(2)**:** 283-288.

[7] Wang S, Zhang C, Li J, Niyazi S, Zheng L, Xu M, Rong R, Yang C, Zhu T. Erythropoietin protects against rhabdomyolysis-induced acute kidney injury by modulating macrophage polarization. *Cell Death Dis* 2017, 8(4)**:** e2725.

[8] Wang X, Li N, Han L, Qiao O, Chen X, Wang P, Zhang L, Hou Y, Bao F, Hao H, Saeed S, Zhang L, Li Z, Duan X, Rao S, Liu Z, Gong Y. Rescue RM/CS-AKI by blocking strategy with one-dose anti-myoglobin RabMAb. *Nat Commun* 2025, 16(1)**:** 1044.

[9] Yu JG, Fan BS, Guo JM, Shen YJ, Hu YY, Liu X. Anisodamine Ameliorates Hyperkalemia during Crush Syndrome through Estradiol-Induced Enhancement of Insulin Sensitivity. *Front Pharmacol* 2019, 10**:** 1444.

[10] Zhang BF, Wang PF, Cong YX, Lei JL, Wang H, Huang H, Han S, Zhuang Y. Anti-high mobility group box-1 (HMGB1) antibody attenuates kidney damage following experimental crush injury and the possible role of the tumor necrosis factor-α and c-Jun N-terminal kinase pathway. *J Orthop Surg Res* 2017, 12(1)**:** 110.

A histological study with IHC staining for myoglobin would be required.

Response: Thank you for your construction suggestion. We have added the immunohistochemical (IHC) staining results for myoglobin (Fig. 3K, L). The specific results are as follows:

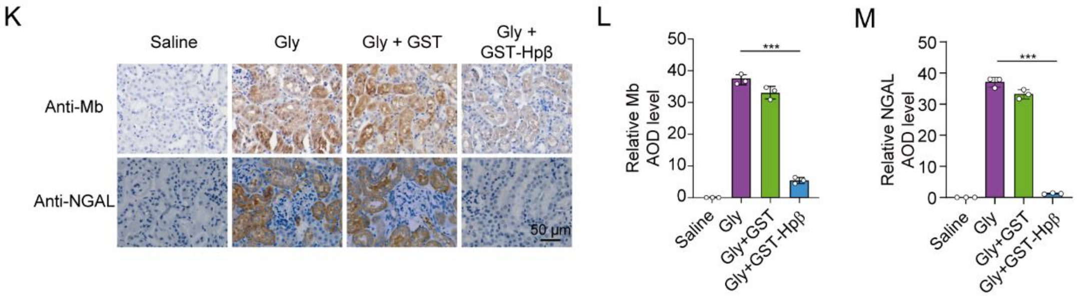

Fig. 3 Recombinant Hpβ fusion protein injection alleviates RM-AKI *in vivo*
(K) Representative IHC staining images of Mb and NGAL expression in kidney tissues. **(L-M)** Quantification of the integrated optical density (IOD) of Mb (**L**) and NGAL (**M**) in the image of (K) (n = 3 per group for biological replicates).

Here again, there is virtually no variability between the six replicates for the study of renal function.

Response: We appreciate the reviewer's attention to data presentation. To increase data reproducibility, we have repeated the experiments with three biological replicates. The new data is presented as follows:

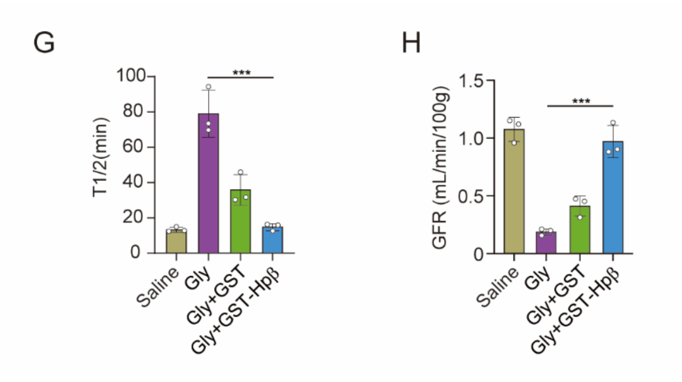

Fig. 3 Recombinant Hpβ fusion protein injection alleviates RM-AKI *in vivo*
(G) The excretion half-life ($T_{1/2}$) of FITC-sinistrin in the selected groups of mice (n = 3 per group for biological replicates).
(H) GFR of the selected groups of mice was measured at 24h (n = 3 per group for biological replicates).

Figure 6.

Finally, GST-Hpb is eliminated via the urinary tract (fluorescence is found in the bladder). There is no fixation in the spleen, as might be expected?

Response: Thank you for your question. Previously, in order to obtain fluorescence images with low background, we set the fluorescence threshold too high, which caused us to overlook some valuable information and led to your concerns. We sincerely apologize for this. In the current revision, we strictly followed the instrument operation manual and relevant experimental protocols, and reanalyzed the *in vivo* fluorescence imaging data of the mice, selecting the instrument's default optimal fluorescence range. The results showed fluorescence signals near the mouse spleen in the abdominal cavity, which is largely consistent with our preliminary expectations for the *in vivo* distribution of GST-Hpβ. In addition, the fluorescently labeled protein

may be metabolized in the body, with some eliminated through the bladder and urine, resulting in obvious fluorescence signals in the bladder. We are now providing the images again as follows:

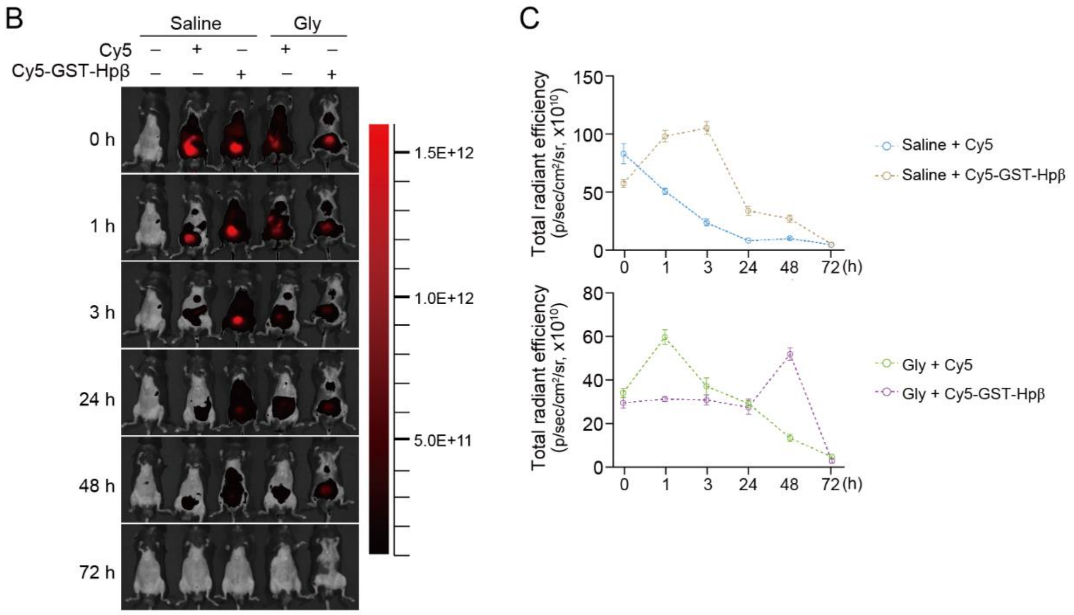

Fig. 6 Metabolic imaging of Hpβ fusion protein in RM-AKI mice
(B) *In vivo* imaging of each group of mice within 72 hours.
(C) The fluorescence intensity of each group of mice at different time points.

2nd Mar 2026

Dear Prof. Gong,

Thank you for the submission of your revised manuscript to EMBO Molecular Medicine. We have heard back from the three referees who agreed to re-evaluate your manuscript. As you will see from the reports below, referees #1 and #2 are supporting publication. However, as previously communicated, #3 raises concerns about the number of replicates. We also thank you for providing a response to these points.

After consultation with my colleagues here, we concluded that raised concerns are justified. Therefore, in an additional and final round of major revision, the results from all biological replicates (from both initial submission and revised version) should be presented. Statistical analyses including all biological replicates must be performed for all experiments in which replicates were excluded during first revision. Please also upload source data for all biological replicates with your revised manuscript. In addition, referee #3 points regarding the experimental model should be discussed in greater detail and limitations of the study in this respect should be clearly stated in the manuscript.

Further consideration of a revision that addresses reviewer's concerns in full will entail an additional round of review. Acceptance or rejection of the manuscript will depend on the completeness of your responses included in the next, final version of the manuscript. For this reason, and to save you from any frustrations in the end, I would strongly advise against returning an incomplete revision.

Please also amend following points:
1) Author checklist: Please complete the form by selecting appropriate responses in all column D fields.
2) Source data: Please upload source data files as one folder per figure either to BioStudies (https://www.ebi.ac.uk/biostudies/) or our submission system.
3) Supplementary information: The methods from the supplementary file should be moved to the manuscript text. The EV figures should be uploaded as separate, high resolution figure files and their legends should be added to the manuscript text, after the main figure legends. Table EV1 should be uploaded as a separate file. The western blot source data should be uploaded as source data files and zipped with the numerical and image data, as one zip file per figure.
4) In the main manuscript file, please do the following:
- Please address all comments suggested by our data editors listed below:
o Figure legends:
1. Please note that the exact p values are not provided in the legends of figures 1A-L, O, Q, S, T; 3B, C, D, E, G, H, J, L, M, N, P, Q, R, S; 5C, G, H, I, J, L, N; EV1 B, C, D, F, G, H-L.
2. Please indicate the statistical test used for data analysis in the legend of figure EV1 A.
3. Please note that information related to n is missing in the legends of figures 1C-L; 3N-S; 4A, B; 6C, 1B-E, K, L; EV2 B.
4. Please note that the error bars are not defined in the legends of figures 4A, B; 6C, EV2 B.
- Add callouts for Fig 6C and Table EV1.
- Author contributions: Please remove it from the manuscript. CRediT has replaced the traditional author contributions section because it offers a systematic machine-readable author contributions format that allows for more effective research assessment. You are encouraged to use the free text boxes beneath each contributing author's name to add specific details on the author's contribution. More information is available in our guide to authors:
https://www.embopress.org/page/journal/17574684/authorguide#authorshipguidelines
- In data availability statement please remove "All source data for the experiments are available with this manuscript. Source data are included with this paper." If you decide to upload source data files to BioStudies please provide the access link using the following format:
The source data are available in the following database:
[full name of the resource] [accession number/identifier] ([doi or URL or identifiers.org/DATABASE:ACCESSION])
5) The Paper Explained: Please add it to the main manuscript text.
6) Synopsis:
- Synopsis image: Please upload the image as a separate, high-resolution jpeg file 550 px-wide x 300-600 pixels high to illustrate your article.
- Please check your synopsis text and image before submission with your revised manuscript.

We would welcome the submission of a revised version within three months for further consideration. Please let us know if you require longer to complete the revision.

I look forward to seeing a revised form of your manuscript as soon as possible.

I look forward to receiving your revised manuscript.

Yours sincerely,

Zeljko Durdevic

Zeljko Durdevic
Senior Editor
EMBO Molecular Medicine

*****
When preparing your revised manuscript, please refer to our guidelines: https://link.springer.com/journal/44321/submission-guidelines#cms-Revised-submissions. We perform an initial quality control of all revised manuscripts before re-review; failure to include requested items will delay the evaluation of your revision.

We require:

1) A .docx formatted version of the manuscript text (including legends for main figures, EV figures and tables). Please make sure that the changes are highlighted to be clearly visible.

2) Individual production quality figure files as .eps, .tif, .jpg (one file per figure). For guidance, download the 'Figure Guide PDF': https://media.springernature.com/original/springer-cms/rest/v1/content/27825798/data/v1.

3) A .docx formatted letter INCLUDING the reviewers' reports and your detailed point-by-point responses to their comments. As part of the EMBO Press transparent editorial process, the point-by-point response is part of the Review Process File (RPF), which will be published alongside your paper.

4) A complete author checklist, which you can download from our author guidelines. Please insert information in the checklist that is also reflected in the manuscript. The completed author checklist will also be part of the RPF.

5) Please note that all corresponding authors are required to supply an ORCID ID for their name upon submission of a revised manuscript.

6) It is mandatory to include a 'Data Availability' section after the Materials and Methods. Before submitting your revision, primary datasets produced in this study need to be deposited in an appropriate public database, and the accession numbers and database listed under 'Data Availability'. Please remember to provide a reviewer password if the datasets are not yet public.

In case you have no data that requires deposition in a public database, please state so in this section. Note that the Data Availability Section is restricted to new primary data that are part of this study.

7) For data quantification: please specify the name of the statistical test used to generate error bars and P values, the number (n) of independent experiments (specify technical or biological replicates) underlying each data point and the test used to calculate p-values in each figure legend. The figure legends should contain a basic description of n, P and the test applied. Graphs must include a description of the bars and the error bars (s.d., s.e.m.).

8) At EMBO Press we ask authors to provide source data for the main manuscript figures. You will receive a separate email with instructions for providing source data with your revised manuscript, including how to upload and organize the files.

9) Our journal encourages inclusion of *data citations in the reference list* to directly cite datasets that were re-used and obtained from public databases. Data citations in the article text are distinct from normal bibliographical citations and should directly link to the database records from which the data can be accessed. In the main text, data citations are formatted as follows: "Data ref: Smith et al, 2001" or "Data ref: NCBI Sequence Read Archive PRJNA342805, 2017". In the Reference list, data citations must be labeled with "[DATASET]". A data reference must provide the database name, accession number/identifiers and a resolvable link to the landing page from which the data can be accessed at the end of the reference.

10) We replaced Supplementary Information with Expanded View (EV) Figures and Tables that are collapsible/expandable online. A maximum of 5 EV Figures can be typeset. EV Figures should be cited as 'Figure EV1, Figure EV2" etc... in the text and their respective legends should be included in the main text after the legends of regular figures.

- For the figures that you do NOT wish to display as Expanded View figures, they should be bundled together with their legends

in a single PDF file called *Appendix*, which should start with a short Table of Content. Appendix figures should be referred to in the main text as: "Appendix Figure S1, Appendix Figure S2" etc.

- Additional Tables/Datasets should be labeled and referred to as Table EV1, Dataset EV1, etc. Legends have to be provided in a separate tab in case of .xls files. Alternatively, the legend can be supplied as a separate text file (README) and zipped together with the Table/Dataset file.

11) The paper explained: EMBO Molecular Medicine articles are accompanied by a summary of the articles to emphasize the major findings in the paper and their medical implications for the non-specialist reader. Please provide a draft summary of your article highlighting

- the medical issue you are addressing,

- the results obtained and

- their clinical impact.

This may be edited to ensure that readers understand the significance and context of the research. Please refer to any of our published articles for an example.

12) Author contributions: You will be asked to provide CRediT (Contributor Role Taxonomy) terms in the submission system. These replace a narrative author contribution section in the manuscript.

13) A Conflict of Interest statement should be provided in the main text.

14) Every published paper includes a 'Synopsis' to further enhance discoverability. Synopses are displayed on the journal webpage and are freely accessible to all readers. They include a short stand first (maximum of 300 characters, including space) as well as 2-5 one-sentences bullet points that summarizes the paper. Please write the bullet points to summarize the key NEW findings. They should be designed to be complementary to the abstract - i.e. not repeat the same text. We encourage inclusion of key acronyms and quantitative information (maximum of 30 words / bullet point). Please use the passive voice. Please attach these in a separate file or send them by email, we will incorporate them accordingly.

Please also suggest a striking image or visual abstract to illustrate your article as a PNG file 550 px wide x 300-600 px high.

15) Include a Reagents and Tools Table as part of the Methods section, which can be downloaded from our author guidelines.

EMBO Molecular Medicine has a "scooping protection" policy, whereby similar findings that are published by others during review or revision are not a criterion for rejection. Should you decide to submit a revised version, I do ask that you get in touch after three months if you have not completed it, to update us on the status.

***** Reviewer's comments *****

Referee #1 (Comments on Novelty/Model System for Author):

The authors have well addressed all my concerns.

Referee #1 (Remarks for Author):

I am satisfied with the revisions and believe the manuscript is now suitable for publication.

Referee #2 (Remarks for Author):

The authors have addressed all my concerns.

Referee #3 (Remarks for Author):

Sepsis is not a suitable clinical model for studying the links between rhabdomyolysis and its effects on haptoglobin and AKI, given the number of confounding factors (inflammation, low flow, infections, antibiotics, etc.). The same applies to contrast-induced AKI. We need polytrauma or crush syndrome patients, which are more pure, with clearly elevated CPK/myoglobin values.

I don't understand how many mice were used in Figure 1 (RM-AKI mouse model: in the first version, there is mention of 6 per group, so 6 biological replicates a priori; in the revised version, there are only 3 biological replicates per group. But how can we move in V2 to a large dispersion of the 3 values when in V1 there is no dispersion for 6 replicates?

Yes, volume expansion is the standard treatment for preserving renal function during rhabdomyolysis. Alkalization is indeed more controversial.

In Figure 3, therapeutic intervention allows for total clearance of myoglobin in the tubules and total preservation of renal function within 24 hours, which is absolutely exceptional for a preclinical therapeutic intervention. This efficacy should be confirmed in another experimental model where rhabdomyolysis is involved in ARF: if the authors are convinced of the clinical relevance of sepsis in myoglobin-induced AKI, there are murine models of sepsis/AKI where this treatment can be tested, which will definitively validate this treatment.

Figure 3 G H. Again, I do not understand how we go from 6 to 3 replicates: how many mice were ultimately analyzed in each group? Regarding the absence of variance, I was referring to panels 3 B, C, D, E, where under certain conditions, the distribution of values is zero.

In Figure 6B, the in vivo imaging analyses do not show the Gly+(Cy5+GST+Hpbeta) condition: why?

Point-by-point responses

***** Reviewer's comments *****

Referee #1 (Comments on Novelty/Model System for Author):

The authors have well addressed all my concerns.

Referee #1 (Remarks for Author):

I am satisfied with the revisions and believe the manuscript is now suitable for publication.

Referee #2 (Remarks for Author):

The authors have addressed all my concerns.

Response: We sincerely thank Referees #1 and #2 for their time and for their positive assessment of our revisions. We also greatly appreciate Referee #3 for the continued critical evaluation, which has further strengthened our manuscript. Below, we provide point-by-point responses to the remaining concerns.

Referee #3 (Remarks for Author):

Sepsis is not a suitable clinical model for studying the links between rhabdomyolysis and its effects on haptoglobin and AKI, given the number of confounding factors (inflammation, low flow, infections, antibiotics, etc.). The same applies to contrast-induced AKI. We need polytrauma or crush syndrome patients, which are more pure, with clearly elevated CPK/myoglobin values.

Response: Thank you for your constructive suggestion. We fully agree with the reviewer that sepsis and contrast-induced AKI represent complex clinical conditions with multiple confounding factors, and that the ideal clinical validation for our findings would involve cohorts of patients with direct muscle injury, such as polytrauma or crush syndrome. We attempted to collaborate with hospitals to obtain blood samples from patients with polytrauma or crush syndrome, but unfortunately, we were unable to do so. Our original use of public human datasets and a single septic patient sample was intended to provide preliminary clinical clues regarding a potential association between

Mb and Hp across different pathological conditions. If this dataset raises any concerns, we are willing to remove the clinical data on septic patients and contrast-induced AKI, as the relationship between haptoglobin and Mb has been sufficiently demonstrated in our well-established glycerol-induced rhabdomyolysis-AKI mouse model. Furthermore, we will emphasize in the Discussion (Limitations section) that direct clinical validation in future studies should be conducted using cohorts of patients with rhabdomyolysis, such as those with crush syndrome.

I don't understand how many mice were used in Figure 1 (RM-AKI mouse model: in the first version, there is mention of 6 per group, so 6 biological replicates a priori; in the revised version, there are only 3 biological replicates per group. But how can we move in V2 to a large dispersion of the 3 values when in V1 there is no dispersion for 6 replicates?

Response: We thank the reviewer for the careful attention to the experimental details and data presentation in our revised manuscript. We apologize for the confusion caused by our representation. In Fig.1, there were 6 mice in each of the NC group and the RM-AKI group. When the biochemical indicators SCr and BUN concentrations were detected, we tested the 6 mice in each group separately, so n = 6 biological replicates (Fig EV. 1A-B）. However, for tissue Western blot, HE staining, immunohistochemistry, immunofluorescence, and blood Hp concentration testing, we randomly selected 3 samples from 6 mice per group, yielding n = 3 biological replicates.

In the V1 version, the 6 data points in Fig. 1O comprised 3 biological replicates and 2 technical replicates. Following your advice, we have repeated the experiments with three biological replicates to improve data reproducibility. Therefore, in the V2 version, we present the new data after 3 biological replicates. According to the editor's suggestion, the results from all biological replicates (from both the initial submission and the revised version) should be presented. Therefore, in this round of revisions, Fig. 1C (original Fig. 1O) shows data from six biological replicates: three from the initial submission and the other three from the V2 revision.

For immunofluorescence quantification of Mb and Hp proteins in Fig. 1I (original

Fig. 1Q-R in V1 version, revised Fig. EV1K-L in V2 version, revised Fig. EV1F-G in this version), the raw 6 data were statistically analyzed by 1 individual (including 3 biological replicates and 2 technical replicates). Thank you for your kind reminder. To increase the objectivity of the data statistics, 3 experienced senior students counted the 3 biological replicates for each group in the V2 version, and the average value of each group is taken as the final data point. We want to express our deep apologies again for the trouble caused by the lack of clarity.

Yes, volume expansion is the standard treatment for preserving renal function during rhabdomyolysis. Alkalization is indeed more controversial.

Response: We thank the reviewer for this confirmation. We fully acknowledge that volume expansion is the standard treatment for preserving renal function during rhabdomyolysis. Therefore, in the Discussion section, we added the sentence as follows (underline highlight):

"Moreover, for newly developed recombinant tandem multi-beta-subunit of Hp proteins in drug development, it is necessary to evaluate the circulating half-life and tissue distribution. In addition, given that fluid resuscitation is the standard clinical treatment for preserving renal function during rhabdomyolysis, subsequent evaluation of recombinant tandem multi-beta-subunit of Hp proteins, in combination with or compared to standard fluid resuscitation, will be critical for defining their clinical positioning."

In Figure 3, therapeutic intervention allows for total clearance of myoglobin in the tubules and total preservation of renal function within 24 hours, which is absolutely exceptional for a preclinical therapeutic intervention. This efficacy should be confirmed in another experimental model where rhabdomyolysis is involved in ARF: if the authors are convinced of the clinical relevance of sepsis in myoglobin-induced AKI, there are murine models of sepsis/AKI where this treatment can be tested, which will definitively validate this treatment.

Response: We appreciate the reviewer's recognition of the robust therapeutic effect observed in our glycerol-induced RM-AKI model. The glycerol-induced RM-AKI model has a relatively simple etiology, and our therapeutic approach neutralizes the key pathogenic factor (free myoglobin, Mb) directly at its source before it reaches the kidneys. Although GST-Hpβ treatment significantly improved renal function and reduced kidney injury in glycerol-induced RM-AKI mice, it did not achieve complete clearance of Mb from the renal tubules or fully restore renal function. As shown in Fig. 3J, the kidney injury score in the Gly+GST-Hpβ group (1.89 ± 0.19) remained higher than that in the saline control group (0.67 ± 0.33). Moreover, residual Mb accumulation was still detectable in the renal tubules of the Gly+GST-Hpβ group (Fig. 3K-L; the three replicate experiments and an enlarged image are shown below). Moreover, compared with the Saline group, the kidney tubular injury marker NGAL level is higher in the Gly+GST-Hpβ group (Fig. 3K, M; the three replicate experiments and enlarged image are shown below). Thus, the treatment did not completely eliminate myoglobin from the tubules or fully preserve renal function.

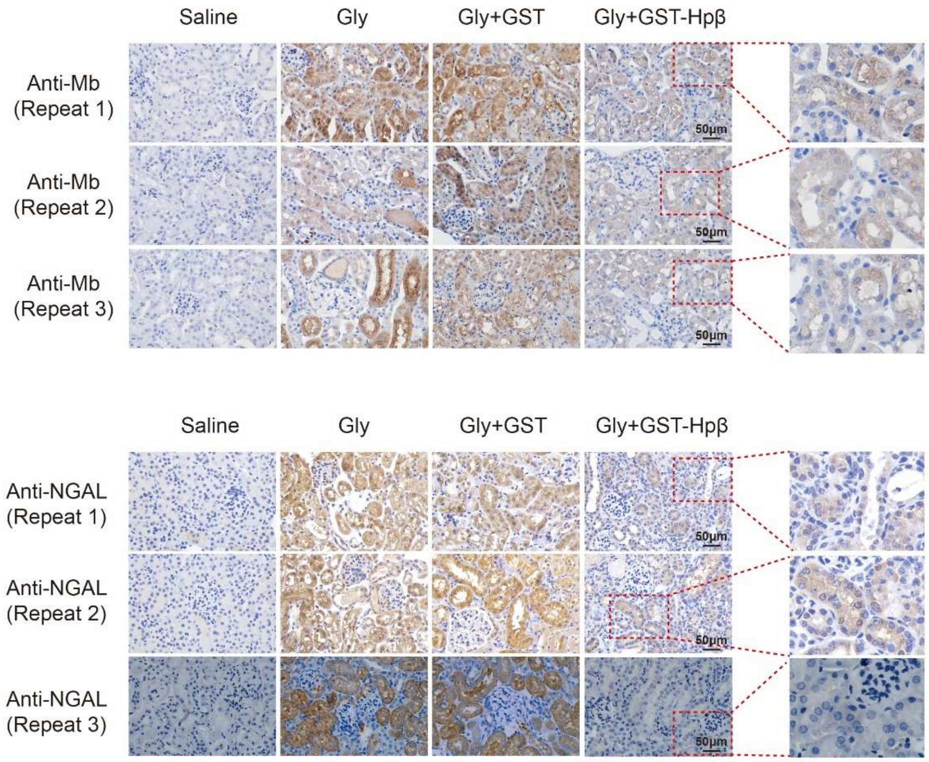

Fig. Figure 3K in the maintext, three repetitions of immunohistochemical staining results of Mb and NGAL in the kidney tissues of glycerol-induced RM-AKI mice, along

with enlarged images.

We are grateful to the reviewer for the constructive suggestion to validate our findings in an additional model. Rhabdomyolysis can be classified into non-traumatic rhabdomyolysis and traumatic rhabdomyolysis (also known as crush syndrome associated AKI, CS-AKI). The glycerol-induced model is the classic approach for non-traumatic RM, which is associated with causes such as excessive exercise, drug toxicity, high temperature, fever, and improper diet. Traumatic rhabdomyolysis, on the other hand, results from prolonged compression of muscle-rich limbs by heavy objects; upon decompression, muscle cells rupture, releasing intracellular contents such as myoglobin, K^+, and nucleic acids into the circulation, with AKI being one of its most serious complications. Traumatic rhabdomyolysis occurs frequently in disaster settings such as earthquakes, wars, and explosions.

Accordingly, we successfully established a mouse model of traumatic rhabdomyolysis-associated AKI (CS-AKI). Treatment with GST-Hpβ significantly improved renal function and reduced kidney injury in CS-AKI mice (**Fig. EV3A-K**). However, the pathogenic mechanisms underlying the CS model are more complex, involving not only myoglobin accumulation but also ischemia-reperfusion injury, oxidative stress, and so on. These factors likely explain why the variability among the six biological replicates was greater in the CS-AKI model than in the glycerol-induced RM-AKI model. Meanwhile, this also suggests that the therapeutic efficacy of GST-Hpβ in the more complex CS-AKI model (a traumatic RM model) is indeed less pronounced than in the simpler glycerol-induced RM-AKI model. This observation may, to some extent, explain why effective therapeutic outcomes observed at the laboratory level are often difficult to achieve in clinical AKI patients. Nevertheless, the protective efficacy of GST-Hpβ in RM-AKI mouse models remains affirmed.

We have now added a description of the CS-AKI rescue experiments and the corresponding data to the Results section of the revised manuscript, as follows:

"Furthermore, we validated the therapeutic efficacy of GST-Hpβ in a mouse model of traumatic rhabdomyolysis-associated acute kidney injury (crush syndrome associated AKI, CS-AKI) (**Fig. EV3A**). Compared with the control group (NC group), mice in the CS-AKI model group (CS group) exhibited significantly elevated serum

levels of BUN and SCr at 24 h after decompression (**Fig. EV3B-C**), along with a marked reduction in GFR (**Fig. EV3D-F**). Histopathological examination revealed typical renal injuries, including cast formation, brush border detachment, and glomerular shrinkage (**Fig. EV3G-H**), collectively indicating successful establishment of the CS-AKI mouse model. Immediately after decompression, CS-AKI mice received an intraperitoneal injection of 10 mg/kg GST-Hpβ fusion protein (CS+GST-Hpβ group). Compared with the CS group, the CS+GST-Hpβ group showed significantly decreased serum BUN and SCr levels (**Fig. EV3B-C**) and a marked recovery of GFR (**Fig. EV3D-F**), indicating improved renal function. Moreover, both the renal injury score and the expression level of the kidney tubular injury marker NGAL were significantly reduced in the CS+GST-Hpβ group (**Fig. EV3G-J**). Notably, Mb accumulation in the renal tubules was also markedly attenuated (**Fig. EV3I, K**). Collectively, these results demonstrate that GST-Hpβ treatment significantly ameliorates renal dysfunction and reduces kidney injury in glycerol-induced non-traumatic RM-AKI mice or CS-AKI mice."

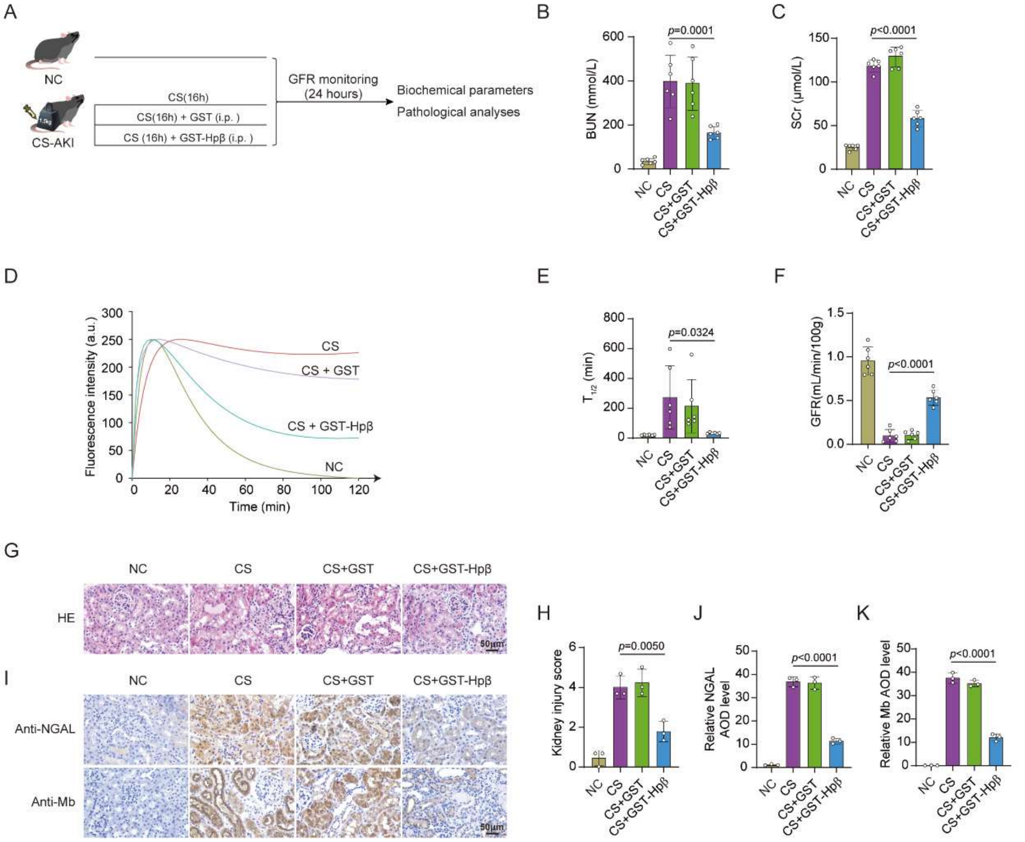

Figure EV3. Therapeutic effects of GST-Hpβ on CS-AKI mice and pathological examination of major organs in glycerol-induced RM-AKI mice.

(A) Experimental design of GST-Hpβ fusion protein treatment for CS-AKI mice. Mice were subjected to 1.5 kg of compression for 16 hours, and GST-Hpβ was injected immediately after decompression. Kidney function in the mice was tested 24 hours later, blood samples were collected for biochemical examination, and tissue samples were collected for pathological analysis.

(B-C) Blood biochemical analysis of the concentrations of BUN (**B**) and SCr (**C**) in serum in NC and CS-AKI mice (n = 6 per group for biological replicates).

(D) Representative image of transcutaneous disappearance curves of Inulin-FITC excretion after 24 h in i.p. injection with 10 mg/kg GST-Hpβ of CS-AKI mice.

(E) The excretion half-life (T1/2) of Inulin-FITC in NC, CS, CS+GST, and CS+GST-Hpβ groups of mice (n = 6 per group for biological replicates).

(F) GFR of mice in NC, CS, CS+GST, and CS+GST-Hpβ groups was measured at 24h (n = 6 per group for biological replicates).

(G) HE staining assesses renal tubular injury severity in selected NC, CS, CS+GST, and CS+GST-Hpβ groups.

(H) Blinded kidney injury score of the selected groups of mice in the image of (G) (n = 3 per group for biological replicates).

(I) Representative IHC staining images of Mb and NGAL expression in kidney tissues of NC, CS, CS+GST, and CS+GST-Hpβ groups.

(J-K) Quantification of the integrated optical density (IOD) of NGAL (J) and Mb (K) in the image of (I) (n = 3 per group for biological replicates).

Figure 3 G H. Again, I do not understand how we go from 6 to 3 replicates: how many mice were ultimately analyzed in each group? Regarding the absence of variance, I was referring to panels 3 B, C, D, E, where under certain conditions, the distribution of values is zero.

Response: We sincerely apologize for any confusion caused by the data presentation in our revised manuscript. In Figure 3, four experimental groups were included: Saline, Gly, Gly+GST, and Gly+GST-Hpβ, with n = 6 mice per group. For serum biochemical

analyses (SCr and BUN) and qPCR assays (*Kim-1*, *Ngal*), all 6 mice per group were analyzed, and the data are presented as individual points in Fig. 3B-E. For glomerular filtration rate (GFR) measurements shown in Fig. 3F-H, we randomly selected 3 out of the 6 mice per group for these assays. Thus, the sample size for GFR data is n = 3 biological replicates, as clearly indicated in the Figure legend. This approach is consistent with established practices in rodent GFR measurement studies, where smaller subset sampling is often employed due to equipment limitations. We appreciate the reviewer's careful observation. In the first version of Fig. 3G-H, we presented data from 3 biological replicates, each with 2 technical replicates, yielding 6 data points per group. Following the reviewer's valuable suggestion to increase data reproducibility, we repeated the experiments with 3 independent biological replicates (without technical replication) in the revised version (V2). This change was implemented to ensure that each data point represents a true biological replicate, thereby enhancing the statistical rigor and reproducibility of our findings. According to the editor's suggestion, the results from all biological replicates (from both the initial submission and the revised version) should be presented. Therefore, in this round of revisions, Fig. 3G-H shows data from six biological replicates: three from the initial submission and three from the V2 revision.

We have carefully re-examined the raw data for SCr, BUN, *Kim-1*, and *Ngal* (n = 6 per group) in Fig. 3B-E. Tables 1-4 (provided as source data) display the complete dataset, including individual values, averages, and standard deviations (SD). These tables confirm that variability exists among replicates in all groups.

Regarding the specific concern about Figure 3E (*Ngal* mRNA expression in the Gly+GST-Hpβ treatment group), we acknowledge that the bar graph appears compressed. This is because the mean value in the treatment group is approximately 2 (relative to the control), with a standard deviation (SD) of ~0.4, while the y-axis scale extends to 40 to accommodate the high values in the model group. This scaling choice, while necessary to display all data, visually minimizes the apparent variance in the low-expression groups. We presented individual data points for all panels in Fig. 3B-E, allowing direct visualization of the data distribution.

For the qPCR analyses presented in Fig. 3D-E, we employed the standard $2^{-\Delta\Delta Ct}$

method for relative quantification. In this widely accepted approach, the average of the control group (Saline) is set to 1, and all experimental groups are normalized relative to this control. Consequently, the Saline group shows an average of 1 and a standard deviation of 0, as normalization is performed on the group mean rather than on individual samples. This normalization strategy is standard practice in qPCR data analysis and has been extensively documented in the literature [1-6].

References:

[1] Fung E, Tang SM, Canner JP, Morishige K, Arboleda-Velasquez JF, Cardoso AA, Carlesso N, Aster JC, Aikawa M. Delta-like 4 induces notch signaling in macrophages: implications for inflammation. Circulation. 2007.115(23):2948-56. PMID: 17533181.

[2] Zheng J, Li Y, Guan H, Zhang J, Tan H. Enhancement of neomycin production by engineering the entire biosynthetic gene cluster and feeding key precursors in Streptomyces fradiae CGMCC 4.576. Appl Microbiol Biotechnol. 2019.103(5):2263-2275. PMID: 30685809.

[3] Pagie S, Gérard N, Charreau B. Notch signaling triggered via the ligand DLL4 impedes M2 macrophage differentiation and promotes their apoptosis. Cell Commun Signal. 2018.16(1):4. PMID: 29321062.

[4] He T, Wang M, Cao X, Cheng A, Wu Y, Yang Q, Liu M, Zhu D, Jia R, Chen S, Sun K, Zhao X, Chen X. Molecular characterization of duck enteritis virus UL41 protein. Virol J. 2018.15(1):12. PMID: 29334975.

[5] Sangphech N, Keawvilai P, Palaga T. Notch signaling increases PPARγ protein stability and enhances lipid uptake through AKT in IL-4-stimulated THP-1 and primary human macrophages. FEBS Open Bio. 2020.10(6):1082-1095. PMID: 32274896.

[6] Mohlin S, Kerosuo L. In Vitro Maintenance of Multipotent Neural Crest Stem Cells as Crestospheres. Methods Mol Biol. 2019.2002:1-11. PMID: 30159826.

Table 1. Original data of the Scr concentration detected by blood biochemistry in **Fig. 3B**

**Scr Concentration (μmol/L)**	**Biological replicate**						**Average**	**SD**
Saline	42.48	39.65	29.45	34.02	32.57	54.00	38.70	8.88
Gly	285.16	538.06	432.82	337.35	450.10	385.75	404.87	89.24
Gly+GST-ip	324.84	423.26	226.05	324.27	389.20	406.03	348.94	73.07
Gly+GST-Hpβ 10mg/kg-ip	71.43	41.95	58.49	45.65	53.24	44.26	52.50	11.14
Gly+GST-Hpβ 20mg/kg-ip	35.65	75.63	24.34	71.30	48.07	52.42	51.24	19.88
Gly+GST-Hpβ 40mg/kg-ip	31.38	28.14	63.68	40.37	56.79	39.41	43.30	14.09
Gly+GST-iv	262.23	297.60	223.01	257.96	260.58	385.74	281.19	56.41
Gly+GST-Hpβ 10mg/kg-iv	38.44	109.88	70.94	66.80	58.30	57.36	66.95	23.83
Gly+GST-Hpβ 20mg/kg-iv	36.80	48.30	51.00	38.98	60.83	40.93	46.14	9.05
Gly+GST-Hpβ 40mg/kg-iv	71.32	41.78	55.52	48.81	44.54	60.39	53.73	11.03

Table 2. Original data of the BUN concentration detected by blood biochemistry in **Fig. 3C**

**BUN Concentration (mmol/L)**	**Biological replicate**						**Average**	**SD**
Saline	10.00	20.02	18.55	16.57	15.92	18.21	16.55	3.52
Gly	125.13	108.31	136.54	117.62	128.91	132.57	124.85	10.38
Gly+GST-ip	97.07	87.63	90.25	99.23	85.19	89.29	91.44	5.51
Gly+GST-Hpβ 10mg/kg-ip	9.77	19.56	18.76	17.89	15.64	18.82	16.74	3.67
Gly+GST-Hpβ 20mg/kg-ip	17.53	16.85	18.52	21.57	18.88	16.58	18.32	1.83
Gly+GST-Hpβ 40mg/kg-ip	22.50	20.65	21.10	23.60	21.38	22.78	22.00	1.13
Gly+GST-iv	121.61	123.94	126.72	129.22	129.06	126.50	126.18	2.96
Gly+GST-Hpβ 10mg/kg-iv	25.00	16.66	27.59	28.87	21.20	29.33	24.78	4.98
Gly+GST-Hpβ 20mg/kg-iv	28.75	20.83	20.37	26.69	19.48	22.11	23.04	3.78
Gly+GST-Hpβ 40mg/kg-iv	22.93	23.96	31.67	27.56	23.49	24.36	25.66	3.36

Table 3. Original data of the *Kim-1* mRNA level detected by qPCR in **Fig. 3D**

**Relative *Kim-1* mRNA level**	**Biological replicate**						**Average**	**SD**
Saline	1.00	1.00	1.00	1.00	1.00	1.00	1.00	0.00
Gly	122.39	93.63	113.91	120.17	98.74	126.59	112.57	13.44
Gly+GST-ip	96.79	82.80	81.11	102.05	78.43	87.42	88.10	9.39
Gly+GST-Hpβ 10mg/kg-ip	12.16	9.51	10.09	12.82	9.50	10.64	10.79	1.40
Gly+GST-Hpβ 20mg/kg-ip	14.45	11.16	15.56	15.85	16.54	11.15	14.12	2.39
Gly+GST-Hpβ 40mg/kg-ip	24.50	23.98	20.32	25.84	24.73	20.50	23.31	2.33
Gly+GST-iv	109.77	115.76	121.35	130.70	121.31	123.40	120.38	7.09
Gly+GST-Hpβ 10mg/kg-iv	11.28	12.60	12.10	14.14	12.46	12.55	12.52	0.93
Gly+GST-Hpβ 20mg/kg-iv	24.21	23.66	25.02	26.93	24.75	25.71	25.05	1.16
Gly+GST-Hpβ 40mg/kg-iv	32.68	31.19	32.70	30.87	29.88	34.67	32.00	1.70

Table 4. Original data of the *Ngal* mRNA level detected by qPCR in **Fig. 3E**

**Relative *Ngal* mRNA level**	**Biological replicate**						**Average**	**SD**
Saline	1.00	1.00	1.00	1.00	1.00	1.00	1.00	0.00
Gly	31.78	30.63	39.31	23.60	22.25	23.20	28.46	6.68
Gly+GST-ip	23.16	18.04	23.72	29.27	30.44	23.61	24.71	4.53
Gly+GST-Hpβ 10mg/kg-ip	1.11	1.80	1.10	1.40	2.19	1.36	1.49	0.43
Gly+GST-Hpβ 20mg/kg-ip	1.13	1.43	1.39	1.45	1.84	1.79	1.51	0.27
Gly+GST-Hpβ 40mg/kg-ip	1.08	1.46	2.15	1.41	1.86	2.81	1.80	0.62
Gly+GST-iv	21.54	20.04	24.12	33.20	31.30	36.62	27.80	6.81
Gly+GST-Hpβ 10mg/kg-iv	1.17	1.50	1.83	1.34	1.40	1.04	1.38	0.28
Gly+GST-Hpβ 20mg/kg-iv	1.73	2.24	2.67	1.92	2.31	1.75	2.10	0.37
Gly+GST-Hpβ 40mg/kg-iv	2.21	2.70	3.20	2.49	2.66	3.38	2.77	0.44

In Figure 6B, the in vivo imaging analyses do not show the Gly+(Cy5+GST+Hpbeta) condition: why?

Response: We thank the reviewer for this question. In our experiments, the Gly+Cy5-GST-Hpβ group was indeed included and is shown in Fig. 6B (rightmost lane). Specifically, the GST-Hpβ fusion protein was covalently conjugated with the Cy5 fluorescent dye, and the purified Cy5-GST-Hpβ protein was prepared by BioSS Biotechnology Co., Ltd. (Beijing, China). One day prior to the imaging experiment, the fur of each mouse was depilated from the neck to the ankle to expose the skin. During the experiment, mice were anesthetized, and those in the Gly+Cy5-GST-Hpβ group received an intramuscular injection of 50% glycerol (8 mL/kg) into the bilateral hind limbs, immediately followed by an intravenous tail vein injection of 200 μL Cy5-GST-Hpβ fusion protein (10 mg/kg). Fluorescence imaging was performed using a small animal live imaging system (Tanon ABL X5, Shanghai, China) at 0, 1, 3, 24, 48, and 72 h post-administration. Bright-field and dark-field images were overlaid, and regions of interest (ROI) encompassing the entire mouse were analyzed using the imaging system's analysis software (Tanon, version 2.0.1) to quantify whole-body fluorescence intensity.

We understand that the reviewer may be asking why the Gly+Cy5-GST condition (i.e., Cy5-labeled GST protein without the Hpβ moiety) was not included. We want to clarify the rationale behind our experimental design.

The primary objective of the *in vivo* imaging experiment shown in Fig. 6B was to show the preliminary tissue distribution and metabolic fate of the GST-Hpβ fusion protein between healthy (Saline) and diseased (RM-AKI) mice. Specifically, we aimed to determine whether excess myoglobin (Mb) in the circulation of RM-AKI mice alters the distribution and clearance kinetics of the GST-Hpβ fusion protein. Therefore, we included the following experimental groups:

Saline+Cy5-GST-Hpβ group: To establish the preliminary tissue distribution and metabolic profile of the fusion protein in healthy animals.

Gly+Cy5-GST-Hpβ group: To preliminarily assess how the disease state (RM-AKI) affects the tissue distribution and metabolism of GST-Hpβ.

Free Cy5 dye controls (Saline+Cy5 group, Gly+Cy5 group): To serve as

background controls for each condition.

The "Gly+Cy5-GST" group (Cy5-labeled GST tag without Hpβ) was not included because the GST tag alone lacks myoglobin-binding function, which is the core therapeutic mechanism of our fusion protein. Including this control group would not address our central question—the preliminary metabolism and distribution of the GST-Hpβ fusion protein in healthy (Saline) and RM-AKI mice—but rather tests an irrelevant control protein. The experimental design used in this study has been widely applied in animal rescue studies of protein therapeutics for pharmacokinetics and biodistribution analysis [1-2].

References:

[1] Shi D, Liu W, Gao Y, Li X, Huang Y, Li X, James TD, Guo Y, Li J. Photoactivatable senolysis with single-cell resolution delays aging. Nat Aging. 2023.3(3):297-312. PMID: 37118423.

[2] Jin C, Xu X, Yao N, Zhang H, Chen C, Zeng L, Liu Z, Lei L, Cui S, Wen C, Shi L. Prodrug nanoplatform for triggering ferroptosis to eliminate senescent cells in age-associated pathologies. Nat Commun. 2025.17(1):630. PMID: 41390693.

We are grateful to the reviewer for the time and expertise, and we appreciate the thoughtful feedback that has helped us improve our work.

28th Apr 2026

Dear Prof. Gong,

Thank you for the submission of your revised manuscript to EMBO Molecular Medicine. I am pleased to inform you that we will be able to accept your manuscript pending the following final amendments:

1) Please address referee #3 remaining concerns by discussion.
2) Source data should be made freely accessible.
3) Synopsis:
- Synopsis image: Please upload the image as a separate, high-resolution jpeg file, exactly 550 px-wide x 300 pixels high.
- Please check your synopsis text and image before submission with your revised manuscript. Please be aware that in the proof stage minor corrections only are allowed (e.g., typos).
4) As part of the EMBO Publications transparent editorial process (see our Editorial at http://embomolmed.embopress.org/content/2/9/329), EMBO Molecular Medicine will publish online a Review Process File (RPF) to accompany accepted manuscripts. This file will be published in conjunction with your paper and will include the anonymous referee reports, your point-by-point response and all pertinent correspondence relating to the manuscript. Let us know if you want to remove or not any figures from it prior to publication. Please note that the Authors checklist will be published at the end of the RPF.
5) Please provide a point-by-point letter INCLUDING my comments as well as the reviewer's reports and your detailed responses (as Word file).

I look forward to reading a new revised version of your manuscript as soon as possible.

Yours sincerely,

Zeljko Durdevic

Zeljko Durdevic
Senior Editor
EMBO Molecular Medicine

*** Instructions to submit your revised manuscript ***

*** PLEASE NOTE *** As part of the EMBO Publications transparent editorial process initiative (see our Editorial at https://www.embopress.org/doi/pdf/10.1002/emmm.201000094), EMBO Molecular Medicine will publish online a Review Process File to accompany accepted manuscripts.

In the event of acceptance, this file will be published in conjunction with your paper and will include the anonymous referee reports, your point-by-point response and all pertinent correspondence relating to the manuscript. If you do NOT want this file to be published, please inform the editorial office at contact@embomolmed.org.

*****

When preparing your revised manuscript, please refer to our guidelines: https://link.springer.com/journal/44321/submission-guidelines#cms-Revised-submissions. We perform an initial quality control of all revised manuscripts before re-review; failure to include requested items will delay the evaluation of your revision.

We require:

1) A .docx formatted version of the manuscript text (including legends for main figures, EV figures and tables). Please make sure that the changes are highlighted to be clearly visible.

2) Individual production quality figure files as .eps, .tif, .jpg (one file per figure). For guidance, download the 'Figure Guide PDF': https://media.springernature.com/original/springer-cms/rest/v1/content/27825798/data/v1.

3) A .docx formatted letter INCLUDING the reviewers' reports and your detailed point-by-point responses to their comments. As part of the EMBO Press transparent editorial process, the point-by-point response is part of the Review Process File (RPF), which will be published alongside your paper.

4) A complete author checklist, which you can download from our author guidelines. Please insert information in the checklist that is also reflected in the manuscript. The completed author checklist will also be part of the RPF.

5) Please note that all corresponding authors are required to supply an ORCID ID for their name upon submission of a revised manuscript.

6) It is mandatory to include a 'Data Availability' section after the Materials and Methods. Before submitting your revision, primary datasets produced in this study need to be deposited in an appropriate public database, and the accession numbers and database listed under 'Data Availability'. Please remember to provide a reviewer password if the datasets are not yet public.

In case you have no data that requires deposition in a public database, please state so in this section. Note that the Data Availability Section is restricted to new primary data that are part of this study.

7) For data quantification: please specify the name of the statistical test used to generate error bars and P values, the number (n) of independent experiments (specify technical or biological replicates) underlying each data point and the test used to calculate p-values in each figure legend. The figure legends should contain a basic description of n, P and the test applied. Graphs must include a description of the bars and the error bars (s.d., s.e.m.).

8) At EMBO Press we ask authors to provide source data for the main manuscript figures. You will receive a separate email with instructions for providing source data with your revised manuscript, including how to upload and organize the files.

9) Our journal encourages inclusion of *data citations in the reference list* to directly cite datasets that were re-used and obtained from public databases. Data citations in the article text are distinct from normal bibliographical citations and should directly link to the database records from which the data can be accessed. In the main text, data citations are formatted as follows: "Data ref: Smith et al, 2001" or "Data ref: NCBI Sequence Read Archive PRJNA342805, 2017". In the Reference list, data citations must be labeled with "[DATASET]". A data reference must provide the database name, accession number/identifiers and a resolvable link to the landing page from which the data can be accessed at the end of the reference.

10) We replaced Supplementary Information with Expanded View (EV) Figures and Tables that are collapsible/expandable online. A maximum of 5 EV Figures can be typeset. EV Figures should be cited as 'Figure EV1, Figure EV2" etc... in the text and their respective legends should be included in the main text after the legends of regular figures.

- For the figures that you do NOT wish to display as Expanded View figures, they should be bundled together with their legends in a single PDF file called *Appendix*, which should start with a short Table of Content. Appendix figures should be referred to in the main text as: "Appendix Figure S1, Appendix Figure S2" etc.

- Additional Tables/Datasets should be labeled and referred to as Table EV1, Dataset EV1, etc. Legends have to be provided in a separate tab in case of .xls files. Alternatively, the legend can be supplied as a separate text file (README) and zipped together with the Table/Dataset file.

11) The paper explained: EMBO Molecular Medicine articles are accompanied by a summary of the articles to emphasize the major findings in the paper and their medical implications for the non-specialist reader. Please provide a draft summary of your article highlighting

- the medical issue you are addressing,

- the results obtained and

- their clinical impact.

This may be edited to ensure that readers understand the significance and context of the research. Please refer to any of our published articles for an example.

12) Author contributions: You will be asked to provide CRediT (Contributor Role Taxonomy) terms in the submission system. These replace a narrative author contribution section in the manuscript.

13) A Conflict of Interest statement should be provided in the main text.

14) Every published paper includes a 'Synopsis' to further enhance discoverability. Synopses are displayed on the journal webpage and are freely accessible to all readers. They include a short stand first (maximum of 300 characters, including space) as well as 2-5 one-sentences bullet points that summarizes the paper. Please write the bullet points to summarize the key NEW findings. They should be designed to be complementary to the abstract - i.e. not repeat the same text. We encourage inclusion of key acronyms and quantitative information (maximum of 30 words / bullet point). Please use the passive voice. Please attach these in a separate file or send them by email, we will incorporate them accordingly.

Please also suggest a striking image or visual abstract to illustrate your article as a PNG file 550 px wide x 300 px high.

15) Include a Reagents and Tools Table as part of the Methods section, which can be downloaded from our author guidelines.

EMBO Molecular Medicine has a "scooping protection" policy, whereby similar findings that are published by others during review or revision are not a criterion for rejection. Should you decide to submit a revised version, I do ask that you get in touch after three months if you have not completed it, to update us on the status.

*Additional important information regarding Figures

Each figure should be given in a separate file and should have the following resolution:
Graphs 800-1,200 DPI
Photos 400-800 DPI
Colour (only CMYK) 300-400 DPI"

Figures are not edited by the production team. All lettering should be the same size and style; figure panels should be indicated by capital letters (A, B, C etc). Gridlines are not allowed except for log plots. Figures should be numbered in the order of their appearance in the text with Arabic numerals. Each Figure must have a separate legend and a caption is needed for each panel.

*Additional important information regarding figures and illustrations can be found at
https://media.springernature.com/original/springer-cms/rest/v1/content/27825798/data/v1

***** Reviewer's comments *****

Referee #3 (Remarks for Author):

The addition of a second model allows for at least a partial generalization of the efficacy of the GST-Hpβ fusion protein.
The major limitation is that the treatment is preventive rather than curative; that is, it is administered at the same time as the injury causing rhabdomyolysis, which never occurs under normal clinical conditions, where there is always a time lag between the diagnosis of rhabdomyolysis/acute kidney injury and medical intervention.
Furthermore, it would have been appropriate to compare treatment with the GST-Hpβ fusion protein against standard of care (hyperhydration)

Point-by-point responses

***** Reviewer's comments *****

Referee #3 (Remarks for Author):

The addition of a second model allows for at least a partial generalization of the efficacy of the GST-Hpβ fusion protein.

Response: Thank you for recognizing the therapeutic effect of GST-HPβ in the dual models of rhabdomyolysis-induced acute kidney injury (glycerol-induced non-traumatic RM-AKI or CS-AKI). We also appreciate the reviewer's constructive suggestion to supplement the experiments regarding the therapeutic effect in the CS-AKI model.

The major limitation is that the treatment is preventive rather than curative; that is, it is administered at the same time as the injury causing rhabdomyolysis, which never occurs under normal clinical conditions, where there is always a time lag between the diagnosis of rhabdomyolysis/acute kidney injury and medical intervention.

Furthermore, it would have been appropriate to compare treatment with the GST-Hpβ fusion protein against standard of care (hyperhydration)

Response: We thank the reviewers for pointing out the limitations of this study regarding the timing of administration from a clinical practice perspective, as well as for the suggestion to compare with the standard of care (hyperhydration). We have added a discussion on the timing of administration and fluid resuscitation in the Limitations paragraph of the Discussion section of the paper, as detailed below (underline highlight):

"Our study has limitations. Thirdly, the tandem multi-beta-subunit of Hp has a large molecular weight, binds more free Mb, and may have a more significant treatment effect than the GST-Hpβ fusion protein in RM-AKI. In fact, this research only completed the proof of concept for treating RM-AKI with the Hpβ fusion protein. Before clinical translation, additional pharmacokinetic and safety studies are required, including a thorough assessment of long-term toxicity and immune responses. Moreover, given that fluid resuscitation is the standard clinical treatment for preserving

renal function during rhabdomyolysis, subsequent evaluation of GST-Hpβ or recombinant tandem multi-beta-subunit of Hp proteins, in combination with or compared to standard fluid resuscitation, will be critical for defining their clinical positioning. Fourth, under normal clinical conditions, the timing of administration in this study may be more preventive than therapeutic. Since there is always a time lag between the diagnosis of RM-AKI and medical intervention in clinical practice, it is difficult to administer treatment simultaneously with rhabdomyolysis injury. However, in scenarios with a high incidence of traumatic rhabdomyolysis-AKI (CS-AKI), such as earthquakes, immediately injecting Hpβ proteins after rescuing long-term buried victims may be an effective way to reduce the occurrence of AKI and lower the mortality."

At last, we sincerely thank Referee #3 for the time and constructive suggestions for our revisions.

11th May 2026

Dear Prof. Gong,

We are pleased to inform you that your manuscript is accepted for publication and is now being sent to our publisher to be included in the next available issue of EMBO Molecular Medicine.

Your manuscript will be processed for publication by EMBO Press. It will be copy edited and you will receive page proofs prior to publication. Please note that you will be contacted by Springer Nature Author Services to complete licensing and payment information.

You may qualify for financial assistance for your publication charges - either via a Springer Nature fully open access agreement or an EMBO initiative. Check your eligibility: https://link.springer.com/journal/44321/how-to-publish-with-us

Should you be planning a Press Release on your article, please get in contact with embo_production@springernature.com as early as possible in order to coordinate publication and release dates.

If you have any questions, please do not hesitate to contact the Editorial Office. Thank you for your contribution to EMBO Molecular Medicine.

Yours sincerely,
Zeljko Durdevic

Zeljko Durdevic
Senior Editor
EMBO Molecular Medicine

>>> Please note that it is EMBO Molecular Medicine policy for the transcript of the editorial process (containing referee reports and your response letter) to be published as an online supplement to each paper. If you do NOT want this, you will need to inform the Editorial Office via email immediately. More information is available here: https://link.springer.com/partners/embo-press/editorial-policies#Peer%20review

EMBO Press Author Checklist

Corresponding Author Name: Shuquan Rao, Yanhua Gong
Journal Submitted to: EMBO Molecular Medicine
Manuscript Number: EMM-2025-22760-V3

USEFUL LINKS FOR COMPLETING THIS FORM
The EMBO Journal - Author Guidelines
EMBO Reports - Author Guidelines
Molecular Systems Biology - Author Guidelines
EMBO Molecular Medicine - Author Guidelines

Reporting Checklist for Life Science Articles (updated January
This checklist is adapted from Materials Design Analysis Reporting (MDAR) Checklist for Authors. MDAR establishes a minimum set of requirements in transparent reporting in the life sciences (see Statement of Task: 10.31222/osf.io/9sm4x). Please follow the journal's guidelines in preparing your
Please note that a copy of this checklist will be published alongside your article.

Abridged guidelines for figures
1. Data
The data shown in figures should satisfy the following conditions:
➞ the data were obtained and processed according to the field's best practice and are presented to reflect the results of the experiments in an accurate and unbiased manner.
➞ ideally, figure panels should include only measurements that are directly comparable to each other and obtained with the same assay.
➞ plots include clearly labeled error bars for independent experiments and sample sizes. Unless justified, error bars should not be shown for technical
➞ if n<5, the individual data points from each experiment should be plotted. Any statistical test employed should be justified.
➞ Source Data should be included to report the data underlying figures according to the guidelines set out in the authorship guidelines on Data

2. Captions
Each figure caption should contain the following information, for each panel where they are relevant:
➞ a specification of the experimental system investigated (eg cell line, species name).
➞ the assay(s) and method(s) used to carry out the reported observations and measurements.
➞ an explicit mention of the biological and chemical entity(ies) that are being measured.
➞ an explicit mention of the biological and chemical entity(ies) that are altered/varied/perturbed in a controlled manner.
➞ the exact sample size (n) for each experimental group/condition, given as a number, not a range;
➞ a description of the sample collection allowing the reader to understand whether the samples represent technical or biological replicates (including how many animals, litters, cultures, etc.).
➞ a statement of how many times the experiment shown was independently replicated in the laboratory.
➞ definitions of statistical methods and measures:
- common tests, such as t-test (please specify whether paired vs. unpaired), simple χ2 tests, Wilcoxon and Mann-Whitney tests, can be unambiguously identified by name only, but more complex techniques should be described in the methods section;
- are tests one-sided or two-sided?
- are there adjustments for multiple comparisons?
- exact statistical test results, e.g., P values = x but not P values < x;
- definition of 'center values' as median or average;
- definition of error bars as s.d. or s.e.m.

Please complete ALL of the questions below.
Select "Not Applicable" only when the requested information is not relevant for your study.

Materials

Newly Created Materials	Information included in the manuscript?	In which section is the information available? (Reagents and Tools Table, Materials and Methods, Figures, Data Availability Section)
New materials and reagents need to be available; do any restrictions apply?	Yes	Methods section, Line 521-528

Antibodies	Information included in the manuscript?	In which section is the information available? (Reagents and Tools Table, Materials and Methods, Figures, Data Availability Section)
For **antibodies** provide the following information:<br>- Commercial antibodies: RRID (if possible) or supplier name, catalogue number and or/clone number<br>- Non-commercial: RRID or citation	Yes	Methods section, Line 450-454, 487-503

DNA and RNA sequences	Information included in the manuscript?	In which section is the information available? (Reagents and Tools Table, Materials and Methods, Figures, Data Availability Section)
**Short novel DNA or RNA including primers, probes**: provide the sequences.	Yes	Table EV1. Primer sequences for qPCR

Cell materials	Information included in the manuscript?	In which section is the information available? (Reagents and Tools Table, Materials and Methods, Figures, Data Availability Section)
**Cell lines:** Provide species information, strain. Provide accession number in repository **OR** supplier name, catalog number, clone number, and/**OR** RRID.	Yes	Methods section, Line 559-564
**Primary cultures:** Provide species, strain, sex of origin, genetic modification status.	Not Applicable	n/a
Report if the cell lines were recently **authenticated** (e.g., by STR profiling) and tested for mycoplasma contamination.	Yes	Methods section, Line 563-564

Experimental animals	Information included in the manuscript?	In which section is the information available? (Reagents and Tools Table, Materials and Methods, Figures, Data Availability Section)
**Laboratory animals or Model organisms:** Provide species, strain, sex, age, genetic modification status. Provide accession number in repository **OR** supplier name, catalog number, clone number, **OR** RRID.	Yes	Methods section, Line 409
**Animal observed in or captured from the field:** Provide species, sex, and age where possible.	Not Applicable	n/a
Please detail **housing and husbandry conditions**.	Yes	Methods section, Line 409-410

Plants and microbes	Information included in the manuscript?	In which section is the information available? (Reagents and Tools Table, Materials and Methods, Figures, Data Availability Section)
**Plants:** provide species and strain, ecotype and cultivar where relevant, unique accession number if available, and source (including location for collected wild specimens).	Not Applicable	n/a
**Microbes:** provide species and strain, unique accession number if available, and source.	Not Applicable	n/a

Human research participants	Information included in the manuscript?	In which section is the information available? (Reagents and Tools Table, Materials and Methods, Figures, Data Availability Section)
If collected and within the bounds of privacy constraints report on age, sex and gender or ethnicity for all study participants.	Not Applicable	n/a

Core facilities	Information included in the manuscript?	In which section is the information available? (Reagents and Tools Table, Materials and Methods, Figures, Data Availability Section)
If your work benefited from core facilities, was their service mentioned in the acknowledgments section?	Not Applicable	n/a

Design

Study protocol	Information included in the manuscript?	In which section is the information available? (Reagents and Tools Table, Materials and Methods, Figures, Data Availability Section)
If study protocol has been **pre-registered, provide DOI in the manuscript**. For clinical trials, provide the trial registration number **OR** cite DOI.	Not Applicable	n/a
Report the **clinical trial registration number** (at ClinicalTrials.gov or equivalent), where applicable.	Not Applicable	n/a

Laboratory protocol	Information included in the manuscript?	In which section is the information available? (Reagents and Tools Table, Materials and Methods, Figures, Data Availability Section)
Provide DOI OR other citation details if **external detailed step-by-step protocols** are available.	Not Applicable	n/a

Experimental study design and statistics	Information included in the manuscript?	In which section is the information available? (Reagents and Tools Table, Materials and Methods, Figures, Data Availability Section)
Include a statement about **sample size** estimate even if no statistical methods were used.	Yes	Methods section, Line 594-596
Were any steps taken to minimize the effects of subjective bias when allocating animals/samples to treatment (e.g. **randomization procedure**)? If yes, have they been described?	Yes	Methods section, Line 410-411, 591-592
Include a statement about **blinding** even if no blinding was done.	Yes	Methods section, Line 592
Describe **inclusion/exclusion criteria** if samples or animals were excluded from the analysis. Were the criteria pre-established?<br><br>If sample or data points were omitted from analysis, report if this was due to attrition or intentional exclusion and provide justification.	Yes	Methods section, Line 591
For every figure, are **statistical tests** justified as appropriate? Do the data meet the assumptions of the tests (e.g., normal distribution)? Describe any methods used to assess it. Is there an estimate of variation within each group of data? Is the variance similar between the groups that are being statistically compared?	Yes	Methods section, Line 594-598

Sample definition and in-laboratory replication	Information included in the manuscript?	In which section is the information available? (Reagents and Tools Table, Materials and Methods, Figures, Data Availability Section)
In the figure legends: state number of times the experiment was **replicated** in laboratory.	Yes	Yes, in figure legends of Fig.1, Fig.3-6, Fig. EV1-4
In the figure legends: define whether data describe **technical or biological replicates**.	Yes	Yes, in figure legends of Fig.1, Fig.3-6, Fig. EV1-4

Ethics

Ethics	Information included in the manuscript?	In which section is the information available? (Reagents and Tools Table, Materials and Methods, Figures, Data Availability Section)
Studies involving **human participants**: State details of **authority granting ethics approval** (IRB or equivalent committee(s), provide reference number for approval.	Not Applicable	n/a
Studies involving **human participants**: Include a statement confirming that **informed consent** was obtained from all subjects and that the experiments conformed to the principles set out in the WMA Declaration of Helsinki and the Department of Health and Human Services Belmont Report.	Not Applicable	n/a
Studies involving **human participants:** For publication of **patient photos**, include a statement confirming that consent to publish was obtained.	Not Applicable	n/a
Studies involving experimental **animals**: State details of **authority granting ethics approval** (IRB or equivalent committee(s), provide reference number for approval. Include a statement of compliance with ethical regulations.	Yes	Methods section, Line 418-419
Studies involving **specimen and field samples:** State if relevant **permits** obtained, provide details of authority approving study; if none were required, explain why.	Not Applicable	n/a

Dual Use Research of Concern (DURC)	Information included in the manuscript?	In which section is the information available? (Reagents and Tools Table, Materials and Methods, Figures, Data Availability Section)
Could your study fall under dual use research restrictions? Please check biosecurity documents and list of **select agents and toxins** (CDC): https://www.selectagents.gov/sat/list.htm	Not Applicable	n/a
If you used a select agent, is the security level of the lab appropriate and reported in the manuscript?	Not Applicable	n/a
If a study is subject to dual use research of concern regulations, is the name of the **authority granting approval and reference number** for the regulatory approval provided in the manuscript?	Not Applicable	n/a

Reporting

The MDAR framework recommends adoption of discipline-specific guidelines, established and endorsed through community initiatives. Journals have their own policy about requiring specific guidelines and recommendations to complement MDAR.

Adherence to community standards	Information included in the manuscript?	In which section is the information available? (Reagents and Tools Table, Materials and Methods, Figures, Data Availability Section)
State if relevant guidelines or checklists (e.g., **ICMJE, MIBBI, ARRIVE, PRISMA**) have been followed or provided.	Yes	Methods section, Line 418-419
For **tumor marker prognostic studies,** we recommend that you follow the **REMARK** reporting guidelines (see link list at top right). See author guidelines, under 'Reporting Guidelines'. Please confirm you have followed these guidelines.	Not Applicable	n/a
For **phase II and III randomized controlled trials**, please refer to the **CONSORT** flow diagram (see link list at top right) and submit the CONSORT checklist (see link list at top right) with your submission. See author guidelines, under 'Reporting Guidelines'. Please confirm you have submitted this list.	Not Applicable	n/a

Data Availability

Data availability	Information included in the manuscript?	In which section is the information available? (Reagents and Tools Table, Materials and Methods, Figures, Data Availability Section)
Have **primary datasets** been deposited according to the journal's guidelines (see 'Data Deposition' section) and the respective accession numbers provided in the Data Availability Section?	Not Applicable	n/a
Were **human clinical and genomic datasets** deposited in a public access-controlled repository in accordance to ethical obligations to the patients and to the applicable consent agreement?	Not Applicable	n/a
Are **computational models** that are central and integral to a study available without restrictions in a machine-readable form? Were the relevant accession numbers or links provided?	Not Applicable	n/a
If publicly available data were reused, provide the respective **data citations in the reference list**.	Not Applicable	n/a
